# Medicinal Plants for Mitigating Pain and Inflammatory-Related Conditions: An Appraisal of Ethnobotanical Uses and Patterns in South Africa

**DOI:** 10.3389/fphar.2021.758583

**Published:** 2021-10-22

**Authors:** Adeyemi O. Aremu, Srinivasa C. Pendota

**Affiliations:** ^1^ Indigenous Knowledge Systems Centre, Faculty of Natural and Agricultural Sciences, North-West University, Mmabatho, South Africa; ^2^ School of Life Sciences, University of KwaZulu-Natal Pietermaritzburg, Scottsville, South Africa

**Keywords:** ethnobotanical survey, folk medicine, headache, indigenous knowledge, rheumatism

## Abstract

In South Africa, traditional medicine remains the first point of call for a significant proportion of the population seeking primary healthcare needs. This is particularly important for treating common conditions including pain and inflammation which are often associated with many disease conditions. This review focuses on the analysis of the trend and pattern of plants used for mitigating pain and inflammatory-related conditions in South African folk medicine. An extensive search was conducted using various scientific databases and popular ethnobotanical literature focusing on South African ethnobotany. Based on the systematic analysis, 38 sources were selected to generate the inventory of 495 plants from 99 families that are considered as remedies for pain and inflammatory-related conditions (e.g., headache, toothache, backache, menstrual pain, and rheumatism) among different ethnic groups in South Africa. The majority (55%) of the 38 studies were recorded in three provinces, namely, KwaZulu-Natal, Limpopo, and Western Cape. In terms of the number of mentions, the most popular plants used for pain and inflammatory-related conditions in South Africa were *Ricinus communis* L. (10), *Aloe ferox* Mill. (8), *Pentanisia prunelloides* subsp. *latifolia* (Hochst.) Verdc. (8), *Dodonaea viscosa* Jacq var. *angustifolia* (L.f) Benth. (8), (L.) W.T.Aiton. (7) *Ruta graveolens* L. (7), and *Solanum aculeastrum* Dunal. (7). The top five plant families represented were Asteraceae (13%), Fabaceae (8%), Apocynaceae (4.3%), Asparagaceae (4%), and Lamiaceae (4%). An estimated 54% of the recorded plants were woody (trees and shrubs) in nature, while the leaves (27%) and roots (25%) were the most dominant plant parts. The use of plants for alleviating pain and inflammatory-related conditions remains popular in South African folk medicine. The lagging ethnobotanical information from provinces such as North West, Gauteng, and Free State remains a gap that needs to be pursued meticulously in order to have a complete country-wide database.

## Introduction

Inflammation is one of the most fundamental and pronounced protective reactions of an organism (Iwalewa et al., 2007; Medzhitov 2008; Kuprash and Nedospasov 2016; Kishore et al., 2019). It is regarded as a biological function which is triggered after the mechanical tissue disruption or from the responses by the presence of a physical, chemical, or biological agent in the body (Ahmed 2011; Ashley et al., 2012). Since ancient times, the complex and diverse patterns of inflammation development and their role in various (minor to major) disease conditions remain of great interest to researchers (Rocha e Silva 1978; Medzhitov 2008; Ashley et al., 2012; Bernstein et al., 2018; Hashemzaei et al., 2020). From a historical perspective (Rocha e Silva 1978), the main four signs of inflammation include redness (*rubor*), swelling (*tumor*), heat with (*calor*), and pain (*dolor*). In addition, the loss or disturbance of function (*functio laesa*) is considered the fifth sign (Kuprash and Nedospasov 2016). These aforementioned signs are considered as clinical signs of inflammation which are known to generally involve a sequence of events (Agyare et al., 2013). As a medical condition, pain is an enormous problem with an estimated 20% of adults suffering from this globally and 10% are newly diagnosed with chronic pain yearly (Goldberg and McGee 2011). Based on recent data (James et al., 2018), the Global Burden of Disease Study reaffirmed that the high prominence of pain and pain-related conditions remain the leading cause of disability and disease burden globally. From an epidemiological perspective, the importance of pain and related conditions cannot be overemphasized as it is known as a common, complex, and distressing problem that has a profound impact on individuals and society at large (Goldberg and McGee 2011; Mills et al., 2019).

Despite the existence of conventional drugs/medicines for pain, inflammation, and related conditions, the high risk of side effects and exorbitant cost remain a major deterrent to many people especially in developing and underdeveloped countries (Heinrich 2013; Sreekeesoon and Mahomoodally 2014; Bernstein et al., 2018; Kishore et al., 2019; Daniyal and Wang 2021). On this basis, research on alternative approaches especially pharmacological interventions has remained pertinent (Daniyal and Wang 2021). In sub-Saharan Africa, the rich biodiversity and relatively high level of plant endemism often translate to great dependence on botanicals for therapeutic purposes (Moyo et al., 2015; Alebie et al., 2017; Aumeeruddy and Mahomoodally 2020; Van Wyk 2020). An estimate of 4,576–5,000 plant species has been used as food and for the treatment of various diseases (Agyare et al., 2013; Van Wyk 2020). The use of plant-based remedies for the mitigation of pain, inflammation, and related conditions remains popular among different ethnic groups (Stark et al., 2013; Oguntibeju 2018; Daniyal and Wang 2021). As a result, the need for further research on plants with anti-inflammatory activities cannot be overemphasized (Agyare et al., 2013; Heinrich 2013; Oguntibeju 2018; Adebayo and Amoo 2019; Elgorashi and McGaw 2019).

From an ethnobotanical/ethnopharmacological context, the primary data generated from field studies are the foundation towards exploring plant resources for novel bioactive entities and herbal medicine for different diseases (Heinrich et al., 2018; Yeung et al., 2020). The importance of such knowledge is well recognized as they can contribute to improving human health and the fight against diseases on local and global levels (Heinrich 2013; Popović et al., 2016). South Africa is culturally diverse and divided into nine provinces with an estimate of over 55 million people. Across the different ethnic groups, the value of traditional medicine and the importance of medicinal plants is well recognized (Van Wyk 2002; Van Staden 2008; Van Wyk 2011). Currently, only a few research groups in South Africa have focused on ethnobotanical surveys despite the fragile nature and rapid disappearance of indigenous knowledge systems associated with the rich plant biodiversity (Viljoen et al., 2019). The aim of the current review is to analyze the existing literature on ethnobotanical studies/surveys, books, and grey literature that focused on plants used for pain, inflammation, and related conditions in South Africa. In addition to generating baseline data for future pharmacological and phytochemical investigations, this appraisal of literature is expected to identify the existing knowledge gap(s) and serve as an important reference for future research in the field.

## Methods

### Strategy for Literature Search

We conducted a detailed literature search by retrieving information from different scientific databases such as ScienceDirect, Scopus, and PubMed. The literature search (covering up till January 2021) was guided by the Preferred Reporting Items for Systematic Reviews and Meta-Analyses (PRISMA) statement (Moher et al., 2015). Keywords and phrases that were used included anti-inflammation, inflammation, pain, ethnobotany, ethnobotanical survey, and South Africa. These were used individually and in various combinations. In order to expand the generated data, we explored and included benchmark ethnobotanical books relevant to the South African context and which are currently available in the library of the North-West University, South Africa.

To generate the inventory/data for Table 1 and Table 2 as well as Supplementary Table S1, the inclusion criteria were that 1) the literature has ethnobotanical or ethnopharmacological context, and articles should be ethnobotanical field studies/surveys reporting on plant(s) with an indication as used for treating pain, inflammation, and related conditions, 2) the study location must be South Africa, 3) study must focus on plants, and 4) study must be written in English. On the other hand, the exclusion criteria were 1) articles with no scientific plant names, 2) review articles, and 3) articles focusing on animals and other natural resources used for treating pain, inflammation, and related conditions.

**TABLE 1:** Overview of literature documenting the use of medicinal plants for managing and treating pain and inflammatory-related conditions in South Africa.

	Author(s)	Province	Title/focus of the study	Number of plants
1	Arnold and Gulumian (1984)	Limpopo	Pharmacopoeia of traditional medicine in Venda	53
2	Bhat and Jacobs (1995)	Eastern Cape	Inventory of traditional herbal medicine in Transkei	14
3	Bhat (2013)	Eastern Cape	Plants of Xhosa people in the Transkei region	6
4	Bhat (2014)	Eastern Cape	Medicinal plants and traditional practices of Xhosa people in the Transkei region of Eastern Cape	13
5	Bruce (1975)	No restriction	Medicinal properties of aloe	1
6	Bryant (1966)	KwaZulu-Natal	Zulu medicine and medicine-men	9
7	Coopoosamy and Naidoo (2012)	KwaZulu-Natal	An ethnobotanical study of medicinal plants used by traditional healers in Durban	6
8	Corrigan et al. (2011)	KwaZulu-Natal	Ethnobotanical plant uses in the KwaNibela Peninsula of southern Maputaland, St Lucia	9
9	Cumes et al. (2009)	No restriction	Healing trees and plants of the Lowveld	1
10	De Beer and Van Wyk (2011)	Northern Cape	An ethnobotanical survey of the Agter-Hantam	38
11	Forbes (1986)	Western Cape	Traveller’s account of local floras	1
12	Gebashe et al. (2019)	KwaZulu-Natal	Ethnobotanical survey for medicinal grasses in KwaZulu-Natal Province	1
13	Gerstner (1941)	KwaZulu-Natal	A preliminary checklist of Zulu names of plants	9
14	Hulme (1954)	KwaZulu-Natal	Inventory of wild flowers of Natal	5
15	Hulley and Van Wyk (2019)	Western Cape	Quantitative medicinal ethnobotany of Kannaland (western Little Karoo)	107
16	Hutchings et al. (1996)	KwaZulu-Natal	Ethnobotanical book on Zulu Medicinal plants	57
17	Mabogo (1990)	Limpopo	An inventory of plants used by the Vhavenda	11
18	Mahwasane et al. (2013)	Limpopo	Ethnobotanical survey on plants for treating various human ailments by the traditional healers of the Lwamondo area	6
19	Maroyi (2017)	Eastern Cape	Useful plant species recorded in the Eastern Cape Province	18
20	Mbanjwa (2020)	KwaZulu-Natal	A quantitative ethnobotanical survey of the Ixopo area	47
21	Mhlongo and Van Wyk (2019)	KwaZulu-Natal	An ethnobotanical survey at Amandawe	123
22	Mintsa Mi Nzue (2009)	Western Cape	Use and conservation status of medicinal plants in the Cape Peninsula	27
23	Mogale et al. (2019)	Limpopo	Ethnobotany of Sekhukhuneland and the plants used by rural Bapedi people	14
24	Mokganya and Tshisikhawe (2019)	Limpopo	Medicinal value of wild vegetables consumed by local people of Vhembe District Municipality	4
25	Mongalo and Makhafola (2018)	Limpopo	Ethnobotanical knowledge of the lay people of Blouberg area (Pedi tribe), Limpopo Province	17
26	Nortje and Van Wyk (2015)	Northern Cape	An ethnobotanical survey of medicinal plants of the Kamiesberg area (an important Khoisan and Nama cultural center)	49
27	Palmer and Pitman (1961)	No restriction	An inventory of trees of South Africa	5
28	Philander (2011)	Western Cape	Ethnobotany of Western Cape Rasta bush medicine	49
29	Polori et al. (2018)	Free State	Ethnomedical botany and some biological activities of *Ipomoea oblongata*	1
30	Pooley (1993)	KwaZulu-Natal	Inventory on the trees of Natal, Zululand, and Transkei	6
31	Pujol (1990)	No restriction	An account of the herbalist medicine	17
32	Shai et al. (2020)	Mpumalanga	An ethnobotanical survey for locally sourced fruits among the Mapulana people	4
33	Thring and Weitz (2006)	Western Cape	Medicinal plant use in the Bredasdorp/Elim region of the southern Overberg	15
34	Tshikalange et al. (2016)	Mpumalanga	An ethnobotanical study of medicinal plants used in villages under Jongilanga tribal council	13
35	Van Wyk and Gericke (2000)	No restriction	Inventory on useful plants of Southern Africa	8
36	Van Wyk et al. (1997)	No restriction	Inventory on medicinal plants of South Africa	3
37	Van Wyk et al. (2008)	Eastern Cape and Western Cape	Ethnobotanical survey of medicinal plants in the southeastern Karoo	36
38	Watt and Breyer-Brandwijk (1962)	No restriction	Inventory on the medicinal and poisonous plants of Southern and Eastern Africa	69

**TABLE 2:** Ethnobotanical information of plants used for mitigating pain and inflammatory-related conditions in South Africa. Botanical names were verified using PlantZAfrica (pza.sanbi.org) and South African National Biodiversity Institute website (http://newposa.sanbi.org/sanbi/Explore) as well as the World Flora Online (http://www.worldfloraonline.org/). The listed 87 plants had ≥3 mentions and the full list of 495 plants recorded in the current study is presented in Supplementary Table S1. *Common name: A, Afrikaans; E, English; K, Khoi; KS, Khoisan (Khoe-San), SS, Southern Sotho; SL, Sepulana; NS, Northern Sotho; TW, Twana; X, Xhosa; V, Vhenda; Z, Zulu. #Part used; ns, not specified; Nm, number of mentions/citations.

Botanical name	*Common name	Family	Life-form	#Part used	Application(s)	Reference	Nm
*Acokanthera oppositifolia* (Lam.) Codd. Synonyms: *Acokanthera venenata* and *Carissa acokanthera*	Bushman’s arrow poison, Hottentot’s poison bush, kaffir poison bush, poison bush/tree (E), iNxinene (X), ubuhlungu benyoka, umkhwangu, and Inhlungunyembe (Z)	Apocynaceae	Shrub	Leaves and roots	For painful feet, rheumatism, toothache, abnormal menstrual period, and swellings. Analgesic (headache, general pain, and sharp internal body pains). Powder made from the dry roots is used as a stuff for headache	Hutchings et al. (1996); Watt and Breyer-Brandwijk (1962); Mhlongo and Van Wyk (2019); Bhat and Jacobs (1995)	4
*Agathosma betulina* (Berg.) Pillans. Synonyms: *Diosma betulina* Thunb. and *Bucco betulina* Schult.	Long-leaf buchu (E), langblaar boegoe (A), buchu (K), and ibuchu (X)	Rutaceae	Shrub	Leaves	For relieving the symptoms of rheumatism and easing backache. Dried leaves are used to treat stomachache. Infusions used for arthritis, inflammation, and backache. Applied externally for sprains and (arthritic) pains. Dried leaves are placed on a cloth and sprinkle with brandy/vinegar and wrapped around the affected area to relieve the pain	Watt and Breyer-Brandwijk (1962); De Beer and Van Wyk (2011); Hulley and Van Wyk (2019); Philander (2011); Thring and Weitz (2006); Mintsa Mi Nzue (2009)	6
*Aloe arborescens* Mill. Synonyms: *Aloe arborea* Medik. and *Catevala arborescens* (Mill.) Medik.	Krantz aloe (E), kransaalwyn (A), ikalene (X), Inhlabane, and Inkalane (Z)	Xanthorrhoeaceae	Shrub	Leaves, ns	Musculoskeletal inflammation. A small portion of leaves are mixed with chicken feed as an anti-inflammatory herb. Two-three spoons of leaf gel are taken orally to treat stomachache. For relieving menstrual pains and poultice for painful feet	Mhlongo and Van Wyk (2019); Bhat (2014); Mbanjwa (2020)	3
*Aloe ferox* Mill. Synonyms: *Aloe galpinii* Baker and *Aloe muricata* Haw.	Bitter aloe, red aloe, century tree (E), bitteraalwyn, bergaalwyn (A), iKhala, umHlaba, uNomaweni (X), and iNhlaba (Z)	Xanthorrhoeaceae	Shrub	Leaves	Leaves are boiled in water and taken orally for arthritis. Leaf gel is used for stomachache. Leaf infusion is used for back pain. Leaf decoctions (half a cup) are taken orally for stomachache. Leaf infusion is taken orally as a gargle for toothache. For relieving headache	Watt and Breyer-Brandwijk (1962); Bruce (1975); Maroyi (2017); De Beer and Van Wyk (2011); Bhat and Jacobs (1995); Bhat (2014); Hulley and Van Wyk (2019); Mbanjwa (2020)	8
*Anemone vesicatoria* (L.f.) Prantl. Synonyms: *Knowltonia vesicatoria* (L.f.) Sims and *Christophoriana vesicatoria* (L.f.) Kuntze	Blisterleaf (E), brandblaar, katjiedrieblaar, and tandpynblaar (A)	Ranunculaceae	Herb	Leaves	Fresh leaf infusions are used for rheumatism. Used to treat toothache and headache	Forbes (1986); Hutchings et al. (1996); Hulley and Van Wyk (2019)	3
*Artemisia afra* Jacq. ex Willd. Synonyms: *Absinthium ponticum* (L.) Garsault and *Absinthium tenuifolium* Gaterau	Wild wormwood, African wormwood (E), Wildeals (KS), and Mhlonyane (Z)	Asteraceae	Shrub	Leaves and stem	Leaves used as a compress with cooking oil to alleviate pain (inflammation). For treating backache and stomach pain. Infusion used for headache and earache. Leaves placed in ear as a bud for toothache. Infusion made from a handful of the leaves can be taken daily to treat headache. Leaves are made into a poultice for inflammation and rheumatism	Nortje and V3an Wyk (2015); De Beer and Van Wyk (2011); Coopoosamy and Naidoo (2012); Hulley and Van Wyk (2019); Thring and Weitz (2006); Mintsa Mi Nzue (2009)	6
*Asclepias crispa* P.J.Bergius. Synonyms: *Asclepias sabulosa* Schltr. and *Asclepias sinuosa* Burm.f.	Bitter Root (E), bitterhout (A), and Witvergeet (KS)	Apocynaceae	Herb	Roots	The root used with clove as snuff for headache, root chewed, and placed in a tooth for toothache. Root decoctions used for toothache and stomachache. Root infusion is used for the treatment of rheumatism	Nortje and Van Wyk (2015); Van Wyk et al. (2008); Hulley and Van Wyk (2019)	3
*Athrixia phylicoides* DC.	Bushman’s tea (E), Boesmanstee (A), icholocholo, itshelo, Inkalane, Ishanelo, and umtshanela (Z)	Asteraceae	Shrub	Leaves	Leaves are used with roots of *Athrixia elata* in decoctions for bathing sore feet. Musculoskeletal (inflammation). For relieving headache	Watt and Breyer-Brandwijk (1962); Mhlongo and Van Wyk (2019); Mbanjwa (2020)	3
*Baccharoides adoensis* (Sch.Bip. ex Walp.) H.Rob. Synonyms: *Ascaricida adoensis* Steetz and *Vernonia polymorpha* var. *polymorpha*	innyathelo, inyathelo, and uhlonyane (Z)	Asteraceae	Herb	Leaves, stem, and roots	Decoctions from leaves and stems are used for stomach cramps, nervous spasms of the stomach, and backbone pain. Root decoctions are taken for chest pain	Watt and Breyer-Brandwijk (1962); Pujol (1990); Hutchings et al. (1996)	3
*Ballota africana* (L.) Benth. Synonyms*: Marrubium africanum* L. and *Stachys africana* (L.) Kuntze	Cape horehound, Cat Herb, Catmint (E), Kattekrui, Kattekruie, kattekruid (A), and Kattekruid (KS)	Lamiaceae	Herb	Leaves	Leaves are used for the treatment of arthritis. As compress on sick children`s feet to get rid of the pains, on head for headache, and on cheek for toothache. Leaf infusion is given for stomach pain and headache. For washing aching legs. Applied as an ointment to pain and inflammation as well as backache. Leaf infusion used externally for headache and rheumatism	Watt and Breyer-Brandwijk (1962); Nortje and Van Wyk (2015); Van Wyk et al. (2008); De Beer and Van Wyk (2011); Hulley and Van Wyk (2019)	5
*Berchemia zeyheri* (Sond.) Grubov. Synonyms: *Phyllogeiton zeyheri* and *Rhamnus zeyheri*	Ivory wood, red ebony (E), Dinee (SL), umgologolo, umncaka, umneyi, and umnini (Z)	Rhamnaceae	Tree	Bark and roots	Bark infusions are administered as enemas for pains in the back and for rectal ulceration in children. Roots are used as a remedy to relieve headache	Mabogo (1990); Watt and Breyer-Brandwijk (1962); Shai et al. (2020)	3
*Bidens pilosa* L. Synonyms: *Bidens alausensis* Kunth and *Bidens cannabina* Lam.	Black jack, Spanish needles (E), knapsekerel, wewenaars (A), inongwe (X), amalenjane, and uqadolo (Z)	Asteraceae	Herb	Stem, seeds, and leaves	Young shoots are chewed for rheumatism. Burnt seed is rubbed into scarifications on the sides of the body for the relief of pain. Leaf decoction (1/4^th^ a cup) is taken twice daily to treat arthritis. Squeezed liquid from leaves is used as ear drops to relieve earache. For relieving menstrual pains	Watt and Breyer-Brandwijk (1962); Hutchings et al. (1996); Bhat (2014); Mokganya and Tshisikhawe (2019); Mbanjwa (2020)	5
*Boophone disticha* (L. f.) Herb. Synonyms: Amaryllis disticha L.f. and *Boophone longipedicellata* Pax	Cape poison bulb, sore-eye flower (E), gitbol, gifui, kopseerblom (A), incotho, incwadi, Ingcotho, and Umayime (Z)	Amaryllidaceae	Herb	Bulbs	Bulb decoctions are administered by mouth or as enemas to adults for headaches, sharp chest pains, and persistent bladder pains. For patients suffering from *inkwatshu,* a condition characterized by the development of cramp-like pains in the calf muscles associated with a feeling of tightness in the fingers and toes. Moistened bulb scales used for rheumatic pain. Analgesic (lower back aches). Bulb leaves used as a compress for pain and inflammation	Watt and Breyer-Brandwijk (1962); Hutchings et al. (1996); Mhlongo and Van Wyk (2019); Coopoosamy and Naidoo (2012); Hulley and Van Wyk (2019)	5
*Bridelia micrantha* (Hochst.) Baill. Synonyms: *Bridelia stenocarpa* Müll.Arg. and *Candelabria micrantha* Hochst.	Coastal goldenleaf (E), bruinstinkhout (A), Ditsere (SL), munzere (V) umhlamagwababa, and umshonge (Z)	Phyllanthaceae	Tree	Roots, bark, and leaves	Roots are used for severe epigastric pain and rubbed into the scalp for headache. Bark used for toothache and leaves for painful eyes and headache. Bark decoction is used to rinse the oral cavity to relieve toothache	Hutchings et al. (1996); Mabogo (1990); Shai et al. (2020), Arnold and Gulumian (1984)	4
*Bulbine latifolia* (L.f.) Spreng. Synonyms: *Bulbine brunsvigiaefolia* and *Bulbine natalensis*	Broad-leaved bulbine, red carrot (E), rooiwortel, geelkopieva (A), incelwane (X), and ibhucu (Z)	Xanthorrhoeaceae	Herb	Tubers and roots	Xhosa and Dutch settlers use tubers for rheumatism. Root infusions or decoctions are used for rheumatism. Roots are used for treating arthritis	Hutchings et al. (1996); Van Wyk and Gericke (2000); Philander (2011)	3
*Cannabis sativa* L. Synonyms: *Cannabis chinensis* Delile and *Cannabis sativa* var. *indica* (Lam.) Wehmer	Marijuana (E), dagga (A), Umya, Matakwane, intsangu (X), Matekwane/Patse (NS), and Nsangu (Z)	Cannabaceae	Herb	Whole plant and leaves	Whole plant is used to treat excessive headache. Leaf decoction is taken to relieve chronic pain. Used as painkillers and for toothache	Mongalo and Makhafola (2018); Bhat (2013); Mbanjwa (2020)	3
*Capparis tomentosa* Lam. Synonyms: *Capparis alexandrae* Chiov. and *Capparis subtomentosa* De Wild.	Woolly caper bush (E), Wollerige kapperbos (A), inkunzi-ebomvu, iqwaningi, and umqoqolo (Z)	Capparaceae	Tree	Roots	The roots boiled in water half a cupful of the infusion are taken three times a day. Powdered roots are rubbed on swollen ankles. Roots are burnt and the smoke is inhaled to relieve headache	Hutchings et al. (1996); Pujol (1990); Watt and Breyer-Brandwijk (1962); Arnold and Gulumian (1984)	4
*Carpobrotus edulis* (L.) N.E.Br. Synonyms: *Abryanthemum edule* (L.) Rothm*.* and *Mesembryanthemum edule* L.	Sour fig, Cape fig, Hottentots fig (E), ghaukum, ghoenavy, Hottentotsvy, Kaapsevy, perdevy, rankvy, suurvy, vyerank (A), ikhambi-lamabulawo, and umgongozi (Z)	Aizoaceae	Creeper	Leaves	To treat painful lungs. Leaf juice gargled for sore throat, teething problems, and earache as well as stomachache	Van Wyk et al. (2008); Hulley and Van Wyk (2019); Philander (2011); Mogale et al. (2019)	4
*Catharanthus roseus* (L.) G.Don. Synonyms: *Lochnera rosea, Pervinca rosea* (L.) Gaterau and *Vinca rosea* var. *alba* (G. Don) Sweet	Periwinkle, vinca (E), Imbali yamathuna, Imbali yesibaya, Isona, Ubani bezwe, and Umangashi (Z)	Apocynaceae	Herb	Leaves, roots, and milky sap	Leaves are used for rheumatism. Milky sap is used for insect bites. Roots are used for toothache. Analgesic (headache; toothache)	Watt and Breyer-Brandwijk (1962); Hutchings et al. (1996); Mhlongo and Van Wyk (2019); Mogale et al. (2019)	4
*Centella asiatica* (L.) Urb. Synonyms: *Centella hirtella* Nannf. and *Hydrocotyle biflora* P. Vell.	Marsh Pennywort (E), kleinkattekruid, varkoortjies (A), Umangobozane, and Isgoba (Z)	Apiaceae	Herb	Whole plant and leaves	Fresh plant decoction is taken orally for rheumatoid arthritis. Analgesic (sharp internal body pains). Fresh leaves used as ear plugs to relieve ear pain in children	Van Wyk and Gericke (2000); Mhlongo and Van Wyk (2019); Van Wyk et al. (2008)	3
*Chironia baccifera* L. Synonyms: *Chironia parviflora* Salisb. and *Chironia baccifera* var. *elongata* E.Mey.	Christmas berry (E), aambeibossie, bitterbossie (A), and Bitterbos (KS)	Gentianaceae	Shrub	Leaves and whole plant	Used for backache, rheumatism, arthritis, and woman ailments (menstrual pains). Infusions used for stomach ailments, pain and inflammation, backache, and headache	Nortje and Van Wyk (2015); Hulley and Van Wyk (2019); Mintsa Mi Nzue (2009)	3
*Cissampelos capensis* L.f. Synonyms: *Antizoma capensis* (L.f.) Diels and *Phyllanthus cinereoviridis* Pax	Davidjieswortel, Dawidjieswortel, and Fynblaarklimop (A)	Menispermaceae	Shrub	Roots and leaves	Roots are chewed for severe stomach pain. Root infusions as a remedy for toothache and headache. Fresh leaf infusions and decoctions are used for treating pain, backache, and stomach ailments	Van Wyk et al. (2008); Hulley and Van Wyk (2019); Mintsa Mi Nzue (2009)	3
*Cliffortia odorata* L.f. Synonyms: *Cliffortia alnifolia* Rchb. and *Cliffortia odorata* var. *vera* Harv.	Wild vine (E), wildewingerd, and wildevyerank (A)	Rosaceae	Shrub	Leaves, roots, and stem, ns	Used for backache, pain, and inflammation as well as arthritis	Hulley and Van Wyk (2019); Philander (2011); Mintsa Mi Nzue (2009)	3
*Colocasia antiquorum* Schott. Synonyms: *Colocasia fontanesii* Schott and *Colocasia tonoimo* Nakai	Elephant’s ear (E), idumbe (lomfula), and idumbi (Z)	Araceae	Herb	Tubers, roots, and leaves	Crushed root decoctions are administered as enemas for stomach trouble. Tubers are used as poultices for rheumatism. Bruised leaves are applied directly to cuts from insect stings	Hutchings et al. (1996); Hulme (1954); Pujol (1990)	3
*Conyza scabrida* DC. Synonyms: *Nidorella ivifolia* (L.) J.C.Manning & Goldblatt, *Erigeron dentatus* Burm.f, and *Fimbrillaria baccharoides* Cass.	Oven Bush (E), Bakbos, oondbos, paddabos (A), and Vleiwilger (KS)	Asteraceae	Shrub	Leaves	Leaf decoction used for backache. Infusion with ballerja used for headache. Leaf infusions used for cramps after labor and pain as well as rheumatism. Used as a compress to relieve arthritis, pain and inflammation, headache, backache, and stomachache	Nortje and Van Wyk (2015); Van Wyk et al. (2008); De Beer and Van Wyk (2011); Hulley and Van Wyk (2019); Philander (2011); Thring and Weitz (2006)	6
*Cotyledon orbiculata* L. Synonyms: *Cotyledon ambigua* Salisb. and *Cotyledon tricuspidata* Haw.	Pig’s ears, cotyledon (E), plakkie, platjies, varkoorblare, varkoor, kouterie (A), imphewula (X), and ipewula (Z)	Crassulaceae	Herb	Leaves	Leaves are boiled and filtered, and a drop of decoction is used for earache. Leaves are heated and placed on a swollen body part to treat inflammation. Leaf juice treats earache and toothache. Used for earache	Bhat and Jacobs (1995); Hulley and Van Wyk (2019); Philander (2011)	3
*Crinum macowanii* Baker. Synonyms: *Crinum corradii* Chiov. ex Chiarugi and *Crinum pedicellatum* Pax	Boslelie (A), Cape Coast Lily, Common Vlei Crinum (E), Intelezi (X), Intelezi, Uguqu, and Umduze (Z)	Amaryllidaceae	Herb	Bulbs	Decoctions of the bulb taken orally for rheumatic fever. Analgesic (toothache); musculoskeletal inflammation). Used for headache	Hutchings et al. (1996); Van Wyk and Gericke (2000); Mhlongo and Van Wyk (2019); Mbanjwa (2020)	4
*Croton steenkampianus* Gerstner	Marsh Fever-berry (E), vleikoorsbessie (A), and uhubeshane omkhulu (Z)	Euphorbiaceae	Shrub	Leaves	Steam from fresh leaf decoctions is inhaled to relieve aches. A remedy against painful joints, back, and rheumatism	Pooley (1993); Watt and Breyer-Brandwijk (1962); Hutchings et al. (1996)	3
*Dalbergia armata* E.Mey.	Thorny-rope, flat-bean, Hluhluwe climber (E), doringtou (A), sehlokootswa (NS), uBobo (X), Umhluhluwe, and Umhluhlube (Z)	Fabaceae	Shrub	Roots and leaves, ns	Analgesic (sharp internal body pains). The roots are boiled in water and the water is gargled to relieve toothache. Leaf decoction is taken (half a cup thrice daily) for body pains	Mhlongo and Van Wyk (2019); Corrigan et al. (2011); Bhat and Jacobs (1995)	3
*Datura stramonium* L. Synonyms: *Datura bernhardii* and *Datura inermis*	Downy thorn apple, ditch weed, Jimson weed, stinkwort (E), gewone stinkblaar, malpitte (A), Stinkblaar (KS), umhlavuthwa (X), and iloqi (Z)	Solanaceae	Herb	Leaves	Leaves are smoked for the relief of headaches. Leaf infusions are used for treating rheumatism. Powdered leaves are applied to human bruises to alleviate inflammation. Leaves applied as a compress on pain (inflammation), backache, headache, and earache	Van Wyk et al. (1997); Watt and Breyer-Brandwijk (1962); Nortje and Van Wyk (2015); Hulley and Van Wyk (2019)	4
*Dicerothamnus rhinocerotis* (L.f.) Koek. Synonyms: *Elytropappus rhinocerotis* (L.f.) Less., *Stoebe rhinocerotis* L.f., and *Seriphium adpressum* DC.	Rhinoceros bush, rhenoster bush (E), renosterbos, rhenosterbos (A), and Renosterbos (KS)	Asteraceae	Shrub	Leaves and stem	Leaf decoction used for painful legs, as a wash for burning feet, and as a compress for backache. Used as a wash for rheumatism. Infusion of young stem is used for back pain. Leaves are chewed and juices swallowed for stomachache. Infusions used for stomachache, headache, and earache	Nortje and Van Wyk (2015); Van Wyk et al. (2008); Hulley and Van Wyk (2019); Thring and Weitz (2006)	4
*Dicoma capensis* Less. Synonyms: *Berkheya albida* DC. and *Tibestina lanuginosa* Maire	Fever bush (E), Karmedik, verpis, vyfpondbos, Melktou (A), and koorsbos(sie) (KS)	Asteraceae	Herb	Leaves	Leaf infusion with other plants used for rheumatism and backache. For treating rheumatism and stomach pain	Nortje and Van Wyk (2015); Van Wyk et al. (2008); De Beer and Van Wyk (2011)	3
*Dodonaea viscosa* Jacq var. *angustifolia* (L.f) Benth. Synonym: *Dodonaea arabica* Hochst. & Steud. and *Dodonaea angustifolia* L.f.	Sand olive (E), makkaree, Sandolien, ysterhouttoppe (A), and mutata-vhana (V)	Sapindaceae	Shrub	Leaves	Leaf decoctions are used against arthritis. Infusion of leafy tips is used for back pain. Powdered leaves used as a snuff for headache. Leaf infusions used for pain and inflammation, backache, and arthritis. The leaf tops (±3 teaspoons in 1 L boiling water) are made into an infusion and small amount taken 3 times daily to treat arthritis, inflammation, and rheumatism	Van Wyk et al. (1997); Watt and Breyer-Brandwijk (1962); Van Wyk et al. (2008); De Beer and Van Wyk (2011); Hulley and Van Wyk (2019); Philander (2011); Thring and Weitz (2006); Mintsa Mi Nzue (2009)	8
*Dovyalis caffra (*Hook.f. & Harv.) Sim. Synonyms: *Aberia caffra* and *Dovyalis caffra* (Hook. f. & Harv.) Warb.	Kei-apple, Dingaan’s apricot, wild apricot (E), Kei-appel, appelkoosdoring (A), mutunu (V), and Umqokolo (Z)	Salicaceae	Shrub	Root, bark, and thorn	Roots and thorns are used for treating chest pain. Decoction of the bark and root is a remedy for rheumatism. Analgesic (sharp internal body pains). Thorn decoction is drunk for pain in chest (heart side)	Bryant (1966); Cumes et al. (2009); Watt and Breyer-Brandwijk (1962); Mhlongo and Van Wyk (2019); Arnold and Gulumian (1984)	5
*Drimia elata* Jacq. Synonyms: *Drimia alta* R.A.Dyer and *Drimia robusta* Baker	Satin Squill (E), brandui, maerman (A), indongana-zibomvana, and isiklenama (Z)	Asparagaceae	Herb	Bulbs	Bulb scales are rubbed on the chest for stabbing pains. Poultice of the bulb is used against pain and inflammation. Topical arthritis remedy	Hutchings et al. (1996); Hulley and Van Wyk (2019); Philander (2011)	3
*Ekebergia capensis* Sparrm. Synonyms: *Ekebergia buchananii* Harms and *Trichilia ekebergia* E. Mey. ex Sond.	Cape ash, dog plum (E), essenhout, rooiess(en)hout (A), nyamaru (TW), mmidibidi (NS), Mutovuma (V), umnyamathi, umthoma, usimanaye, and uvungu (Z)	Meliaceae	Tree	Roots, bark, and leaves	Roots are used for headaches. Leaves and bark are used for headache. Bark is macerated and used as an enema to relieve backache for 2 days	Watt and Breyer-Brandwijk (1962); Mabogo (1990); Arnold and Gulumian (1984)	3
*Erythrina lysistemon* Hutch. Synonym: *Erythrina caffra* var. *mossambicensis*	Common coral tree, lucky bean tree (E), gewone koraalboom, kanniedood (A), umsintsi (X), muvhale (V), mophete (TW), and umsinsi (Z)	Fabaceae	Tree	Bark	Bark is used to treat arthritis and toothache. Bark is used as a poultice for swellings and abscesses. The bark s using toothache	Pujol (1990); Mabogo (1990); Van Wyk et al. (1997); Mbanjwa (2020)	4
*Eucomis comosa* (Houtt.) Wehrh. var. *comosa*. Synonyms: *Eucomis pallidiflora* Bak and *Eucomis punctata* L'Herit.	Pineapple flower (E), krulkoppie, Pynappellelie (A), Ubuhlungu-becanti (X), and Ubuhlungu-becanti (Z)	Asparagaceae	Herb	Roots and bulbs	Medicine made from the root is administered as an antirheumatic in doses of one spoonful. Bulb decoctions are used for rheumatism	Gerstner (1941); Hutchings et al. (1996); Watt and Breyer-Brandwijk (1962)	3
*Foeniculum vulgare* Mill. Synonyms: *Anethum foeniculum* L. and *Foeniculum officinale* ALL.	Fennel, vinkel (E), i(li)beka, imbozisa, and imboziso(-eluhlaza) (Z)	Apiaceae	Herb	Leaves	Leaf decoctions are taken three times a day or taken as enemas for pain in the side. Used for cramps and stomach ache. Analgesic (toothache); musculoskeletal inflammation. Infusion of the leaves is used for stomachache and arthritis	Hutchings et al. (1996); Watt and Breyer-Brandwijk (1962); Mhlongo and Van Wyk (2019); Hulley and Van Wyk (2019); Thring and Weitz (2006)	5
*Galenia africana* L. Synonyms: *Galenia linearis* Thunb. and *Galenia tenuifolia* Salisb.	Geelbos, perdebos, kraalbos (A), and Kraalbos (KS)	Aizoaceae	Shrub	Leaves and twigs	Twig/leaf placed in tooth for toothache. Bathe in a weak infusion to relieve rheumatism. Leaf infusion used to treat leg pain. Used as wash or ointment and as a rinse for toothache, rheumatism, pain, and inflammation	Nortje and Van Wyk (2015); Van Wyk et al. (2008); De Beer and Van Wyk (2011); Hulley and Van Wyk (2019); Philander (2011)	5
*Galium tomentosum* Thunb. Synonyms: *Galium asperum var. villosum* Eckl. & Zeyh. and *Galium glabrum* Thunb.	Old Man’s Beard (E), Rooivergeet, Kleefgras (A), and Jantjiegoub (KS)	Rubiaceae	Herb	Roots	Roots are powdered and used as a snuff for headache. As a remedy for inflammation	Nortje and Van Wyk (2015); Hulley and Van Wyk (2019); Philander (2011)	3
*Gnidia kraussiana* Meisn. Synonyms: *Gnidia hoepjiwriana*, *Lasiosiphon hoepfnerianus*, and *Lasiosiphon kraussianus* (Meisn.) Burtt Davy	Yellow heads (E), gifbossie (A), umarhedeni (X), isidikili, imfuzane, and umsilawengwe (Z)	Thymelaeaceae	Herb	Roots	Strong enemas made from root extracts are taken for stomach complaints. Roots in milk decoctions for backache and stomach sores. Root decoctions or infusions are taken for chest complaints. For lower back pain	Hulme (1954); Watt and Breyer-Brandwijk (1962); Hutchings et al. (1996); Mbanjwa (2020)	4
*Gomphocarpus fruticosus* (L.) W.T.Aiton. Synonyms: *Asclepias fruticosa* and *Gomphocarpus crinitus* G.Bertol.	Milkweed, narrow-leaved cotton bush, wild cotton (E), blaasoppies (A), Gewone (KS), ulusinga Iwesalukazi, and umsinga-lwesalukazi (Z)	Apocynaceae	Shrub	Leaves and roots	Leaf infusions are administered for stomach pain in children. Roots are used for general body pain and stomach ache. Root used as a snuff for headache and dry leaves used as a snuff for headache. Leaf decoction is taken orally as a headache treatment	Gerstner (1941); Hulme (1954); Pujol (1990); Watt and Breyer-Brandwijk (1962); Nortje and Van Wyk (2015); De Beer and Van Wyk (2011); Mogale et al. (2019)	7
*Gunnera perpensa* L. Synonyms: *Gunnera calthifolia* and *Perpensum blitispermum*	River pumpkin, wild Rhubarb (E), Iphuzi, Uxobo (X), Rivierpampoen (A), qobo (SS), Izibu, Ugobho, Uklenya, and Uxobo (Z)	Gunneraceae	Herb	Roots and rhizomes	Together with other plants such as *Alepidea amatymbica* and *Crinum* sp., the root decoctions are taken for pain in rheumatic fever and stomachache. Musculoskeletal (inflammation). Inflammation and menstrual pain. Root decoction is used to treat menstrual pain. Leaves used as a compress on pain and inflammation especially rheumatism and backache. Used as a compress for headache. Used for relieving menstrual pains and afterbirth pain	Bryant (1966); Mhlongo and Van Wyk (2019); Maroyi (2017); Bhat (2014); Hulley and Van Wyk (2019); Mbanjwa (2020)	6
*Helichrysum cymosum* (L.) D.Don. Synonyms: *Gnaphalium cernuum* Thunb. and *Gnaphalium tricostatum* Sieber ex DC.	Gold carpet (E), goue tapyt (A), imPepho, and imPepha (X)	Asteraceae	Herb	Leaves	Fresh leaves are boiled in water and the vapor used as a vapor bath for treating headache. For treating toothache	Bhat and Jacobs (1995); Bhat (2013); Mbanjwa (2020)	3
*Helichrysum odoratissimum* (L.) Sweet. Synonyms: *Gnaphalium strigosum* Thunb. and *Helichrysum rosmarinum* Mattf.	Most fragrant helichrysum (E), kooigoed, kruie (A), iphepho (X), and Hotnotskooigoed (KS)	Asteraceae	Herb	Whole plant, ns	Infusion used for backache. Whole plant is used for headache. Used for treating pain and inflammation, backache, toothache, menstrual pains, and cramp	Nortje and Van Wyk (2015); Maroyi (2017); Hulley and Van Wyk (2019); Mbanjwa (2020)	4
*Hypoxis hemerocallidea* Fisch., C.A.Mey. & Avé-Lall. Synonyms: *Hypoxis rooperi* S. Moore and *Hypoxis patula* Nel	African potato, Star flower, yellow star (E), sterblom, geelsterretjie, gifbol (A), moli kharatsa, lotsane (SS), tshuka (TW), inongwe, ixhalanxa, ikhubalo lezithunzela (X), inkomfe, and inkomfe enkulu (Z)	Hypoxidaceae	Herb	Corms and rootstock	Infusions and decoctions of the plant are used for rheumatism. Juice from the rootstock is applied to burns. Corms are traditionally used for headaches. Analgesic (back pains, sharp internal body pains); musculoskeletal (arthritis)	Hutchings et al. (1996); Van Wyk and Gericke (2000); Mhlongo and Van Wyk (2019); Philander (2011); Mintsa Mi Nzue (2009)	5
*Leonotis leonurus* (L.) R.Br. Synonyms: *Hemisodon leonurus* (L.) Raf. and *Phlomis leonurus* L.	Wild dagga, lion’s ear, leonotis (E), wildedagga, duiwelstabak (A), imvovo, utywala-bengcungcu, umfincafincane, umunyamunya (X), umfincafincane, umcwili, and utshwala-bezinyoni (Z)	Lamiaceae	Shrub	Leaves and flowers	Leaf decoction is taken orally to treat headache. Used to treat stomach ailments, backache, pain, and inflammation. An infusion is made from a handful of leaves and flowers steeped in boiling water and left to draw in a glass bottle. About 25 ml is drunk morning and night for arthritis, backache, headache, and rheumatism	Bhat (2013); Hulley and Van Wyk (2019); Philander (2011); Thring and Weitz (2006)	4
*Melianthus comosus* Vahl. Synonym: *Diplerisma comosum* (Vahl) Planch.	Touch-me-not, honey flower (E), Kruidjie-roer-my-nie (A), and ibonya (Z)	Melianthaceae	Shrub	Leaves and whole plant	Plant decoctions are used to bathe rheumatic limbs and painful feet while leaf paste has also been used to reduce the swelling of bruises. Herb is applied topically for the inflamed leg. Boiled leaves are applied to painful knees. For treating rheumatism painful back and legs. Used as a wash for pain and inflammation, rheumatism, backache, wounds, and sores and as a rinse for toothache	Hutchings et al. (1996); Van Wyk et al. (2008); De Beer and Van Wyk (2011); Hulley and Van Wyk (2019)	4
*Mentha longifolia* (L.) Huds. Synonyms: *Mentha aepycaulos* Candargy and *Mentha brassoensis* (Topitz) Trautm.	Wild mint (E), ballerja, balderjan, baldrian,t’kamma (A), Ballerja (KS), inixina, inzinziniba (X), and ufuthana lomhlanga (Z)	Lamiaceae	Herb	Leaves	Leaves used as a compress on pains and sores. Ointment used for painful legs. Prepared as an infusion for treating headache and arthritis. Warm leaves used as a compress to treat headache and stomach pains, used for washing aching legs. Used to treat toothache, headache, earache, pain, and inflammation	Nortje and Van Wyk (2015); Van Wyk et al. (2008); De Beer and Van Wyk (2011); Hulley and Van Wyk (2019); Thring and Weitz (2006)	5
*Nicotiana glauca* Graham. Synonyms: *Nicotiana glauca* f. *lateritia* Lillo and *Nicotiana glauca* var. angustifolia Comes	Tree tobacco (E), wilde twak, jantwak(boom), jan twak (A), Jantwaks (KS), and Icubamfene (X)	Solanaceae	Shrub	Leaves	Warmed leaves used as a compress on pains. Leaves used as a plug or as a wash for earache. Used for headache. Fresh leaves are applied to the head as a poultice to draw out the pain. Dried leaves are used as fumitory to get rid of headache. As a compress on the head for headache, earache, pain, and inflammation	Nortje and Van Wyk (2015); Maroyi (2017); Van Wyk et al. (2008); Bhat (2014); Hulley and Van Wyk (2019)	5
*Peltophorum africanum* Sond. Synonym: *Brasilettia africana*	Weeping wattle, Natal wattle (E), huilboom, kiaatboom (A), Mosêhla, Mosese (NS), Mosêtlha (TW), umsehle, and Isikhabamkhombe (Z)	Fabaceae	Tree	Roots and bark	Roots and bark are used for backache. Bark is also used for abdominal pain while leaves are used for toothache and abdominal pain. Root decoction used for treating body pain	Pooley (1993); Mabogo (1990); Watt and Breyer-Brandwijk (1962); Mogale et al. (2019); Tshikalange et al. (2016)	5
*Pentanisia prunelloides* subsp. *latifolia* (Hochst.) Verdc. Synonyms: *Pentanisia variabilis* Harv. var. *latifolia* and *Declieuxia latifolia* Hochst.	Wild verbena, broad-leaved Pentanisia (E), sooibrandbossie (A), isigcikamlilo (X), Icishamlilo, and Icishamlilo elikhulu (Z)	Rubiaceae	Herb	Leaves and roots	Decoctions are sprinkled on painful parts for treating rheumatism. Pounded roots are applied to burns and used in poultices for inflammation and swollen joints. Leaf poultices or hot root decoctions are applied to painful swellings, rheumatic parts, sprains, and sores. Analgesic (general body pains). Root decoction is used for treating rheumatism	Bryant (1966); Gerstner (1941); Hulme (1954); Watt and Breyer-Brandwijk (1962); Mhlongo and Van Wyk (2019); Bhat (2014); Mintsa Mi Nzue (2009); Mbanjwa (2020)	8
*Pentzia incana* (Thunb.) Kuntze. Synonyms: *Chrysanthemum incanum* Thunb. and *Pentzia virgata* Less.	Anchor Karoo, Common Karro (E), Ankerkaroo, Gansie, Alsbossie, Rooikarobos (A), and Mohantsoana (SS)	Asteraceae	Shrub	Leaves and twigs, ns	For stomachache. Leaves are chewed to treat stomach cramps and to treat general pain. Twigs are chewed to extract juices for treating stomachache, backache, pain, and inflammation	Van Wyk et al. (2008); De Beer and Van Wyk (2011); Hulley and Van Wyk (2019)	3
*Pittosporum viridiflorum* Sims. Synonyms: *Pittosporum abyssinicum*, *Pittosporum antunesii*, *Pittosporum commutatum*, and *Pittosporum floribundum*	Cheesewood (E), bosbeukenhout, Kasuur (A), Kgalagangwe (NS), Mosetlela (SS), Mutanzwakhamelo (V), umkhwenkwe (X), umfusamvu, umvusamu umkhwenkhwe, and umkwenkwe (Z)	Pittosporaceae	Shrub	Bark and roots	Bark decoctions are also taken for pains in the back as emetics or enemas for stomach troubles particularly those to ease pain. Root infusions are taken for chest pains. Taken for abdominal pain	Watt and Breyer-Brandwijk (1962); Maroyi (2017); Van Wyk et al. (2008)	3
*Platycarpha glomerata* (Thunb.) Less. Synonyms: *Cynara glomerata* Thunb. and *Stobaea glomerata* (Thunb.) Spreng.	Imbozisa, Imbozisa encane, Isiphahluka, Ubani, Ubani olukhulu, Ukhula, Umabopha,Umbola, and Umkhwibi ompofu (Z)	Asteraceae	Herb	Roots, ns	Internal side pain in children, chest pain, and musculoskeletal (inflammation). Yellow sap is used for cleaning ear or earache	Mhlongo and Van Wyk (2019); Philander (2011); Mbanjwa (2020)	3
*Plumbago auriculata* Lam. Synonyms: *Plumbagidium auriculatum* (Lam.) Spach and *Plumbago capensis*	Cape leadwort, plumbago (E), syselbos (A), Umabophe, umatshintshine, (X), umasheleshele, Ubani, and umaswelisweli (Z)	Plumbaginaceae	Shrub	Roots and leaves	Powdered roots or dried leaves are taken as snuff to relieve headaches. Analgesic (sharp internal body pains)	Gerstner (1941); Hutchings et al. (1996); Mhlongo and Van Wyk (2019)	3
*Ptaeroxylon obliquum* (Thunb.) Radlk. Synonyms: *Ptaeroxylon utile* Eckl. & Zeyh and *Rhus obliqua* Thunb*.*	Sneezewood, stinkhout (E), Nieshout (A), Mulari, Munari, Munukha-vhaloi (V), Umthathi, Umthote (X), Ithatha, Umthathi, Umzane, and Uthathi (Z)	Rutaceae	Tree	Bark	Bark infusions are used against rheumatism and arthritis. Powdered bark is used to relieve headaches	Pujol (1990); Watt and Breyer-Brandwijk (1962); Mhlongo and Van Wyk (2019)	3
*Rapanea melanophloeos* (L.) Mez. Synonyms: *Heeria melanophloeos* (L.) Meisn. and *Myrsine melanophloeos*	Cape beech tree (E), rooiboekenhout (A), Isiqwane Sehlathi (X), Mogônô (NS), Tshikonwa (V), Isihluthi-wentaba, and ikhubalwane (Z)	Primulaceae	Tree	Bark	Bark is used for stomach and muscular pain. Ground bark decoctions are taken for stomach ache	Gerstner (1941); Hutchings et al. (1996); Pujol (1990)	3
*Ricinus communis* L. Synonyms: *Ricinus africanus* Mill. and *Ricinus communis* var. *communis*	Castor bean (E), kasterolie (boom) (A), Olieboom, kasterolie (KS), Mupfure (V), Umkakuva, umhlakuva (X), Uhlakuva, and Umhlakuva (Z)	Euphorbiaceae	Shrub	Leaves, seeds, and fruit	Analgesic (toothache); musculoskeletal (inflammation). Oil from ground seeds used as ointment and leaves as a compress on pains and rheumatism. Leaves used as a compress on the cheek for toothache. Treating stomachache. Leaves are heated and placed on painful knees/joints. Oil squeezed from the fruits into the ear to relieve earache. Fresh leaves are ground and mixed with water and given orally to treat stomachache. Compress leaves used for headache, pain, inflammation, and sprains. Warm leaves are wrapped around a child for stomachache	Mhlongo and Van Wyk (2019); Nortje and Van Wyk (2015); Maroyi (2017); De Beer and Van Wyk (2011); Arnold and Gulumian (1984); Bhat (2014); Hulley and Van Wyk (2019), Philander (2011); Thring and Weitz (2006); Mbanjwa (2020)	10
*Rubus pinnatus* Willd. Synonyms: *Rubus kingaensis* Engl. and *Rubus pinnatus* var. *defensus* Gust*.*	South African blackberry (bramble or Raspberry), Capebramble (E), braambos braamboswortel (A), Iqunube (X), Ijingijolo, and Mfongosi (Z)	Rosaceae	Shrub	Roots	Root decoctions are taken for various respiratory ailments including pain in the chest. Roots are used for toothache either as warm water gargles or ground and inserted directly into the cavity	Hutchings et al. (1996); Pujol (1990); Watt and Breyer-Brandwijk (1962)	3
*Rubus rigidus* Sm. Synonyms: *Dyctisperma rigidus* (Sm.) Raf. ex B.D.Jacks. and *Rubus inedulis* Rolfe	White bramble (E), braambossie, braambos (A), Umgcunube (X), ijingijolo, Amajikijolo, and Amabhimbi (Z)	Rosaceae	Shrub	Roots	Root decoctions are taken as gargles for toothache. Root decoctions for acute pain during illnesses. Analgesic (toothache and sharp internal body pains)	Hutchings et al. (1996); Watt and Breyer-Brandwijk (1962); Mhlongo and Van Wyk (2019); Mbanjwa (2020)	4
*Ruta graveolens* L. Synonym: *Ruta hortensis* Mill.	Rue, common rue, Herb of Grace, Garden Rue (E), wynruit, wynruik (A), and Wynruit (KS)	Rutaceae	Shrub	Leaves and stem	Leaf infusion used for menstruation pains and toothache. As compress for low back pain and other pains. Leaf infusion to relieve stomachache and treatment of headache. Used for inflammation and earache	Nortje and Van Wyk (2015); Van Wyk et al. (2008); De Beer and Van Wyk (2011); Hulley and Van Wyk (2019); Philander (2011); Thring and Weitz (2006); Mintsa Mi Nzue (2009)	7
*Salix mucronata* Thunb. Synonyms: *Salix capensis* var. *mucronata* Anders and *Salix safsaf* Forssk. ex Trautv.	Wild willow (E), Treurwilg Wildewilgerboom (A), Munengeledzi (V), and Wilgerboom (KS)	Salicaceae	Tree	Branch tips, leaves, and bark	Indicated as a remedy against rheumatism. Leaf infusions used for backache. Leaves are used to treat pain. Infusions of the bark are used to treat rheumatism, pain, and inflammation	Van Wyk and Gericke (2000); Nortje and Van Wyk (2015); De Beer and Van Wyk (2011); Hulley and Van Wyk (2019)	4
*Schinus molle* L. Synonyms: *Schinus angustifolia* Sessé & Moc. and *Schinus occidentalis* Sessé & Moc.	Peruvian pepper, pepper tree (E), peperboom (KS), and peperboom (A)	Anacardiaceae	Tree	Leaves	Leaves as a compress on painful legs, backache, headache, and knee. Warm compress of leaves is placed on cheek for toothache. Vapor of leaf decoction is inhaled for inflammation and rheumatism. Leaf decoction is used to gargle to cure toothache. Compress for headache, pain, and inflammation. Infusions for headache, pain, and inflammation	Nortje and Van Wyk (2015); Van Wyk et al. (2008); Bhat and Jacobs (1995); Bhat (2014); Hulley and Van Wyk (2019)	5
*Sclerocarya birrea* (A.Rich.) Hochst. subsp. *caffra* (Sond.) Kokwaro. Synonyms: *Sclerocarya birrea*, *Sclerocarya caffra*, and *Sclerocarya schweinfurthiana*	Cider tree, marula, maroola (E), maeroola, maroelaboom (A), Mufula (V), and umganu (Z)	Anacardiaceae	Tree	Bark	Bark decoctions are used for abdominal pain. The bark is used for headaches, toothache (rinsing oral cavity), and backache	Hutchings et al. (1996); Mabogo (1990); Watt and Breyer-Brandwijk (1962); Arnold and Gulumian (1984)	4
*Securidaca longepedunculata* Fresen. Synonyms: *Elsota longipedunculata* (Fresen.) Kuntze, and *Securidaca longipedunculata* var. *longipedunculata*	Violet tree (E), Langboslaagboom (A), and Mpesu (V)	Polygalaceae	Tree	Bark, roots, and root-kernel	Bark and roots are taken orally as infusions and decoctions for rheumatism. Root-kernel is used to treat headache. Root decoction is drunk thrice daily to relieve backache	Van Wyk and Gericke (2000); Watt and Breyer-Brandwijk (1962); Mongalo and Makhafola (2018); Arnold and Gulumian (1984)	4
*Solanum aculeastrum* Dunal. Synonym: *Solanum aculeastrum* var. *aculeastrum*	Apple of Sodom, bitter apple, devil’s apple (E), Bitterappeltjie, Bokappel (A), Morola (NS), Murulwa, Shulwa (V), Intuma, Intuma, Intuma enkulu, Intumayezibaya, Uthuma, and Untumane (Z)	Solanaceae	Shrub	Fruit and roots, ns	Ash from burnt fruit is rubbed into scarifications over painful parts for the relief of rheumatism pains. Fruits are applied topically for toothache and are also placed in the wound after tooth extraction. Fruit decoctions are used as enemas for pain in the lower back and legs while ash is used for pains from walking. Analgesic (toothache, general pains, and backaches); musculoskeletal (inflammation). Roots are used to treat stomachache	Watt and Breyer-Brandwijk (1962); Hutchings et al. (1996); Mhlongo and Van Wyk (2019); Mongalo and Makhafola (2018); Philander (2011); Mintsa Mi Nzue (2009); Mbanjwa (2020)	7
*Solanum panduriforme* E. Mey.	Morolana (NS), Mututulwa (V), and Intuma encane (Z)	Solanaceae	Herb	Roots and fruit, ns	Analgesic (toothache); musculoskeletal (inflammation). Roots are used to treat stomachache. Fruits are burnt and powder is applied externally on incision made on the forehead to relieve headache	Mhlongo and Van Wyk (2019); Mongalo and Makhafola (2018); Arnold and Gulumian (1984)	3
*Solanum tomentosum* L.	Slangappelbos (E), gifappel, Vuilsiekbossie, Doringappeltjie, !nuheis, bitterboelabos (A), and Tandpynbossie (KS)	Solanaceae	Shrub	Fruit and leaves	Fruit is used for toothache. Ground leaves are a treatment for backache and stomachache	Nortje and Van Wyk (2015); De Beer and Van Wyk (2011); Hulley and Van Wyk (2019)	3
*Stangeria eriopus* (Kunze) Baill. Synonyms: *Stangeria katzeri* Regel and *Stangeria paradoxa* T.Moore	Natal Grass Cycad, Cycad (E), obbejaankos (A), Umfingwani, Umncuma (X), Imfingo, and Umafinga (Z)	Zamiaceae	Shrub	Tubers and roots	Burnt powdered underground tubers are used for headaches. Tubers are used for pains in the bones. Analgesic (sharp internal body pains). Roots used to treat headache	Hutchings et al. (1996); Watt and Breyer-Brandwijk (1962); Mhlongo and Van Wyk (2019); Coopoosamy and Naidoo (2012)	4
*Strychnos henningsii* Gilg. Synonyms: *Strychnos albersii* Gilg & Busse and *Strychnos holstii* Gilg	Coffee bean Strychnos, Natal teak, coffee hard pear (E), harclepeer(hout), rooibitterbessie (A), umanana, umdunye, umnono, umqalothi, and umqaloti (Z)	Loganiaceae	Shrub	Roots and bark	Boiled roots are used for stomach complaints. Bark decoctions boiled with roots of *Turraea floribunda* Hochst are taken for the pains of rheumatic fever. Bark is used in the treatment of dysmenorrhoea. Analgesic (body pains)	Hutchings et al. (1996); Watt and Breyer-Brandwijk (1962): Mhlongo and Van Wyk (2019); Philander (2011)	4
*Sutherlandia frutescens* (L.) R.Br. = *Lessertia frutescens* (L.) Goldblatt & J.C.Manning subsp. *frutescens*. Synonym: *Colutea frutescens* L.	Turkey flower, balloon pea, cancer bush (E), Wildekeur (A), Kankerbossie (KS), Umnwele (X), Umnwele, and Unwele (Z)	Fabaceae	Herb	Leaves, fruit, seeds, stem, flowers, and whole plant	Different parts of the plant are used for treating backache and rheumatism. Leaf wash used for painful feet. Chewed in the mouth and placed on tooth to help with toothache. Infusion is used to treat backache and stomach ailments	Watt and Breyer-Brandwijk (1962); Nortje and Van Wyk (2015); De Beer and Van Wyk (2011); Hulley and Van Wyk (2019); Thring and Weitz (2006); Mintsa Mi Nzue (2009)	6
*Syzygium cordatum* Hochst.ex C.Krauss. Synonyms: *Syzygium cymiferum* (E.Mey.) C.Presl and *Jambosa cymifera* E.Mey.	Water Wood, water berry (E), waterbessie (A), Mutu (V), umswi, umjomi (X), and Umdoni (Z)	Myrtaceae	Tree	Bark, leaves, and roots	The Bemba use cold leaf infusions for various stomach ailments. Vhavenda use leaves for fever while bark and roots are used for headache. For treating inflammation. Roots are burnt and the ash is applied on incisions on forehead to relieve headache. Used for relieving menstrual pain	Hutchings et al. (1996); Watt and Breyer-Brandwijk (1962); Mabogo (1990); Maroyi (2017); Arnold and Gulumian (1984); Mbanjwa (2020)	6
*Trichilia dregeana* Sond. Synonym: *Trichilia strigulosa* Welw. ex C.DC.	Natal forest mahogany (E), Bosrooiessenhout (A), Mutshikili, Mutuhu, Muuhu (V), umathunzini, umkhuhla, Igxolo, and umkhuhlu (Z)	Meliaceae	Tree	Bark	Bark decoctions are administered as enemas for backache associated with kidney problems. Unspecified parts are used for stomach complaints and backache. Analgesic (backaches, lower backaches, and toothache)	Hutchings et al. (1996); Watt and Breyer-Brandwijk (1962); Mhlongo and Van Wyk (2019)	3
*Tulbaghia violacea* Harv. Synonyms: *Omentaria alliacea* (L.f.) Kuntze and *Tulbaghia brachystemma* Kunth	Wild garlic (E), wildeknofflok, wildeknoffel, bergknoffe (A), and isihaqa (Z)	Amaryllidaceae	Herb	Tubers/bulbs, roots, and leaves	Pounded tuber decoctions are administered as enemas for stomach ailments. Leaves are rubbed on the head for sinus headache. Administered in enemas for rheumatism. Clove pieces are placed in castor oil to make eardrops. Used for stomach ailments	Watt and Breyer-Brandwijk (1962); Hutchings et al. (1996); Hulley and Van Wyk (2019); Thring and Weitz (2006); Mintsa Mi Nzue (2009)	5
*Turbina oblongata* A. Meeuse. Synonyms: *Ipomoea lambtoniana* and *Ipomoea oblongata* E. Mey. ex Choisy	Honeysuckle tree (E) and ubhoqo (Z)	Convolvulaceae	Herb	Leaves and roots	Leaves are used as poultices for swollen joints, sores, and abscesses. Medicine taken internally for rheumatism and gout. Ground root decoctions are taken three times a day for arthritis and gout. Enemas made from roots are given for pain of the spine	Hutchings et al. (1996); Pujol (1990); Polori et al. (2018)	3
*Vachellia karroo* (Hayne) Banfi & Galasso. Synonyms: *Acacia karroo* and *Acacia inconflagrabilis* Gerstner	Sweet thorn (E), soetdoring, doringboom (A), mooka (TW), Muunga (V), Ingamazi, Ingamazi elincane, Umunga, Umantungane, and Usidlodlo (Z)	Fabaceae	Tree	Bark, thorns, leaves, and roots, ns	Analgesic (sharp internal body pains). Bark is used to treat aching legs. Used for stomachache. Thorn decoction is drunk to relieve heart pains. Gum is eaten for stomach ailments and toothache. Bark infusion used to treat stomachache, pain, and inflammation. Root infusion is used to treat swollen and burning feet	Mhlongo and Van Wyk (2019); De Beer and Van Wyk (2011); Watt and Breyer-Brandwijk (1962); Arnold and Gulumian (1984); Hulley and Van Wyk (2019)	5
*Vangueria infausta* Burch. Synonyms: *Canthium infaustum* (Burch.) Baill. and *Vangueria tomentosa* Hochst.	wild medlar (E); wilde mispel (A), Mabilo (SL), Amaviyo, and Umtulwa (Z)	Rubiaceae	Tree	Bark and roots, ns	Analgesic (internal side pains and chest side pains in infants). Bark and roots are medicine for alleviating toothache. Used as steam bath for treating painful body	Mhlongo and Van Wyk (2019); Shai et al. (2020); Mbanjwa (2020)	3
*Volkameria glabra* (E.Mey.) Mabb. & Y.W.Yuan. Synonyms: *Clerodendrum glabrum* E.Mey, *Siphonanthus glaber* (E.Mey.) Hiern, and *Premna suaveolens* Chiov.	Tinderwood, verbena tree, white eat’s whiskers (E), tontelhout, bitterblaar, bontelhout, harpuisblaar, huilboom (A), munukha-tshilongwe (V), umqangazane, umqaqongu, and umqoqongo (Z)	Lamiaceae	Shrub	Leaves and roots, ns	Hot water infusions from roots mixed with those of *Tetradenia riparia* are taken as emetics for dropsy and rheumatic conditions. Leaves are used for toothache. Analgesic (toothache)	Watt and Breyer-Brandwijk (1962); Hutchings et al. (1996); Mhlongo and Van Wyk (2019)	3
*Warburgia salutaris* (G.Bertol.) Chiov. Synonyms: *Chibaca salutaris* and *Warburgia breyeri* R.Pott	Fever tree, pepper-bark tree (E), koorsboom, peperbasboom (A), mulanga (V), amazwecehlabayo, isibaha, and isibhaha (Z)	Canellaceae	Tree	Bark, leaves, and roots	Bark is used in emetics or purgatives for febrile complaints and for rheumatism. Lotions made from pounded leaves with stalks of *Hibiscus surattensis* are applied to the penis for inflammation of the urethra, sores, and other irritations. Powdered roots are applied to oral cavity to relieve toothache. Decoction of the bark is taken for backache	Hutchings et al. (1996); Corrigan et al. (2011); Arnold and Gulumian (1984)	3
*Withania somnifera* (L.) Dunal. Synonyms: *Physalis somnifera* and *Withania microphysalis*	Wilde-appelliefie (A), Winter Cherry, Poisonous Gooseberry (E), Ubuvuma (X), Impathampatha, Ubuvimba Umaqhunsula, and Ubuvumba (Z)	Solanaceae	Shrub	Leaves and roots	Leaf poultices are applied externally to treat rheumatism. Musculoskeletal (inflammation). For treating inflammation. Leaf infusions are used to treat stomach ailments	Hutchings et al. (1996); Mhlongo and Van Wyk (2019); Maroyi (2017); Van Wyk et al. (2008); Hulley and Van Wyk (2019)	5
*Ximenia caffra* Sond.	Plum, large sourplum (E), Kleinsuurpruim (A), Mutanwadombo, Mutshili (V), and Umthunduluka-obomvu (Z)	Olacaceae	Tree	Leaves and roots	Cold leaf infusions are applied to inflamed eyes. Root decoction is used to prepare soft porridge for headache due to indigestion	Watt and Breyer-Brandwijk (1962); Mabogo (1990); Arnold and Gulumian (1984)	3
*Zantedeschia aethiopica* (L.) Spreng. Synonyms: *Arodes aethiopicum* (L.) Kuntze and *Colocasia aethiopica* (L.) Link	White or common arum lily (E); wit varkoor (A), Ingquthuyengane, and Intebe (Z)	Araceae	Herb	Leaves, ns	Musculoskeletal inflammation. Compress leaves are used for treating backache, rheumatism, headache, pain, and inflammation. Treatment for arthritis	Mhlongo and Van Wyk (2019); Hulley and Van Wyk (2019); Philander (2011)	3
*Zanthoxylum capense* (Thunb.) Harv. Synonyms: *Fagara magaliesmontana* Engl. and *Zanthoxylum thunbergii* var. *obtusifolia* Harv.	Adelaide spice tree, cardamom (E), kleinperdepram (A), Umabelejongosi, isinungwane, and umlungumabele (Z)	Rutaceae	Tree	Leaves, bark (root-bark), and roots	Leaves are used to heal sores. Dried ground root-bark is directly applied for toothache. Analgesic (general body pains). An infusion of the root is taken to treat toothache. For treating swollen feet and toothache	Bryant (1966); Hutchings et al. (1996); Mhlongo and Van Wyk (2019); Corrigan et al. (2011); Philander (2011); Mbanjwa (2020)	6
*Zanthoxylum davyi* Waterm. Synonyms: *Zanthoxylum thunbergii* var. *grandifolia* Harv. and *Fagara davyi* I.Verd	Fever Tree, Forest Knobwood (E), Knopdoring, Knopdoringhout (A), Monokwane (NS), Munungu, Murandela (V), Umlungumabele (X), and Umnungumabele (Z)	Rutaceae	Tree	Roots and leaves	For tooth removal. Root decoction is drunk thrice daily for 3 days to relieve chest pains. Powdered leaves are rubbed on chest to relieve pains	Mhlongo and Van Wyk (2019); Mabogo (1990); Mbanjwa (2020)	3
*Ziziphus mucronata* Willd. Synonyms: *Ziziphus madecassus* H. Perrier and *Ziziphus mucronata* subsp. *mucronata*	Blinkblaarboom (A) Buffalo thorn, Cat-thorn (E), Umphafa (X), Mutshetshete, Mukhalu (V), isilahla, umhlahlankosi, and umphafa (Z)	Rhamnaceae	Tree	Bark, roots, and leaves	Bark decoctions are used for rheumatism. Roots are used for toothache. Leaves and roots are used for pain by the Vhavenda. Analgesic (sharp internal body pains). For treating chest pain. Root decoction is used to prepare soft porridge to relieve general body pains	Mabogo (1990); Mhlongo and Van Wyk (2019); Maroyi (2017); Arnold and Gulumian (1984)	4

### Data Mining

**FIGURE 1:**
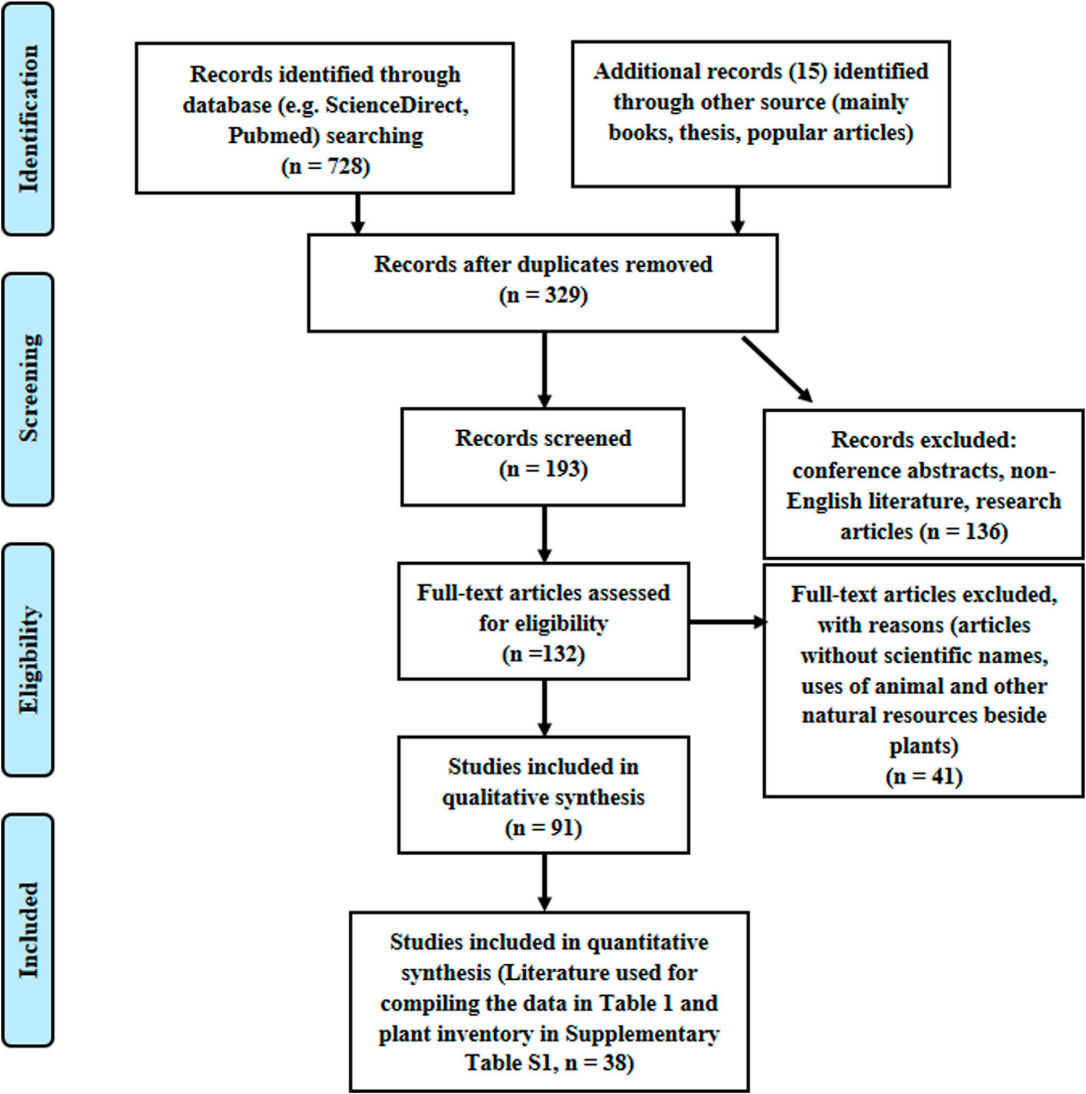
Flow diagram for selection of articles/books/theses included in the systematic literature review for compiling data in Table 1 and Supplementary Table S1.

Following the exclusion of duplicates, citations in abstract form, and non-English citations, the titles/abstracts of full papers were screened for relevance to the scope of the current review (Figure 1). This task was initially conducted by the first author and confirmed by the second author. From each article, the following information was collected: scientific names, family, plant parts, method of preparation, and inflammation or related conditions treated.

Given the importance and the need for accurate scientific nomenclature for plants (Rivera et al., 2014), all scientific names and families were validated in reference to PlantZAfrica (pza.sanbi.org), South African National Biodiversity Institute website (http://newposa.sanbi.org/sanbi/Explore), and the World Flora Online (http://www.worldfloraonline.org/). The synonyms and common names were retrieved from PlantZAfrica and South African National Biodiversity Institute (SANBI) Red List of South African Plants (redlist.sanbi.org/species). Based on the reference database we used for the verification of the botanical names, we opted for the use of family names Asteraceae (PlantZAfrica), Fabaceae (PlantZAfrica, World Flora Online), and Xanthorrhoeaceae (World Flora Online) instead of Compositae, Leguminosae, and Asphodelaceae, respectively.

## Results and Discussion

### Appraisal of Ethnobotanical Literature and Study Locations

Several relevant internet search engines were mined for information relating to ethnobotanical documentation of plants used for treating pain and inflammatory-related conditions in South Africa in the past few decades. Despite the hits from the initial search, the majority were discarded for diverse reasons such as duplicates and being outside the predefined inclusion criteria (Figure 1). Based on the systematic search, 38 pieces of literature (books 29%, theses 10%, and articles 61%) were included as primary data as summarized in Table 1 and detailed in Supplementary Table S1. All the data were extracted from literature documenting general ethnobotanical surveys/studies.

**FIGURE 2:**
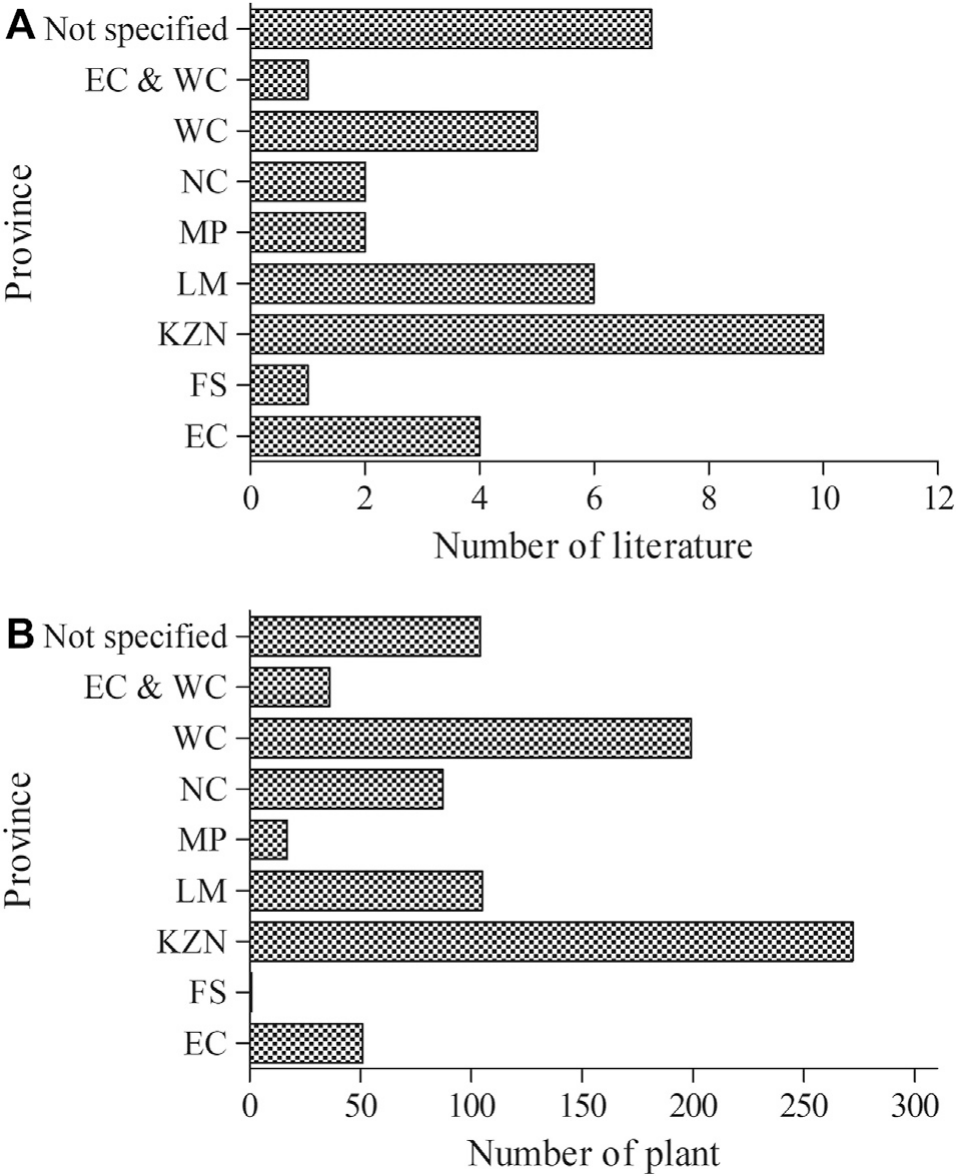
Distribution of literature and plants indicated as a remedy for pain and inflammatory-related conditions across provinces in South Africa. EC = Eastern Cape, FS = Free State, KZN = KwaZulu-Natal, LM = Limpopo, MP = Mpumalanga, NC = Northern Cape, and WC = Western Cape.

Even though about 19% of the studies in the current review were missing specific provinces in South Africa, the majority were linked to KwaZulu-Natal (27%), Limpopo (16%), and Western Cape (14%) provinces (Figure 2). A similar pattern was observed in terms of the high quantity of the plants associated with the different locations. Recently, Ndhlovu et al. (2021) identified the Western Cape, KwaZulu-Natal, and Limpopo provinces as regions with a high record of plants used for the management and treatment of childhood diseases in South Africa. Based on extensive bibliometric analysis of medicinal plant research in South Africa (Viljoen et al., 2019), KwaZulu-Natal was designated as one of the most research active provinces and considered as a “sweet-spot” which may be attributed to the rich plant heritage and indigenous knowledge practice among the Zulus that are the dominant ethnic group.

Evidence from the appraisal of ethnobotanical surveys has revealed the existing gaps across provinces and South African ethnic groups in terms of documenting plants (and associated indigenous knowledge) used for different conditions such as cancer (Twilley et al., 2020), malaria (Cock et al., 2019), childhood diseases (Ndhlovu et al., 2021), respiratory diseases (Cock and Van Vuuren 2020a, b), and animal diseases (McGaw et al., 2020).

### Diversity of Plants, Symptoms/Conditions Treated, Recipes, and Preparations

Based on the 38 pieces of literature included in the current review, 495 plants are used for managing pain and inflammatory-related conditions in South African traditional medicine (Supplementary Table S1). Approximately 18% (87 plants) of the recorded plants had ≥3 mentions/citations suggesting their relative popularity as a remedy for pain and inflammatory-related conditions (Table 2). Particularly, *Ricinus communis* L. (10), *Aloe ferox* Mill. (8), *Pentanisia prunelloides* subsp. *latifolia* (Hochst.) Verdc. (8), *Dodonaea viscosa* Jacq var. *angustifolia* (L.f) Benth. (8), *Gomphocarpus fruticosus* (L.) W.T.Aiton. (7) *Ruta graveolens* L. (7), and *Solanum aculeastrum* Dunal. (7) were the top seven plants with the highest number of mentions/citations.

Symptoms such as swelling, disturbance of normal functions of different body parts, and pains are the hallmark of inflammations (Kuprash and Nedospasov 2016). Even though inflammation is often not a primary cause, it plays an important role in the development of many diseases which has resulted in more targeted efforts at suppressing inflammation as a means of improving clinical conditions (Hotamisligil 2006; Kuprash and Nedospasov 2016). In traditional medicine, healing is holistic in nature whereby symptoms are often the focus during treatment of many diseases (Iwalewa et al., 2007; Adebayo and Amoo 2019; Cock et al., 2019; Elgorashi and McGaw 2019; Twilley et al., 2020; Khumalo et al., 2021). This possibly accounted for the high number of plants prescribed for managing pains and inflammatory-related conditions (Supplementary Table S1). Apart from the use of the term “pain(s)” reliever associated with many of the identified plants, other widely mentioned conditions were headache (142), toothache (114), backache (80), abdominal pain (17), menstrual/period pains (27), rheumatism (78), stomachache (64), swelling/inflammation (113), and sprain (14).

Apart from the use of a single plant, a combination of plants was common for treating pain and inflammation-related conditions. Some examples involved the combination of *Athrixia phylicoides* DC. and *Athrixia elata* Sond. as decoctions for relieving sore feet (Watt and Breyer-Brandwijk 1962). *Brackenridgea zanguebarica* powdered roots are rubbed on after treatment with *Aloe chabaudii* Schönland to relieve swollen ankles (Arnold and Gulumian 1984). As recorded by Bryant (1966), the root decoction of *Gunnera perpensa* L. is used in conjunction with other plants such as *Alepidea amatymbica* Eckl. & Zeyh. and *Crinum* species as a remedy for pain in rheumatic fever and stomachache. This phenomenon of combining two or more plants for treating different diseases conditions has been well demonstrated in African traditional medicine (Alebie et al., 2017; Ahmed et al., 2018). Apart from the combination with other plants, Bhat (2014) indicated that *Aloe arborescens* Mill. leaves are mixed with chicken feed as anti-inflammatory herb among the indigenous people of Eastern Cape, South Africa.

In terms of preparation, decoctions and infusions were the dominant methods used for preparing the plants used for treating pain and inflammatory-related conditions (Supplementary Table S1). These aforementioned methods are generally regarded as the most common preparation methods in traditional medicine as evident in recent studies (Boadu and Asase 2017; Alamgeer et al., 2018; Masondo et al., 2019; Aumeeruddy and Mahomoodally 2020; Ndhlovu et al., 2021). When compared to other preparation methods, the relatively shorter duration required for making medicinal plant decoctions is surely the desired benefit (Aumeeruddy and Mahomoodally 2020). Despite the popularity of decoctions and infusions, variations (e.g., duration of boiling, types, and volume of solvents) often exist in their preparations among traditional healers and locations, which remain a concern for standardization and reproducibility (Boadu and Asase 2017). Other methods of preparation and application have been recorded for mitigating pain and inflammatory-related conditions in South Africa. For instance, the application of leaf as a compress remains common among different ethnic groups for pain and backache (Supplementary Table S1). Particularly, warm leaves of *Ricinus communis* L. are wrapped around the stomach as a remedy for stomachache (Mbanjwa 2020). In addition, fresh leaves of *Centella asiatica* Urb. are used as ear plugs to relieve ear pain (Van Wyk et al., 2008) while dried ground root-bark of *Zanthoxylum capense* (Thunb.) Harv. is directly applied for toothache (Hutchings et al., 1996).

Other trends observed were the variations in the use of specific plants for children relative to adults and on the basis of gender (Supplementary Table S1). Infusion made from the bark of *Berchemia zeyheri* (Sond.) Grubov. is administered as enemas for pains and for rectal ulceration in children (Watt and Breyer-Brandwijk 1962). Whole plant decoction made from *Acanthospermum hispidum* DC. and warm leaves of *Ricinus communis* L. is used to treat stomachache in children (Bhat 2013; Mbanjwa 2020). *Asparagus laricinus* Burch. and *Berkheya bipinnatifida* (Harv.) Roessler is known as an effective remedy for different types of pains in children among the Zulus (Mhlongo and Van Wyk 2019). In Limpopo, *Rhoicissus tridentata* (L.f.) Wild & R.B.Drumm. powdered roots are added to porridge to relieve stomach pain in children (Arnold and Gulumian 1984). Maceration of the powdered roots of *Cassine transvaalensis* (Burtt Davy) Codd is drunk to relieve stomachache in male (Arnold and Gulumian 1984). Evidence from the current review revealed that several plants (e.g., *Aloe arborescens* Mill, *Acokanthera oppositifolia* (Lam.) Codd., *Chironia baccifera* L., *Gnidia capitata* L.f., *Mentha spicata* L., and *Pelargonium hypoleucum* Turcz.) are frequently used for relieving menstrual pains in females (Hutchings et al., 1996; Philander 2011; Nortje and Van Wyk 2015; Mbanjwa 2020). However, the cultural implications and explanations for these significant observations are not explicitly documented.

### Plant Families Used for Pain and Inflammatory-Related Conditions

**FIGURE 3:**
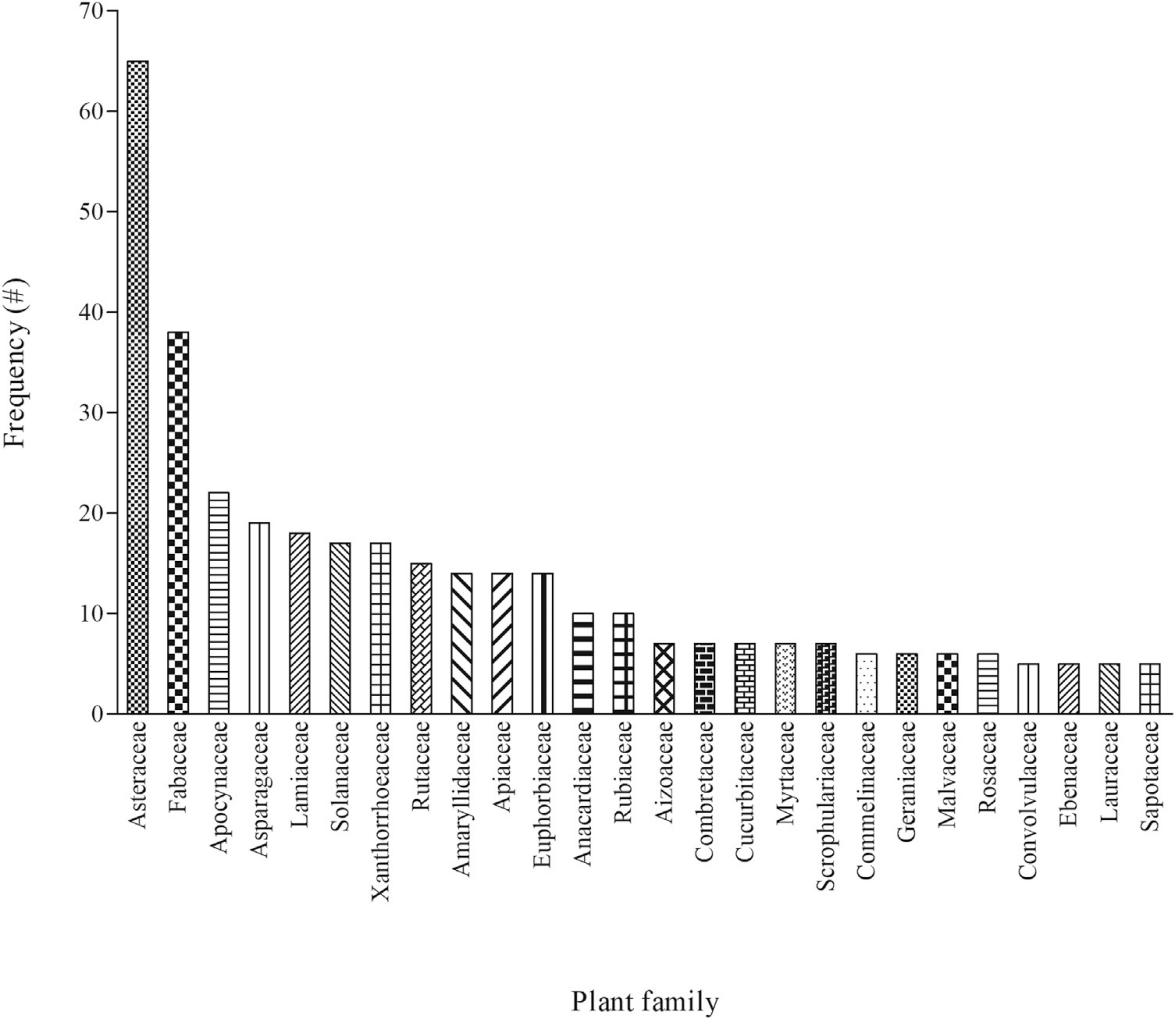
Distribution of families with five or more plants (n ≥ 5) indicated as a remedy for pain and inflammatory-related conditions among different ethnic groups in South Africa, # = number of mentions.

The 495 recorded plants were distributed into 99 families with Asteraceae (13%), Fabaceae (8%), Apocynaceae (4.3%), Asparagaceae (4%), and Lamiaceae (4%) having the highest number of plant species. Other highly represented families included Xanthorrhoeaceae, Solanaceae, Rutaceae, Euphorbiaceae, Apiaceae, and Amaryllidaceae (Figure 3). The majority of these aforementioned families remain dominant in African traditional medicinal flora (Van Wyk 2020). On the other hand, 64 of the 99 families (e.g., Acanthaceae, Araceae, Brassicaceae, Clusiaceae, Ruscaceae, and Portulacaceae) were poorly represented having an average of 1–2 plants per family (Supplementary Table S1). The high utilization of plant families such as Fabaceae and Asteraceae in folk medicinal flora has been well pronounced for different disease conditions. For instance, a detailed review of ethnobotanical surveys revealed that Asteraceae is the most cited family used for treating childhood diseases in South Africa (Ndhlovu et al., 2021), medical ethnobotany of Lesotho (Moteetee and Van Wyk 2011), medicinal plants used as blood purifiers in southern Africa (van Vuuren and Frank 2020), and managing respiratory infections and related symptoms in South Africa (Semenya and Maroyi 2018; Cock and Van Vuuren 2020a) as well as in Pakistan (Alamgeer et al., 2018).

Fabaceae is regarded as the most dominant plant family used for treating malaria in Ethiopia (Alebie et al., 2017). Based on Moerman’s approach, Muleba et al. (2021) identified Fabaceae as the overutilized families for medicinal purpose in Mpumalanga province of South Africa. Following a detailed family-level floristic analysis of medicinal plants, the African folk medicine is dominated by Fabaceae, Asteraceae, and Rubiaceae, which are considered as the top three dominant families in African medicinal flora (Van Wyk 2020). In the current review, both Asteraceae and Fabaceae accounted for approximately 21% (102) of the plants used for treating pain and inflammatory-related conditions in South Africa (Figure 3). As one of the largest plant families globally, the high abundance (about 32 913 species) and distribution of the Asteraceae probably translate to higher availability and utilization as medicinal flora in African folk medicine (Van Wyk 2020). Given that some members of the Asteraceae occur as weeds and early colonisers in fields especially after anthropogenic activities (Daehler 1998), this may be responsible for the ease of access and availability especially in home garden as medicine among local communities (Alamgeer et al., 2018). In addition, the presence of therapeutic chemicals such as alkaloids and terpenoids has been proposed as the driving factor for the utilization of families such as Fabaceae and Apocynaceae in folk medicine (Van Wyk 2020).

### Distribution of Life-Form and Plant Parts

**FIGURE 4:**
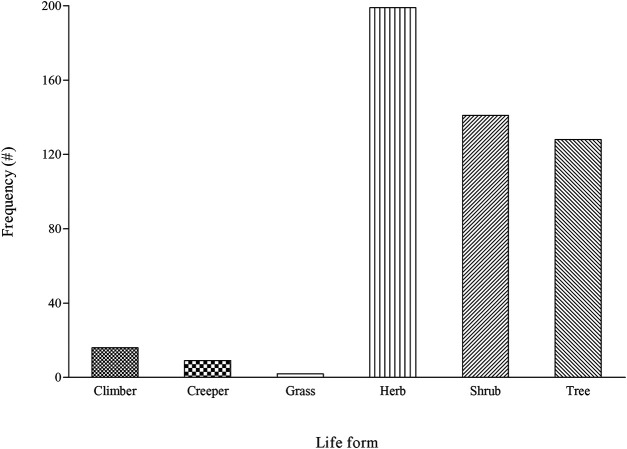
An overview of life-form for plants indicated as a remedy for pain and inflammatory-related conditions among different ethnic groups in South Africa, # = number of mentions.

In terms of life-form, approximately 54% of the recorded plants were woody consisting of trees and shrubs while herbs contributed 40% (Figure 4), a similar pattern with the dominance of woody plants and herbs in South African traditional medicine (Maroyi 2017; Asong et al., 2019). Given the wide variation in rainfall patterns in South Africa (Kruger and Nxumalo 2017), the type and life-form of plants occurring across the different ecological zones are predictable. Furthermore, the presence and occurrence of the plant species often determine their utilization for medicinal and other purposes among local communities (Muleba et al., 2021).

**FIGURE 5:**
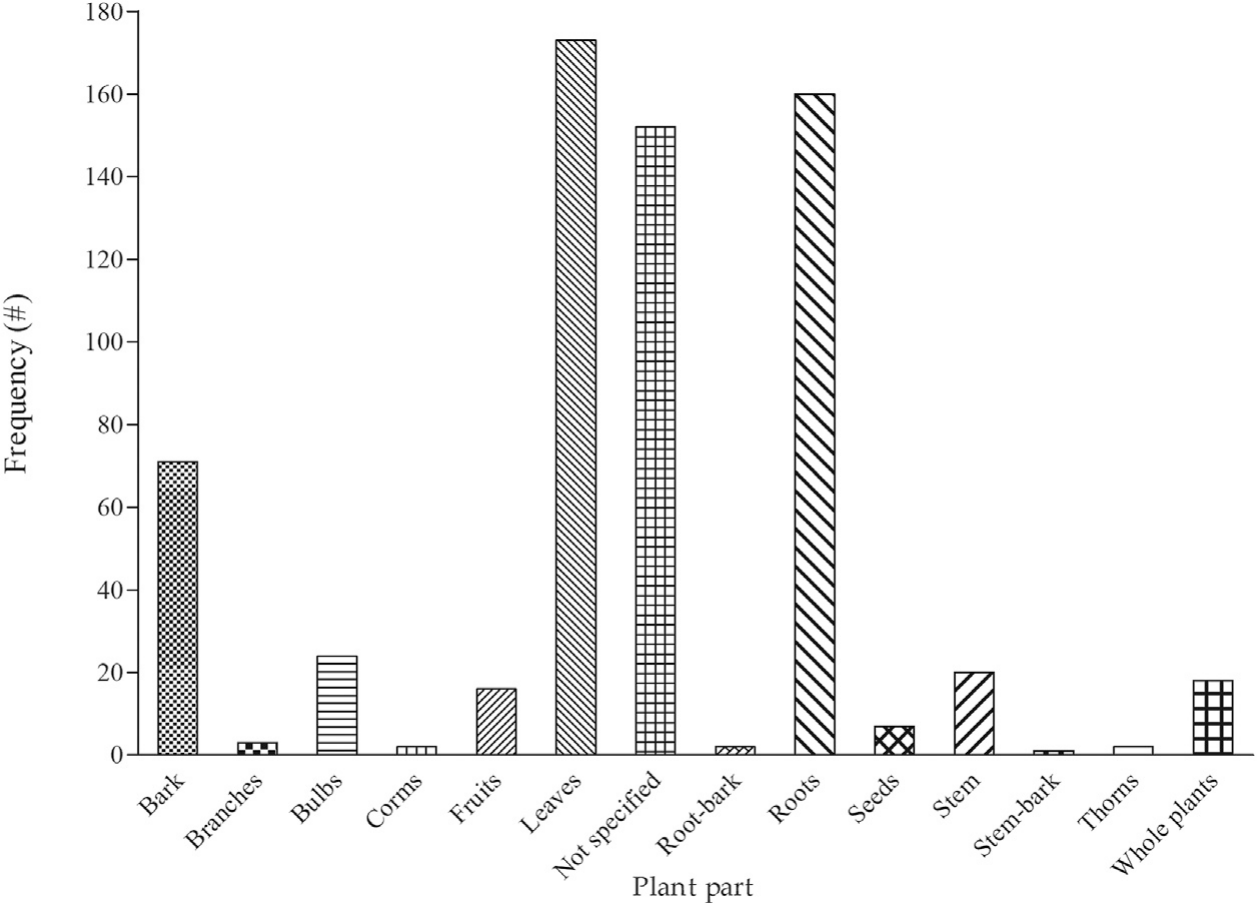
Distribution of plant parts indicated as a remedy for pain and inflammatory-related conditions among different ethnic groups in South Africa, # = number of mentions.

In many instances, more than one part of the 495 recorded plants were used for the treatment of pain and inflammatory-related conditions (Supplementary Table S1). Even though there were significant cases (24%) whereby the authors did not identify plant parts, more than 10 different parts were generated in the current review (Figure 5). Among the identified plant parts, the leaves (27%), roots (25%), and bark (11%) were the most dominant while the use of seeds, branches, corms, stem-bark, root-bark, and thorns was generally limited (<2%). The current observation whereby the leaves were the dominant plant part remains a common pattern as evident in the recent appraisal of ethnobotanical surveys in southern Africa (Semenya and Maroyi 2018; Ndhlovu et al., 2021). Particularly, the leaves were the most frequently used plant part for the treatment of bacterial (Cock and Van Vuuren 2020a) and viral (Cock and Van Vuuren 2020b) respiratory infections across the traditional southern African healing systems. On a global scale, Aumeeruddy and Mahomoodally (2020) identified leaves as the most used plant part in the management of hypertension in traditional medicine. The general popularity of the leaves with respect to other plant parts may be related to ease of access and high abundance in many communities (Maroyi 2017; Aumeeruddy and Mahomoodally 2020). From a conservation perspective (Moyo et al., 2015), the preference of leaves and other aerial parts of the plants for medicinal purposes is advantageous and often strongly recommended. When compared to other plant parts such as roots and bark, the leaves regenerate faster and exert lesser strain on local populations of important medicinal plants (Alebie et al., 2017). As a result, different conservation strategies especially plant-part substitution (e.g., leaves instead of roots and bark) have been often suggested to stakeholders (Lewu et al., 2006; Moyo et al., 2015).

## Conclusion

Based on the large number (495) of plants recorded, it is evident that their utilization for managing pain and inflammatory-related conditions remains a common practice in South African folk medicine. An estimated 18% (87 of the 495) of the recorded plants are relatively well known given that they were mentioned by three or more sources in South Africa. The recorded plants were utilized among the different ethnic groups for a wide range of conditions especially pains, headache, toothache, and backache. We observed that some of the plants recorded in the current study are strictly prescribed based on gender and age (children versus adults). In some cases, we observed that important information such as the plant part utilized, preparation methods, and recipes for a significant portion of identified plants were missing in the existing literature. This underscores the limited value of the existing fragmented nature of the ethnobotanical surveys in South Africa. In order to mitigate these challenges, adherence to the established guidelines and robust ethnobotanical research methodology remains essential for the development of a holistic inventory relating to remedies used for pain and inflammatory-related conditions.

## Data Availability

The original contributions presented in the study are included in the article/Supplementary Material; further inquiries can be directed to the corresponding author.
