# Supplementary material for: Medicinal Plants for Mitigating Pain and Inflammatory-Related Conditions: An Appraisal of Ethnobotanical Uses and Patterns in South Africa
Source: Front Pharmacol. 2021 Oct 22;12:758583. doi: 10.3389/fphar.2021.758583 (PMC8569556; doi:10.3389/fphar.2021.758583)
Supplement: Supplementary file 1 [file Table1.pdf]

Supplementary Table S1: An inventory of 495 plants used for mitigating pain and inflammatory-related conditions in South Africa. Botanical names were verified using PlantZAfrica (pza.sanbi.org), South African National Biodiversity Institute website (http://newposa.sanbi.org/sanbi/Explore) as well as the World Flora Online (http://www.worldfloraonline.org/). *Common name: A = Afrikaans; E = English; K= Khoi; KS = Khoisan (Khoe-San), SS = Southern Sotho; SL = Sepulana; SP = Sepedi; NS = Northern Sotho; TW = Twana; X = Xhosa; V= Vhenda; Z = Zulu. #Part used: ns = not specified. Nm =number of mentions/citations

**Botanical name**	***Common name**	**Family**	**Life-form**	**#Part used**	**Application**	**Reference**	**Nm**
Abrus precatorius L. Synonyms: *Abrus maculatus* Noronha, *Abrus squamulosus* E.Mey.	Bead vine, coral bead plant, coral bean (E), Umkhokha (Z)	Fabaceae	Climber	ns	Analgesic (sharp internal body pains)	Mhlongo and Van Wyk (2019)	1
Abutilon fruticosum Guill. & Perr.	Shrubby Abutilon (E)	Malvaceae	Herb	Whole plant	Root decoction is used for cramps	Tshikalange et al. (2016)	1
Acacia dealbata Link. Synonyms: *Acacia decurrens* var. *dealbata* (Link) Muller, *Racosperma dealbatum* (Link) Pedley	Mimosa, Silver wattle (E), Uwatela (Z)	Fabaceae	Tree	ns	Analgesic (toothache). Used for period pains	Mhlongo and Van Wyk (2019); Mbanjwa (2020)	2
Acacia mearnsii De Wild. Synonyms: *Racosperma mearnsii* (De Wild.) Pedley, *Acacia decurrens* var. *mollis* Lindl.	Black wattle (E), Uwatela (Z)	Fabaceae	Tree	Bark, ns	Analgesic (toothache). Ground bark is given with water thrice daily for stomachache	Mhlongo and Van Wyk (2019); Bhat (2014)	2
Acalypha glabrata Thunb. var. *glabrata* Synonyms: *Acalypha betulina* E.Mey., *Ricinocarpus glabratus* (Thunb.) Kuntze	Forest False Nettle, Forest Mock Nettle (E), Isithobothi, Uthobothi (Z)	Euphorbiaceae	Shrub	ns	Analgesic (general body pains)	Mhlongo and Van Wyk (2019)	1
Acalypha glandulifolia Buchinger & Meisn. ex C.Krauss Synonyms: *Acalypha entumenica* Prain, *Acalypha peduncularis* var. *glandulifolia* (Buchinger & Meisn. ex C.Krauss) Müll.Arg.	Umsunundu (Z)	Euphorbiaceae	Herb	ns	Musculo-skeletal (inflammation)	Mhlongo and Van Wyk (2019)	1

Botanical name	*Common name	Family	Life-form	#Part used	Application	Reference	Nm
Acanthospermum hispidum DC. Synonyms *Acanthospermum humile* var. *hispidum* (DC.) Kuntze	Upright starbur, goat's head (E), inkunaana (X)	Asteraceae	Herb	Whole plant	Whole plant decoction is used to treat stomachache in children	Bhat (2013)	1
Acokanthera oblongifolia (Hochst.) Codd Synonyms: *Acokanthera spectabilis* (Sond.) Hook.f., *Carissa oblongifolia* Hochst.	Dune poison-bush (E), Inxinebe (X), Inhlungunyemba, Inhlungunyembe (Z)	Apocynaceae	Shrub	Leaves, ns	Headache, analgesic (headache, general pains, toothache), musculo-skeletal inflammation	Maroyi (2017); Mhlongo and Van Wyk (2019)	2
Acokanthera oppositifolia (Lam.) Codd. Synonyms: *Acokanthera venenata*, *Carissa acokanthera*	Bushman's arrow poison, Hottentot's poison bush, kaffir poison bush, poison bush/tree (E), iNxinene (X), ubuhlungu-benyoka, umkhwangu, Inhlungunyembe (Z)	Apocynaceae	Shrub	Leaves, roots	For painful feet, rheumatism, toothache, abnormal menstrual period and swellings. Analgesic (headache, general pain, sharp internal body pains). Powder made from the dry roots is used as stuff for headache	Hutchings et al. (1996); Watt and Breyer-Brandwijk (1962); Mhlongo and Van Wyk (2019); Bhat and Jacobs (1995)	4
Acorus calamus L. Synonyms: *Acorus angustifolius* Schott, *Acorus verus* (L.) Raf.	Sweet-flag (E), makkalmoes (A), Indawolucwatho, ikalamuzi (Z)	Acoraceae	Herb	ns	Used for period pains	Mbanjwa (2020)	1
Adansonia digitata L. Synonyms: *Adansonia bahobab* L., *Baobabus digitata* (L.) Kuntze	Baobab, Cream of Tartar tree, monkey-bread tree, lemonade tree (E), kremetartboom(A), mowana (TW), muvhuyu (V), isimuku, umShimulu, isiMuhu (Z)	Malvaceae	Tree	Leaves	Cooked leaves are eaten by women as potherb at the same time acting as the period pains reliever	Mokganya and Tshisikhawe (2019)	1
Adenandra uniflora (L.) Willd. Synonyms: *Adenandra pottsii* Dummer, *Glandulifolia uniflora* (L.) J.C.Wendl.	China flower, shepherd's delight (E), Kommetjie teewater, bergskaapboegoe (A)	Rutaceae	Shrub	Leaves	Remedy for pains	Mintsa Mi Nzue (2009)	1

Botanical name	*Common name	Family	Life-form	#Part used	Application	Reference	Nm
Adenia digitata (Harv.) Engl. Synonyms: *Adenia angustisecta* Burtt Davy, *Modecca digitata* Harv.	Wild granadilla (E), dundu (V)	Passifloraceae	Climber	Roots	Root decoction is drunk and applied externall to swollen legs	Arnold and Gulumian (1984)	1
Adenia gummifera (Harv.) Harms. Synonyms: *Adenia rhodesica* Suess., *Modecca gummifera* Harv.	Wild Grenadilla, Snake-climber, Monkey Rope (E), Slangklimop (A), Impinda, Umphindamshaya, Impindamshaye (Z)	Passifloraceae	Climber	Vine, bark	For treating body pains and headache. Used to cure pain in shoulders and between shoulder blades (*isibhobo*). Bark is used for period pains	Philander (2011); Mbanjwa (2020)	2
Adromischus triflorus (L.f.) A.Berger. Synonyms: *Adromischus procurvus* (N.E.Br.) C.A.Sm., *Cotyledon triflora* L.f.	duck's foot, calico heart (E), bontplakkie, eendevoet (A)	Crassulaceae	Herb	Leaves	Leaf juice is used for ear-ache (otitis) and toothache	Hulley and Van Wyk (2019)	1
Afroaster hispida (Thunb.) J.C. Manning & Goldblatt. Synonyms: *Aster asper* J.M.Wood & M.S.Evans, *Aster bakerianus* Burtt Davy ex C.A.Sm.	Udlutshana, Udlutshani (Z)	Asteraceae	Shrub	ns	Musculo-skeletal (back problem)	Mhlongo and Van Wyk (2019)	1
Afrocanthium mundianum (Cham. & Schltdl.) Lantz. Synonyms: *Canthium mundianum* Cham. & Schltdl., *Plectronia mundiana* (Cham. & Schltdl.) Pappe	Rock-alder (E), Klipels (A), mutomboti (V)	Rubiaceae	Tree	Roots	Root maceration is drunk for abdominal pains	Arnold and Gulumian (1984)	1
Agathosma betulina (Berg.) Pillans. Synonyms: *Diosma betulina* Thunb., *Bucco betulina* Schult.	Long-leaf buchu (E), langblaar boegoe (A), buchu (K), ibuchu (X)	Rutaceae	Shrub	Leaves	For relieving the symptoms of rheumatism and easing backache. Dried leaves are used to treat stomachache. Infusions used for arthritis, inflammation and backache. Applied externally for sprains. For sprains and pains	Watt and Breyer-Brandwijk (1962); De Beer and Van Wyk (2011); Hulley and Van Wyk (2019); Philander (2011); Thring	6

Botanical name	*Common name	Family	Life-form	#Part used	Application	Reference	Nm
					(including arthritic pain), the dried leaves are placed on a cloth and sprinkle with brandy or vinegar and wrap cloth around affected area to relieve the pain	and Weitz (2006); Mintsa Mi Nzue (2009)	
Agathosma imbricata (L.) Willd. Synonyms: *Agathosma lambii* Dummer, *Hartogia ciliata* P.J.Bergius	Sand buchu (E), sandboegoe (A)	Rutaceae	Shrub	Leaves	Applied externally for sprains	Philander (2011)	1
Agathosma odoratissima (Montin) Pillans. Synonyms: *Barosma alpina* Eckl. & Zeyh., *Diosma odoratissima* Montin	Breëblaarboegoe (A)	Rutaceae	Shrub	Leaves	Applied externally for sprains	Philander (2011)	1
Agathosma ovata (Thunb.) Pillans. Synonyms: *Agathosma acuminata* Drège ex Walp., *Barosma lanceolata* Sond.	Kluitjieskraal false buchu (E), Kluitjieskraalbasterboegoe (A)	Rutaceae	Shrub	ns	Treatment of back pain	Van Wyk et al. (2008)	1
Agave americana L. Synonyms: *Agave americana* var. *marginata* Trel., *Aloe americana* (L.) Crantz	American agave, American aloe, century plant (E), Amerikaanse aalwee, Amerikaanse aalwyn, blou-aalwee, blougaringboom, gareboom, makaalwyn (A),	Asparagaceae	Herb	Leaves	Leaves used as a poultice for pain and inflammation, sprained ankle and rheumatism	Hulley and Van Wyk (2019)	1
Albizia adianthifolia (Schumach.) W.Wight. Synonyms: *Albizia adianthifolia* var. *adianthifolia*, *Mimosa adianthifolia* Schum.	Flat-crown albizia, rough-bark flat-crown (E), platkroon (A), iGowane, Usolo, umNebelele (Z), umHlandlothi (X)	Fabaceae	Tree	Bark	Infusions of the bark are taken orally to treat arthritis. Analgesic (backache, headache)	Van Wyk and Gericke (2000); Mhlongo and Van Wyk (2019)	2
Alepidea amatymbica Eckl. & Zeyh.	larger tinsel flower (E), kalmoes (A), Iqwili (X), ikhathazo (Z)	Apiaceae	Herb	Rhizomes, roots	Rhizomes are used for rheumatism. Rootstock is used for abdominal pain and headache	Hutchings et al. (1996); Maroyi (2017)	2

Botanical name	*Common name	Family	Life-form	#Part used	Application	Reference	Nm
Alepidea setifera N.E.Br.	Lesokwana (SS)	Apiaceae	Herb	ns	Used to treat toothache	Mogale et al. (2019)	1
Allium sativum L. Synonyms: *Allium longicuspis* Regel, *Porrum ophioscorodon* (Link) Rchb.	Garlic (E), knoffel (A)	Amaryllidaceae	Herb	Bulbs	Cloves are eaten raw. Taken on a daily basis for arthritis and pains, backache and rheumatism	Thring and Weitz (2006)	1
Aloe arborescens Mill. Synonyms: *Aloe arborea* Medik., *Catevala arborescens* (Mill.) Medik.	Krantz aloe (E), kransaalwyn (A), ikalene (X), Inhlabane, Inkalane (Z)	Xanthorrhoeaceae	Shrub	Leaves, ns	Musculo-skeletal inflammation. Small piece of leaves are mixed with chicken feed as anti-inflammatory herb. Two-three spoon of leaf gel is taken orally to treat stomachache. For relieving menstrual pains and poultice for painful feet	Mhlongo and Van Wyk (2019); Bhat (2014); Mbanjwa (2020)	3
Aloe aristata Haw. Synonyms: *Aloe aristata* var. *leiophylla* Baker, *Aloe longiaristata* Schult. & Schult.f.	Lace aloe (E), uMathithibala (Z)	Xanthorrhoeaceae	Herb	Roots	For relieving headache	Mintsa Mi Nzue (2009)	1
Aloe chabaudii Schönland. Synonyms: *Aloe chabaudii* var. *chabaudii*, *Aloe chabaudii* var. *verekeri* Christian	Tshikhopha (V)	Xanthorrhoeaceae	Herb	Leaves	Leaves are cut with razor, warmed on fire and pressed on swollen ankles	Arnold and Gulumian (1984)	1
Aloe dichotoma Masson = *Aloidendron dichotomum* (Masson) Klopper & Gideon F.Sm.	Quiver tree (E); kokerboom (A), Kokerboom (KS)	Xanthorrhoeaceae	Tree	Roots	Root decoction used for treatment of pain. An infusion is used for backache	Nortje and van Wyk (2015); De Beer and Van Wyk (2011)	2
Aloe ferox Mill. Synonyms: *Aloe galpinii* Baker, *Aloe muricata* Haw.	Bitter aloe, red aloe, century tree (E), bitteraalwyn, bergaalwyn (A), iKhala, umHlaba, uNomaweni (X), iNhlaba (Z)	Xanthorrhoeaceae	Shrub	Leaves	Leaves are boiled in water and taken orally for arthritis. Leaf gel is used for stomachache. Leaf infusion is used for back pain. Leaf decoctions (half a cup) is	Watt and Breyer-Brandwijk (1962); Bruce (1975); Maroyi (2017); De Beer and Van Wyk	8

Botanical name	*Common name	Family	Life-form	#Part used	Application	Reference	Nm
					taken orally for stomachache. Leaf infusion is taken orally as a gargle for toothache. For relieving headache	(2011); Bhat and Jacobs (1995); Bhat (2014); Hulley and Van Wyk (2019); Mbanjwa (2020)	
Aloe greatheadii var. *davyana* (Schönland) Glen & D.S.Hardy. Synonyms: *Aloe davyana* Schönland, *Aloe mutans* Reynolds	Spotted aloe (E), Transvaalaalwyn, grasaalwyn (A), kgopane (TW), lekgala, lekgala la Lesotho, lekgala la quthing (SS)	Xanthorrhoeaceae	Shrub	Leaves	Leaf is placed in hot water and used to rub over a sprained ankle/joint	Mogale et al. (2019)	1
Aloe maculata All. Synonyms: *Aloe commutata* var. *bicolor* A.Berger, *Aloe umbellata* DC.	Soap aloe (E), bontaalwyn (A), Icena, Ichenyane, Inhlaba (Z)	Xanthorrhoeaceae	Herb	ns	Musculo-skeletal inflammation. Used as poultice for painful feet	Mhlongo and Van Wyk (2019); Mbanjwa (2020)	2
Aloe microstigma Salm-Dyck. Synonyms: *Aloe brunnthaleri* A.Berger ex Camm., *Aloe juttae* Dinter	Karoo aloe, small-spotted aloe (E), karoo-aalwyn (A)	Xanthorrhoeaceae	Herb	Leaves	Leaves are used to treat back pain	De Beer and Van Wyk (2011); Hulley and Van Wyk (2019)	2
Aloe striata Haw. Synonyms: *Aloe albocincta* Haw., *Aloe rhodocincta* Baker	Coral aloe (E), Blouaalwyn (A)	Xanthorrhoeaceae	Herb	Leaves	Leaves are heated in the fire or in warm ash and apply for leg pain and rheumatism	Van Wyk et al. (2008)	1
Aloe thraskii Baker	Dune aloe (E), Inhlaba yasolwandle, Isigoba (Z)	Xanthorrhoeaceae	Tree	ns	Analgesic (sharp internal pains)	Mhlongo and Van Wyk (2019)	1
Aloe variegata L. = *Gonialoe variegata* (L.) Boatwr. & J.C.Manning. Synonyms: *Aloe ausana* Dinter, *Aloe variegata* var. *haworthii* A.Berger	Partridge breast aloe (E), kanniedood, bontalwyn (A)	Xanthorrhoeaceae	Herb	Leaves	Leaf pulp is used to treat pain. Leaves used as poultice for inflammation, boils and whitlow fingers	De Beer and Van Wyk (2011); Hulley and Van Wyk (2019)	2
Aloidendron barberae (Dyer) Klopper & Gideon F.Sm.= *Aloe bainesii* T.-Dyer. Synonyms *Aloe barberae* Dyer	Tree aloe (E), sigoba, Inhlabende, Inhlaba yesilungu (Z)	Xanthorrhoeaceae	Tree	ns	Analgesic (sharp internal body pains). Used for toothache	Mhlongo and Van Wyk (2019); Mbanjwa (2020)	2

Botanical name	*Common name	Family	Life-form	#Part used	Application	Reference	Nm
Alsophila dregei (Kunze) R.M.Tryon. Synonym: *Cyathea dregei*	Grassland tree fern, common tree fern (E), Inkomankoma, Inkombandlela (Z)	Cyatheaceae	Shrub	ns	Analgesic (sharp internal body pains)	Mhlongo and Van Wyk (2019)	1
Amaranthus spinosus L. Synonyms: *Amaranthus spinosus* var. *basiscissus* Thell., *Galliaria spitosa* (L.) Nieuwl.	Spiny amaranth, spiny pigweed, prickly amaranth (E), Isinyembane (Z)	Amaranthaceae	Herb	ns	Analgesic (sharp internal body pains-*izibhobo*)	Mhlongo and Van Wyk (2019)	1
Ammocharis coranica (Ker Gawl.) Herb. Synonyms: *Amaryllis coranica* Ker Gawl., *Brunsvigia coranica* (Ker Gawl.) Ker Gawl., *Crinum tavelianum* (Schinz) Fritsch	karoo lily, koranna lily, sore eye flower, bible flower, ground lily (E), berglelie, gifbol, seeroogblom (A) isidiya, icukudo, incotho (Z)	Amaryllidaceae	Herb	Bulbs	Bulb is used in the treatment of footache	Mongalo and Makhafola (2018)	1
Aneilema aequinoctiale (P.Beauv.) Loudon. Synonyms: *Aneilema adhaerens* Kunth	Clinging aneilema (E), Idangabane (Z)	Commelinaceae	Shrub	ns	Musculo-skeletal (inflammation)	Mhlongo and Van Wyk (2019)	1
Anemone tenuifolia (L.f.) DC. Synonyms: *Anemone capensis* Lam., *Clematis capensis* Poir.	Cape Anemone, Syblom, Wildanemone, Windflower, black widow (E), Anemoon, Syblom, katiedreiblaar, Veldanemoon (A)	Ranunculaceae	Herb	Leaves	Leaves used for treating toothache and headache	Philander (2011)	1
Anemone vesicatoria (L.f.) Prantl. Synonyms: *Knowltonia vesicatoria* (L.f.) Sims, *Christophoriana vesicatoria* (L.f.) Kuntze	Blisterleaf (E), brandblaar, katjiedrieblaar, tandpynblaar (A)	Ranunculaceae	Herb	Leaves	Fresh leaf infusions are used for rheumatism. Used to treat toothache and headache	Forbes (1986); Hutchings et al. (1996); Hulley and Van Wyk (2019)	3
Anginon difforme (L.) B.L.Burtt. Synonyms: *Bupleurum difforme* L., *Rhyticarpus difformis* (L.) Briq.	Common needle-leaf (E), Pennebos (KS)	Apiaceae	Shrub	Leaves	Leaf decoction is used for backache	Nortje and van Wyk (2015)	1
Anisodontea triloba (Thunb.) D.M.Bates. Synonyms: *Malva*	Wildestokroos (A)	Malvaceae	Herb	Leaves	Leaf infusion is used for headache	De Beer and Van Wyk (2011)	1

Botanical name	*Common name	Family	Life-form	#Part used	Application	Reference	Nm
triloba Thunb., *Malvastrum grossulariifolium* var. *parvifolium* Harv. p.p.							
Annona senegalensis Pers. Synonyms: *Annona arenaria* Thonn. ex Schumach., *Annona chrysophylla* Bojer	African custard-apple, wild custard apple (E), Muembe (V)	Annonaceae	Shrub	Roots, branches	Roots are boiled and the mixture drank for pains during pregnancy. Root maceration is taken for stomachache. Root decoction is used to prepare soft porridge twice daily to relieve headache due to indigestion. Branches are tied around the head to relieve headache	Mahwasane et al. (2013); Arnold and Gulumian (1984)	2
Ansellia africana Lindl.	Leopard orchid (E), Imfe yenkawu, Iphakama (Z)	Orchidaceae	Herb	ns	Analgesic (sharp internal body pains)	Mhlongo and Van Wyk (2019)	1
Antidesma venosum E.Mey. ex Tul. Synonyms: *Antidesma fuscocinereum* Beille, *Minutalia tomentosa* Fenzl	Tasselberry (E), Voëlsitboom, Tosselbessie (A), modulane (NS), Kgôbê-tsabadisana, Moingwe, Segagama (TW), Mufhala-khwali (V), Umtyongi (X), Isiqutwane, Umhlabahlungulu, Umhlalanyoni (Z),	Phyllanthaceae	Tree	ns	For treating headache	Mintsa Mi Nzue (2009)	1
Antizoma miersiana Harv. Synonym: *Cissampelos angustifolia*	Bloubos (KS)	Menispermaceae	Shrub	Leaves, twigs	Leaf and twig decoction used for pains, such as backache and knee pain	Nortje and van Wyk (2015)	1
Aptosimum indivisum Burch. ex Benth. Synonym: *Aptosimum nanum* Engl.	Veld Violet, Wild Violet, Karoo Violet (E), Karooviooltjie (A)	Scrophulariaceae	Herb	Whole plant	Infusion of whole plant is used to treat stomachache, toothache and headache. Used for ear-ache. Leaf infusion is drunk for pain and inflammation	De Beer and Van Wyk (2011); Hulley and Van Wyk (2019)	2

Botanical name	*Common name	Family	Life-form	#Part used	Application	Reference	Nm
Aptosimum procumbens (Lehm.) Burch. ex Steud. Synonyms: *Aptosimum depressum* Burch. ex Benth., *Ohlendorffia procumbens* Lehm.	Carpet flower, Karoo violet (E), kankerbos (A)	Scrophulariaceae	Herb	Leaves	Used as an ointment, as wash for toothache	Hulley and Van Wyk (2019)	1
Aptosimum spinescens (L.Bolus) F.E.Weber. Synonyms: *Aptosimum abietinum* Burch. ex Benth., *Aptosimum scaberrimum* Schinz	Sandboega (A)	Scrophulariaceae	Herb	Leaves	Leaves are dried and used as a snuff for headache, stomachache and back ache	De Beer and Van Wyk (2011)	1
Arctotis laevis Thunb. Synonym: *Archotis laciniata*	Kankerbossie (KS)	Asteraceae	Herb	Leaves	Leaf used as compress for pains	Nortje and van Wyk (2015)	1
Artemisia absinthium L. Synonym: *Absinthium majus* Geoffr.	Wormwood, grand wormwood (E), Groenamara (KS)	Asteraceae	Herb	Leaves	Leaf used as compress for pain and backache. Leaves can be chewed or infused for the treatment of stomachache and menstruation pains	Nortje and van Wyk (2015); Hulley and Van Wyk (2019)	2
Artemisia afra Jacq. ex Willd. Synonyms: *Absinthium ponticum* (L.) Garsault, *Absinthium tenuifolium* Gaterau	Wild wormwood, African wormwood (E), Wildeals (KS), Mhlonyane (Z)	Asteraceae	Shrub	Leaves, stem	Leaves used as compress with cooking oil to alleviate pain (inflammation). For treating backache and stomach pain. Infusion used for headache and ear-ache. Leaves placed in ear as bud for toothache. An infusion made from a handful of the leaves can be taken daily to treat headache. Leaves are made into poultice for inflammation and rheumatism	Nortje and van Wyk (2015); De Beer and Van Wyk (2011); Coopoosamy and Naidoo (2012); Hulley and Van Wyk (2019); Thring and Weitz (2006); Mintsa Mi Nzue (2009)	6
Asclepias crispa P.J.Bergius. Synonyms: *Asclepias sabulosa*	Bitter Root (E), bitterhout (A), Witvergeet (KS)	Apocynaceae	Herb	Roots	The root used with a clove as snuff for headache, root chewed and placed in a tooth	Nortje and van Wyk (2015); Van Wyk et al. (2008);	3

Botanical name	*Common name	Family	Life-form	#Part used	Application	Reference	Nm
Schltr., *Asclepias sinuosa* Burm.f.					for toothache. Root decoctions used for toothache and stomachache. Root infusion is used as a wash for the treatment of rheumatism	Hulley and Van Wyk (2019)	
Asparagus exuvialis Burch.	Nkwangulatilo (Xitsonga)	Asparagaceae	Climber	Root	Root decoction is used for back pains	Tshikalange et al. (2016)	1
Asparagus laricinus Burch. Synonyms: *Asparagus angolensis* Baker, *Protasparagus laricinus* (Burch.) Oberm.	Bushveld Asparagus (E), Isgoba, Uvucu (Z)	Asparagaceae	Shrub	ns	Remedy for internal side pains in children	Mhlongo and Van Wyk (2019)	1
Athrixia phylicoides DC.	Bushman's tea (E), Boesmanstee (A), icholocholo, itshelo, Inkalane, Ishanelo, umtshanela (Z)	Asteraceae	Shrub	Leaves	Leaves are used with roots of *Athrixia elata* in decoctions for bathing sore feet. Musculo-skeletal (inflammation). For relieving headache	Watt and Breyer-Brandwijk (1962); Mhlongo and Van Wyk (2019); Mbanjwa (2020)	3
Azanza garckeana (F.Hoffm.) Exell & Hillc. Synonyms: *Bupariti garckeana* (F.Hoffm.) Rothm., *Thespesia garckeana* F.Hoffm.	Rhodesian tree-hibiscus, slime-apple, snot-apple, tree-hibiscus (E), slymappel, snotappel (A), morôbja, morôja, morôjwa (TW), mutogwe (V)	Malvaceae	Tree	Stem-bark	Stem bark is used to treat painful joints in aged individuals	Mongalo and Makhafola (2018)	1
Azima tetracantha Lam. Synonyms: *Azima angustifolia* DC., *Monetia barlerioides* L'Hér.	Beehanger, Bee-sting Bush, Four Thorns, Stink Bush, Needle Bush (E), Byangelbos, Naaldbos (A), Icegceya (X), Murunda (V), isiKhumukele, Gecaya (Z)	Salvadoraceae	Shrub	Roots	Roots are used for toothache. The sap is used for toothache	Gerstner (1941); Corrigan et al. (2011)	2
Baccharoides adoensis (Sch.Bip. ex Walp.) H.Rob. Synonyms: *Ascaricida adoensis*	innyathelo, inyathelo , uhlonyane (Z)	Asteraceae	Herb	Leaves, stem, roots	Decoctions from leaves and stem are used for stomach cramps, nervous spasms of	Watt and Breyer-Brandwijk (1962); Pujol	3

Botanical name	*Common name	Family	Life-form	#Part used	Application	Reference	Nm
Steetz, *Vernonia polymorpha* var. *polymorpha*					the stomach, and backbone pain. Root decoctions are taken for chest pain	(1990); Hutchings et al. (1996)	
Ballota africana (L.) Benth. Synonyms*: Marrubium africanum* L., *Stachys africana* (L.) Kuntze	Cape horehound, Cat Herb, Catmint (E), Kattekrui, Kattekruie, kattekruid (A), Kattekruid (KS)	Lamiaceae	Herb	Leaves	Leaves are used for the treatment of arthritis. As compress on sick children`s feet, to get rid of the pains, on head, for headache, on cheek for toothache. Leaf infusion is given for stomach pain and headache. For washing aching legs. Applied as an ointment to pain and inflammation as well as backache. Leaf infusion used externally for headache, rheumatism	Watt and Breyer-Brandwijk (1962); Nortje and van Wyk (2015); Van Wyk et al. (2008); De Beer and Van Wyk (2011); Hulley and Van Wyk (2019)	5
Bauhinia galpinii N.E.Br. Synonyms*: Bauhinia galpinii* var. *galpinii*, *Perlebia galpinii* (N.E.Br.) A.Schmitz	Pride of De Kaap (E), Vlam-van-die-Vlakte (A), Mutswiriri (V)	Fabaceae	Tree	Bark, roots	Maceration of the bark and root is drunk for stomach spasms	Arnold and Gulumian (1984)	1
Berchemia discolor (Klotzsch) Hemsl. Synonyms: *Phyllogeiton discolour, Scutia discolor*	Bird plum, wild almond (E), bruinivoor (A), umhlungulo, umumu, uvuku (Z)	Rhamnaceae	Tree	Bark	Bark is used for toothache	Hutchings et al. (1996)	1
Berchemia zeyheri (Sond.) Grubov. Synonyms: *Phyllogeiton zeyheri*, *Rhamnus zeyheri*	Ivory wood, red ebony (E), Dinee (SL), umgologolo, umncaka, umneyi, umnini (Z)	Rhamnaceae	Tree	Bark, roots	Bark infusions are administered as enemas for pains in the back and for rectal ulceration in children. Roots are used as remedy to relieve headache	Mabogo (1990); Watt and Breyer-Brandwijk (1962); Shai et al. (2020)	3
Berkheya bipinnatifida (Harv.) Roessler. Synonyms: *Stobaea*	Ubani (Z)	Asteraceae	Shrub	ns	Chest pain in children	Mhlongo and Van Wyk (2019)	1

Botanical name	*Common name	Family	Life-form	#Part used	Application	Reference	Nm
bipinnatifida Harv., *Stobaea seminivea* DC.							
Berkheya sp.	ikambi lomkuhlane, isidawo, ukakaka, ulimi-lwenkomo, ulimi-lwenyathi, ushaqa, ushwawu (Z)	Asteraceae	Shrub	Leaves, roots	Roots are mixed with pounded leaves in cold water and applied as foments for rheumatism. As a poultice to relieve aches and pains	Watt and Breyer-Brandwijk (1962); Pujol (1990)	2
Berkheya umbellata DC.	Geelklossiedissel (A), Ikhakhasi, Ulimi lwenkomo (Z)	Asteraceae	Shrub	ns	Musculo-skeletal (inflammation)	Mhlongo and Van Wyk (2019)	1
Berula erecta subsp. *thunbergii* (DC.) B.L.Burtt. Synonyms: *Berula thunbergii* (DC.) H.Wolff, *Berula erecta* (Huds.) Coville	Toothache Root, Water Parsnip (E)	Apiaceae	Herb	ns	Used for the treatment of toothache	Hulley and Van Wyk (2019)	1
Bidens pilosa L. Synonyms: *Bidens alausensis* Kunth, *Bidens cannabina* Lam.	Black jack, Spanish needles (E), knapsekerel, wewenaars (A), inongwe (X), amalenjane, uqadolo (Z)	Asteraceae	Herb	Stem, seeds, leaves	Young shoots are chewed for rheumatism. Burnt seed is rubbed into scarifications on the sides of the body for the relief of pain. Leaf decoction (1/4th a cup) is taken twice daily to treat arthritis. Squeezed liquid from leaves is used as ear drops to relief ear-ache. For relieving menstrual pains	Watt and Breyer-Brandwijk (1962); Hutchings et al. (1996); Bhat (2014); Mokganya and Tshisikhawe (2019); Mbanjwa (2020)	5
Blepharis capensis (L.f.) Pers. Synonyms: *Acanthodium capense* (L.f.) Nees, *Blepharis capensis* var. *latibracteata* Oberm.	scorpion’s tail (E), skerpioenstert (A), ubuHlungu besigcawu (X)	Acanthaceae	Shrub	ns	A poultice is applied onto teeth to treat toothache	Hulley and Van Wyk (2019)	1
Boophone disticha (L. f.) Herb. Synonyms: *Amaryllis disticha*	Cape poison bulb, sore-eye flower (E), gitbol, gifui, kopseerblom (A), incotho,	Amaryllidaceae	Herb	Bulbs	Bulb decoctions are administered by mouth or as enemas to adults for	Watt and Breyer-Brandwijk (1962); Hutchings	5

Botanical name	*Common name	Family	Life-form	#Part used	Application	Reference	Nm
L.f., *Boophone longipedicellata* Pax	incwadi, Ingcotho, Umayime (Z)				headaches, sharp chest pains and persistent bladder pains. Given to patients suffering from *inkwatshu*, a condition characterised by the development of cramp-like pains in the calf muscles associated with a feeling of tightness in the fingers and toes. Moistened bulb scales used for rheumatic pain. Analgesic (lower back aches). Bulb leaves used as compress for pain and inflammation	et al. (1996); Mhlongo and Van Wyk (2019); Coopoosamy and Naidoo (2012); Hulley and Van Wyk (2019)	
Boophone haemanthoides F.M.Leight.	Namaqua century plant (E), gifbol, kwaslelie (A)	Amaryllidaceae	Herb	Bulbs	Bulb used as a compress on paining knees	De Beer and Van Wyk (2011)	1
Boscia oleoides (Burch. ex DC.) Toelken. Synonyms: *Capparis clutiifolia* Burch. ex DC., *Capparis oleoides* Burch. ex DC.	Bastard Shepherd Tree, Karoo Shepherd Tree (E), Karoo-witgat, witgatboom (A), Umgqamagqama, Umgqomo-gqomo, Umphunzisa (X)	Capparaceae	Tree	Roots	Root is mixed with swartstorm root, bruised and drunk for stomach pain. Remedy for back pain	Van Wyk et al. (2008)	1
Bowiea volubilis Harv. = *Bowiea volubilis* Harv. Ex Hook.f. subsp. *volubilis*	Climbing onion (E), knolklimop (A), umgaqana (X), ugibisisila; iguleni (Z)	Asparagaceae	Herb	Bulbs	Used as remedy for headache and inflammation. Remedy for backache and muscle pain	Maroyi (2017); Philander (2011)	2
Brachylaena discolor DC. Synonyms: *Brachylaena discolor* subsp. *discolour*, *Brachylaena natalensis* Sch.Bip.	Coast silver oak (E), Kusvaalbos (A), Phahla (Z), Mphahla (NS), umPhahla (X)	Asteraceae	Tree	ns	Used for stomachache	Corrigan et al. (2011)	1
Brachylaena elliptica (Thunb.) Less. Synonyms: *Brachylaena dentata*; *Tarchonamhus ellipticus*	Bitter-leaf (E), bitterblaar, suurbos (A), iphahle, isiduli-ehlathi, uhlunguhlungu (Z)	Asteraceae	Tree	Leaves, roots	Leaf infusions are administered as enemas for backache. Decorticated root	Watt and Breyer-Brandwijk (1962); Hutchings et al. (1996)	2

Botanical name	*Common name	Family	Life-form	#Part used	Application	Reference	Nm
					infusions are taken as emetics for pains in the side		
Brackenridgea zanguebarica Oliv. Synonyms: *Brackenridgea bussei* Gilg, *Pleuroridgea zanguebarica* (Oliv.) Tiegh.	yellow peeling plane (E), Mutavhatsindi (V)	Ochnaceae	Tree	Roots	Powdered roots is rubbed on after treatment with *Aloe chabaudii* to relieve swollen ankles	Arnold and Gulumian (1984)	1
Bridelia cathartica Bertol. Synonyms: *Bridelia cathartica* subsp. *cathartica*, *Bridelia schlechteri* Hutch.	umKhawulangazi (Z)	Phyllanthaceae	Tree	Leaves	An infusion made from the leaves to soak feet to relieve pain	Corrigan et al. (2011)	1
Bridelia micrantha (Hochst.) Baill. Synonyms: *Bridelia stenocarpa* Müll.Arg., *Candelabria micrantha* Hochst.	Coastal goldenleaf (E), bruinstinkhout (A), Ditsere (SL), munzere (V) umhlamagwababa, umshonge (Z)	Phyllanthaceae	Tree	Roots, bark, leaves	Roots are used for severe epigastric pain and rubbed into the scalp for headache. Bark used for toothache and leaves for painful eyes and headache. Bark decoction is used to rinse the oral cavity to relieve toothache	Hutchings et al. (1996); Mabogo (1990); Shai et al. (2020), Arnold and Gulumian (1984)	4
Brunsvigia grandiflora Lindl. Synonyms: *Amaryllis banksiana* Lindl., *Brunsvigia sphaerocarpa* Baker	Giant Candelabra Flower (E), isichwe (X)	Amaryllidaceae	Herb	Leaves	Leaves are used externally during circumcision of boys to prevent inflammation	Bhat and Jacobs (1995)	1
Brunsvigia josephiniae (Delile) Ker Gawl. Synonyms: *Amaryllis gigantea* Marum, *Coburgia josephinae* (Delile) Herb.	Candelabra lily (E), kandelaarblom, lantanter (A)	Amaryllidaceae	Herb	ns	Used as a compress for pain and inflammatio	Hulley and Van Wyk (2019)	1
Buddleja saligna Willd. Synonyms: *Buddleja salicifolia* Jacq., *Nuxia saligna* (Willd.) Benth.	False olive (E), witolien (A), lelothwane (SS), mothlware (TW), unGqeba (X), iGqeba-elimhlope (Z)	Scrophulariaceae	Tree	Leaves	Leaf infusion used in treatment of teething problems	Hulley and Van Wyk (2019)	1
Bulbine abyssinica A.Rich. Synonyms: *Bulbine asphodeloides* var. *filifolioides*	Bushy bulbine (E), geelkatstert, wildekopieva (A), uyakayakana, intelezi (Xhosa); ibhucu (Zulu);	Xanthorrhoeaceae	Herb	Leaves, roots	For relieving menstrual pain. Root is used for back pain	Maroyi (2017); Van Wyk et al. (2008)	2

Botanical name	*Common name	Family	Life-form	#Part used	Application	Reference	Nm
De Wild., *Bulbine xanthobotrys* Engl. & Gilg							
Bulbine alooides (L.) Willd. Synonyms: *Bulbine acaulis* L., *Anthericum alooides* L.	Kopiva (A), Waterpypie, Wildekopiva (A), Ibhucu (Z)	Xanthorrhoeaceae	Herb	Tubers	Tubers were used for rheumatism by the Xhosa and by Dutch settlers in the Cape	Watt and Breyer-Brandwijk (1962); Hutchings et al. (1996)	2
Bulbine frutescens (L.) Willd.	Stalked bulbine, snake flower (E), Geneesbos(sie) (KS)	Xanthorrhoeaceae	Shrub	Leaves	Fleshy part of leaf and leaf juice used for pains and sprained ankles	Nortje and van Wyk (2015)	1
Bulbine latifolia (L.f.) Spreng. Synonyms: *Bulbine brunsvigiaefolia, Bulbine natalensis*	Broad-leaved bulbine, red carrot (E), rooiwortel, geelkopieva (A), incelwane (X), ibhucu (Z)	Xanthorrhoeaceae	Herb	Tubers, roots	Xhosa and Dutch settlers use tubers for rheumatism. Root infusions or decoctions are used for rheumatism. Roots are used for treating arthritis	Hutchings et al. (1996); Van Wyk and Gericke (2000); Philander (2011)	3
Cadaba aphylla (Thunb.) Wild. Synonyms: *Cadaba juncea* Szyszył., *Cleome sodiroi* Gilg ex Heilbr.	leafless cadaba, leafless wormbush (E), swartstorm, doubos, douwurmbos, gifhoutjie, rooistorm, stormbos (A), mfitshwana, mhitshwana, monnamontsho (TW), mudiatsiwana, tshikuni (V), usitorhom (X)	Capparaceae	Shrub	Roots	Root infusion used for treating backache, constipation, stomachache, rheumatism, menstruation pains, general pain and inflammation	Hulley and Van Wyk (2019)	1
Caesalpinia decapetala (Roth.) Alston. Synonym: *Biancaea decapetela* (Roth) O. Deg.	Mauritius thorn (E), Luanakha (V)	Fabaceae	Shrub	Roots	Roots are boiled and the mixture drank for treating dysmenorrhoe	Mahwasane et al. (2013)	1
Canna indica L. Synonyms: *Canna achiras* Gillies ex D.Don, *Xyphostylis lutea* (Mill.) Raf.	African arrowroot, edible canna, purple arrowroot, Sierra Leone arrowroot (E), kanna, kennablare (A)	Cannaceae	Herb	Leaves	Leaves used as compress for pain and inflammation, backache, rheumatism, sores, headache, toothache and sprains	Hulley and Van Wyk (2019)	1
Cannabis sativa L. Synonyms: *Cannabis chinensis* Delile,	Marijuana (E), dagga (A), Umya, Matakwane, intsangu (X),	Cannabaceae	Herb	Whole plant, leaves	Whole plant is used to treat excessive headache. Leaf decoction is taken to relieve	Mongalo and Makhafola (2018); Bhat	3

Botanical name	*Common name	Family	Life-form	#Part used	Application	Reference	Nm
Cannabis sativa var. *indica* (Lam.) Wehmer	Matekwane/Patse (NS), Nsangu (Z)				chronic pain. Used as painkillers and for toothache	(2013); Mbanjwa (2020)	
Canthium spinosum (Klotzsch ex Eckl. & Zeyh.) Kuntze. Synonym: *Plectronia spinosa* Klotzsch	Thorny turkey-berry, coastal turkey-berry, coastal canthium (E), Ikhanyisani, Isihlungu sankonka, Ubuchopho bekati, Ubuchopho benja, Udlozini, Umhlabandlazi (Z)	Rubiaceae	Shrub	ns	Analgesic (sharp internal body pains)	Mhlongo and Van Wyk (2019)	1
Capparis tomentosa Lam. Synonyms: *Capparis alexandrae* Chiov., *Capparis subtomentosa* De Wild.	Woolly caper bush (E), Wollerige kapperbos (A), inkunzi-ebomvu, iqwaningi, umqoqolo (Z)	Capparaceae	Tree	Roots	The roots boiled in water half a cupful of the infusion is taken three times a day. Powdered roots are rubbed on swollen ankles. Roots are burned and the smoke is inhaled to relieve headache	Hutchings et al. (1996); Pujol (1990); Watt and Breyer-Brandwijk (1962); Arnold and Gulumian (1984)	4
Carissa bispinosa (L.) Desf. ex Brenan	Forest num-num (E), bosnoemnoem (A), Amathungulu (Z)	Apocynaceae	Shrub	ns	Analgesic (headache)	Mhlongo and Van Wyk (2019)	1
Carissa macrocarpa (Eckl.) A.DC. Synonyms: *Carissa africana* A.DC., *Jasminonerium africanum* (A.DC.) Kuntze	Natal plum, big num-num (E), Amathungulu (Z)	Apocynaceae	Shrub	ns	Analgesic (headache)	Mhlongo and Van Wyk (2019)	1
Carissa spinarum L. Synonyms: *Antura edulis* Forssk., *Carissa edulis* (Forssk.) Vahl.	Simple-spined num-num, climbing num-num, small num-num (E), kleinnoemnoem (A), mothokolo (NS), Dithokolo (SL), murungulu (V)	Apocynaceae	Shrub	Roots	Roots are medicine used for body pains	Shai et al. (2020)	1
*Carpobrotus dimidiatus (*Haw.) L. Bolus. Synonyms: *Carpobrotus juritzii* (L.Bolus)	Natal sour fig (E), Natalse suurvy/strandvy (A), Ibohlololo lesilungu (Z)	Aizoaceae	Creeper	ns	Musculo-skeletal inflammation. Used as poultice for painful and swollen feet	Mhlongo and Van Wyk (2019); Mbanjwa (2020)	2

Botanical name	*Common name	Family	Life-form	#Part used	Application	Reference	Nm
L.Bolus, *Mesembryanthemum juritzii* L.Bolus							
Carpobrotus edulis (L.) N.E.Br. Synonyms: *Abryanthemum edule* (L.) Rothm., *Mesembryanthemum edule* L.	Sour fig, Cape fig, Hottentots fig (E), ghaukum, ghoenavy, Hottentotsvy, Kaapsevy, perdevy, rankvy, suurvy, vyerank (A), ikhambi-lamabulawo, umgongozi (Z)	Aizoaceae	Creeper	Leaves	To treat painful lungs. Leaf juice gargled for sore throat, teething problems and ear-ache as well as stomachache	Van Wyk et al. (2008); Hulley and Van Wyk (2019); Philander (2011); Mogale et al. (2019)	4
Cassine transvaalensis (Burtt Davy) Codd. Synonyms: *Elaeodendron transvaalense* (Burtt Davy) R.H.Archer, *Salacia transvaalensis* Burtt Davy	Mulumana-mana, mukuvhazwihi (V)	Celastraceae	Tree	Bark, roots	Bark infusion is drunk twice daily for stomachache. maceration of the powdered roots is drunk to relieve stomachache in male	Arnold and Gulumian (1984)	1
Cassytha ciliolata Nees. Synonyms: *Cassytha capensis* Meisn., *Cassytha triflora* E.Mey.	Devil's Tresses, False Dodder (E), Bôjaantou (KS)	Lauraceae	Climber	Leaves	Leaf decoction is used for pains, snuff for headache	Nortje and van Wyk (2015)	1
Catharanthus roseus (L.) G.Don. Synonyms: *Lochnera rosea*, *Pervinca rosea* (L.) Gaterau, *Vinca rosea* var. *alba* (G. Don) Sweet	Periwinkle, vinca (E), Imbali yamathuna, Imbali yesibaya, Isona, Ubani bezwe, Umangashi (Z)	Apocynaceae	Herb	Leaves, roots, milky sap	Leaves are used for the rheumatism. Milky sap is used for insect bites. Roots are used for toothache. Analgesic (headache; toothache)	Watt and Breyer-Brandwijk (1962); Hutchings et al. (1996); Mhlongo and Van Wyk (2019); Mogale et al. (2019)	4
Cavacoa aurea (Cavaco) J. Léonard. Synonym: *Grossera aurea* Cavaco	Natal hickory (E), Natalokkerneut (A), umbhuku, umbuku (Z)	Euphorbiaceae	Tree	Roots	Root infusions are taken to ease pain	Palmer and Pitman (1961)	1
Celtis africana Burm.f. Synonyms: *Celtis burmanni*	white stinkwood (E), witstinkhout (A), Modutu (TW), umVumvu (X),	Cannabaceae	Tree	Bark	Bark decoction is used as nose and ear drops to relieve	Arnold and Gulumian (1984)	1

Botanical name	*Common name	Family	Life-form	#Part used	Application	Reference	Nm
Planch., *Celtis vesiculosa* Hochst. ex Planch.	Mumvumvu, Mpopano (V), uSinga lwesalukazi (Z)				toothache due to mandibular and maxillar pains		
Cenchrus ciliaris L. Synonyms: *Cenchrus bulbosus* Fresen., *Cenchrus rufescens* Desf.	Buffalo grass (E),bloubuffel sgras (A), idungamuzi, indungulu (Z)	Poaceae	Herb	Stem/runne r	Underground runners are used for body pain and dysmenorrhoea (painful menstruation)	Pujol (1990); Gebashe et al. (2019)	2
Centaurea benedicta (L.) L. Synonyms: *Benedicta officinalis* Bernh., *Cnicus benedictus* L. *Epitrachys microcephala* K.Koch	St. Benedict's thistle, blessed thistle, holy thistle, spotted thistle (E), karmedik, doringbietoea (A)	Asteraceae	Herb	Leaves, stem, ns	Infusions used for the treatment of stomachache	Hulley and Van Wyk (2019); Mintsa Mi Nzue (2009)	2
Centella asiatica (L.) Urb. Synonyms: *Centella hirtella* Nannf., *Hydrocotyle biflora* P. Vell.	Marsh Pennywort (E), kleinkattekruid, varkoortjies (A), Umangobozane, Isgoba (Z)	Apiaceae	Herb	Whole plant, leaves	Fresh plant decoction is taken orally for rheumatoid arthritis. Analgesic (sharp internal body pains). Fresh leaves used as ear plugs to relief ear pain in children	Van Wyk and Gericke (2000); Mhlongo and Van Wyk (2019); Van Wyk et al. (2008)	3
Centella dolichocarpa M.Schub. & B.-E.van Wyk	Skillpadbossie (A)	Apiaceae	Herb	Leaves	Used for arthritis	Philander (2011)	1
Cephalaria zeyheriana Szabó. Synonym: *Cephalaria ustulata*	False scabiosa (E), Uzondle (Z)	Caprifoliaceae	Herb	Roots	Root decoctions are for any unaccountable swellings and pains	Watt and Breyer-Brandwijk (1962)	1
Chamarea capensis (Thunb.) Eckl. & Zeyh. Synonyms: *Anethum capense* Thunb., *Foeniculum capense* DC.	Cape Caraway, Chamare (E), Finkelwortel, Vinkelbol (A)	Apiaceae	Herb	Roots	Root is chewed to treat stomachache	Hulley and Van Wyk (2019)	1
Chamarea longipedicellata B.L.Burtt	Vinkel (A)	Apiaceae	Herb	Roots	Roots is used to relieve headache	De Beer and Van Wyk (2011)	1
Chironia baccifera L. Synonyms: *Chironia parviflora* Salisb., *Chironia baccifera* var. *elongata* E.Mey.	Christmas berry (E), aambeibossie, bitterbossie (A), Bitterbos (KS)	Gentianaceae	Shrub	Leaves, whole plant	Used for backache, rheumatism, arthritis and woman ailments (menstrual pains). Infusions used for stomach ailments, pain and	Nortje and van Wyk (2015); Hulley and Van Wyk (2019); Mintsa Mi Nzue (2009)	3

Botanical name	*Common name	Family	Life-form	#Part used	Application	Reference	Nm
					inflammation, backache and headache		
Chrysocoma ciliata L. Synonyms: *Aster discoideus* Sond., *Chrysocoma tenuifolia* P.J.Bergius	Bitter bush, bitter cowcud (E), beesbos, bitterbos (A)	Asteraceae	Shrub	Leaves, twigs	Leaf decoction is used for aching legs. Chew twig for stomach ailments and ulcers, leaves used as bud for toothache	De Beer and Van Wyk (2011); Hulley and Van Wyk (2019)	2
Cichorium intybus L. Synonyms: *Cichorium balearicum* Porta, *Cichorium intybus* f. *rubicunda* Farw.	Blue dandelion, chicory (E), sigorei, bloublommetjie (A)	Asteraceae	Herb	ns	Used for the treatment of stomach ailments and rheumatism	Hulley and Van Wyk (2019)	1
Cinnamomum camphora (L.) J.Presl. Synonyms: *Camphora officinarum* var. *glaucescens* A.Braun, *Cinnamomum officinarum* Nees ex Steud.	Camphor (E), Urosalina (Z)	Lauraceae	Tree	Bark	Used for inflammation	Philander (2011)	1
Cissampelos capensis L.f. Synonyms: *Antizoma capensis* (L.f.) Diels, *Phyllanthus cinereoviridis* Pax	Davidjieswortel, Dawidjieswortel, Fynblaarklimop (A)	Menispermaceae	Shrub	Roots, leaves	Roots are chewed for severe stomach pain. Root infusions as remedy for toothache and headache. Fresh leaf infusions and decoctions are used for treating pain, backache and stomach ailments	Van Wyk et al. (2008); Hulley and Van Wyk (2019); Mintsa Mi Nzue (2009)	3
Cissampelos torulosa E.Mey. ex Harv. & Sond. Synonyms: *Cissampelos truncatus* Engl., *Menispermum capense* Thunb.	Davidjies, Davidjieswortel (A), Ukhalimele, Ukhalimele-omkhulu, Umthombo (Z)	Menispermaceae	Shrub	ns	Used for backache	Hulley and Van Wyk (2019)	1
Clausena anisata (Willd.) Hook.f. ex Benth. Synonyms: *Clausena inaequalis*, *Clausena inaequalis*	Horsewood (E), basternieshout (A), isifuthu, umnukelambiba, umsanka (Z)	Rutaceae	Shrub	Leaves, bark, roots	Leaf decoctions are used for rheumatism and fevers. Leaf decoctions are taken or inhaled for abdominal pain and toothache	Hutchings et al. (1996); Philander (2011)	2

Botanical name	*Common name	Family	Life-form	#Part used	Application	Reference	Nm
Clematis brachiata Thunb. Synonyms: *Clematis biloba* Steud., *Clematis triloba* var. *congensis* (A.Chev.) M.Johnson	traveller's joy, old man's beard, wild clematis (E), klimop, lemoenbloeisels (A), ityolo (X), Tshiumbeumbe (V), umdlonzo (Z)	Ranunculaceae	Climber	Leaves	Fresh leaves are smoke and inhaled to relieve headache. Leaf decoction at 1 teasppon is drunk thrice daily and team is inhaled to relieve headache	Arnold and Gulumian (1984)	1
Cliffortia odorata L.f. Synonyms: *Cliffortia alnifolia* Rchb. *Cliffortia odorata* var. *vera* Harv.	Wild vine (E), wildewingerd, wildevyerank (A)	Rosaceae	Shrub	Leaves, roots, stem, ns	Used for backache, pain and inflammation as well as arthritis	Hulley and Van Wyk (2019); Philander (2011); Mintsa Mi Nzue (2009)	3
Colocasia antiquorum Schott. Synonyms: *Colocasia fontanesii* Schott, *Colocasia tonoimo* Nakai	Elephant's ear (E), idumbe (lomfula), idumbi (Z)	Araceae	Herb	Tubers, roots, leaves	Crushed root decoctions are administered as enemas for stomach trouble. Tubers are used as poultices for rheumatism. Bruised leaves are applied directly to cuts from insect stings	Hutchings et al. (1996); Hulme (1954); Pujol (1990)	3
Colophospermum mopane (Benth.) Leonard. Synonym: *Copaifera mopane* Benth.	mopane, turpentine tree (E), mopanie (A), mohlanare (NS); mophane (TW)	Fabaceae	Tree	Leaves	Leaf decoction is drunk for stomachache	Arnold and Gulumian (1984)	1
Combretum caffrum (Eckl. & Zeyh.) Kuntze. Synonyms: *Combretum salicifolium* E.Mey. ex Hook., *Dodonaea dubia* Eckl. & Zeyh.	Bush willow bushveld willow (E), isidubu (X)	Combretaceae	Tree	Roots	Ground root is boiled and filtered and 1 cup of the mixture is mixed with water to bathe (twice daily) to relieve body and leg pains	Bhat and Jacobs (1995)	1
Combretum collinum Fresen.	Weeping Bushwillow, Bicoloured Bushwillow (E), Vaiërende Boswilg (A), Muvuvha (V), Fufu (Xitsonga)	Combretaceae	Tree	Roots	Root infusion is used for painful legs, cramps and joint pains	Tshikalange et al. (2016)	1
Combretum kraussii Hochst. Synonyms: *Combretum nelsonii*	umDubu wehlathi, umDubu (X)	Combretaceae	Tree	Roots	Ground root is boiled and filtered and 1 cup of the mixture is mixed with water	Bhat and Jacobs (1995); Mbanjwa (2020)	2

Botanical name	*Common name	Family	Life-form	#Part used	Application	Reference	Nm
Dummer, *Combretum woodii* Dummer					to bathe (twice daily) to relieve body and leg pains. Rememdy for back pain		
Combretum molle R.Br. ex G.Don. Synonyms: *Combretum gueinzii* Sond, *Combretum velutinum* DC.	Velvet bush willow, velvet leaf willow (E), mokgwethe (NS), Isibondwe, Umbonda, Umbondwe (Z)	Combretaceae	Tree	ns	Analgesic (general body pains, internal sharp body pains, lower back pain)	Mhlongo and Van Wyk (2019)	1
Combretum paniculatum Vent. Synonyms: *Combretum abbreviatum* Engl., *Combretum ramosissimum* Engl. & Diels	Burning bush, forest flame-creeper (E), Mukopo-kopo, Gopo-Gopo (V)	Combretaceae	Shrub	Roots	Root decoction is used to prepare soft porridge twice daily and taken to relieve headache die to indigestion	Arnold and Gulumian (1984)	1
Commelina africana L. Synonyms: *Commelina edulis* A.Rich, *Dirtea africana* (L.) Raf.	Common yellow commelina (E), Geeleendagsblom (A), Idangabane, Idangabane lomfula (Z)	Commelinaceae	Herb	ns	Musculo-skeletal (inflammation). Used for menstruation pains	Mhlongo and Van Wyk (2019); Hulley and Van Wyk (2019)	2
Commelina benghalensis L. Synonyms: *Commelina canescens* Vahl, *Commelina turbinata* Vahl	Benghal dayflower, tropical spiderwort, Benghal wandering Jew (E), blouselblommetjie (A), Idangabane lentaba (Z)	Commelinaceae	Herb	ns	Musculo-skeletal (inflammation)	Mhlongo and Van Wyk (2019)	1
Commelina eckloniana Kunth. Synonyms: *Commelina weimarckiana* Norl, *Polyspatha eckloniana* Hassk. ex C.B.Clarke.	Idangabane, Idangabane lamanzi (Z)	Commelinaceae	Herb	ns	Musculo-skeletal (inflammation)	Mhlongo and Van Wyk (2019)	1
Commelina erecta L. Synonyms: *Commelina aethiopica* C.B.Clarke, *Commelina elegans* Kunth	White mouth dayflower, slender dayflower, or widow's tears (E), Idangabane (Z)	Commelinaceae	Herb	ns	Musculo-skeletal (inflammation)	Mhlongo and Van Wyk (2019)	1
Convolvulus sagittatus Thunb.	Wild Bindweed (E), bobbejaantou (A), Uboqo (X), Uvimbukhalo (Z)	Convolvulaceae	Herb	Roots	Headache	Maroyi (2017)	1

Botanical name	*Common name	Family	Life-form	#Part used	Application	Reference	Nm
Conyza scabrida DC. Synonyms: *Nidorella ivifolia* (L.) J.C.Manning & Goldblatt, *Erigeron dentatus* Burm.f, *Fimbrillaria baccharoides* Cass.	Oven Bush (E), Bakbos, oondbos, paddabos (A), Vleiwilger (KS)	Asteraceae	Shrub	Leaves	Leaf decoction used for backache. Infusion with ballerja used for headache. Leaf infusions used for cramps after labour and pain as well as rheumatism. Used as a compress to relieve, arthritis, pain and inflammation, headache, backache and stomachache	Nortje and van Wyk (2015); Van Wyk et al. (2008); De Beer and Van Wyk (2011); Hulley and Van Wyk (2019); Philander (2011); Thring and Weitz (2006)	6
Cotyledon orbiculata L. Synonyms: *Cotyledon ambigua* Salisb., *Cotyledon tricuspidata* Haw.	Pig's ears, cotyledon (E), plakkie, platjies, varkoorblare, varkoor, kouterie (A), imphewula (X), ipewula (Z)	Crassulaceae	Herb	Leaves	Leaves are boiled, filtered and a drop of decoction is used for ear-ache. Leaves is heated and placed on a swollen body part to treat inflammation. Leaf juice treats earache, toothache. Used for ear-ache	Bhat and Jacobs (1995); Hulley and Van Wyk (2019); Philander (2011)	3
Crassula muscosa L. Synonyms: *Combesia muscosa* (L.) P.V.Heath, *Crassula imbricata* Burm.f.	skilpadbos; klein koorsbos (A)	Crassulaceae	Herb	Leaves, stem	Leafy stem use to treat back ache	De Beer and Van Wyk (2011)	1
Crinum bulbispermum (Burm.f.) Milne-Redh. & Schweick. Synonyms: *Crinum capense* Herb, *Amaryllis riparia* Burch. ex Kunth	Orange River lily, Vaal River lily, wild amaryllis (E), Oranjerivierlelie (A); umnduze (Z)	Amaryllidaceae	Herb	Bulbs	Roasted bulbs are applied to aching joints, rheumatism, varicose veins and backache	Watt and Breyer-Brandwijk (1962)	1
Crinum macowanii Baker. Synonyms: *Crinum corradii* Chiov. ex Chiarugi, *Crinum pedicellatum* Pax	Boslelie (A), Cape Coast Lily, Common Vlei Crinum (E), Intelezi (X), Intelezi, Uguqu, Umduze (Z)	Amaryllidaceae	Herb	Bulbs	Decoctions of the bulb taken orally for rheumatic fever. Analgesic (toothache), musculo-skeletal inflammation. Used for headache	Hutchings et al. (1996); Van Wyk and Gericke (2000); Mhlongo and Van Wyk (2019); Mbanjwa (2020)	4

Botanical name	*Common name	Family	Life-form	#Part used	Application	Reference	Nm
Croton gratissimus Burch. Synonyms: *Croton gratissimus, Croton antunesii* Pax	Bergboegoe (A), lavender croton, fever-berry (E), Mufhorola (V) Uhubeshane (Z)	Euphorbiaceae	Shrub	Bark, leaves	Finely ground dry bark is rubbed as an irritant into incisions on the skin for inflammation and pain in the chest. Leaves as ingredients in mixtures used for 'smoking' by rheumatic patients	Watt and Breyer-Brandwijk (1962); Hutchings et al. (1996)	2
Croton steenkampianus Gerstner	Marsh Fever-berry (E), vleikoorsbessie (A), uhubeshane omkhulu (Z)	Euphorbiaceae	Shrub	Leaves	Steam from fresh leaf decoctions is inhaled to relieve aches. A remedy against painful joints, back and rheumatism	Pooley (1993); Watt and Breyer-Brandwijk (1962); Hutchings et al. (1996)	3
Croton sylvaticus Hochst. Synonyms: *Croton bukobensis* Pax, *Croton verdickii* De Wild.	Boskoorsbessie (A), forest croton, forest fever-berry (E), uminya, ummbila, Umgweba, Umzilanyoni (Z)	Euphorbiaceae	Shrub	Bark	Bark is used for abdominal disorders, internal inflammations. Analgesic (toothache, sharp internal body pains)	Bryant (1966); Mhlongo and Van Wyk (2019)	2
Cryptocarya latifolia Sond.	Broad-leafed quince, Nitonga nut (E), Basterswartysterhout (A), umkhondweni, undlangwenya (Z)	Lauraceae	Tree	Bark	Bark is also used to treat internal pains, muscular and cramps	Pujol (1990)	1
Cryptocarya myrtifolia Stapf. Synonyms: *Cryptocarya vacciniifolia* Stapf, *Cryptocarya vaccinioides* Kosterm.	Camphor tree (E), kanferboom (A), igqeba, umkhondweni, umngqabe (Z)	Lauraceae	Tree	Bark	Snuff or smoke from the bark is inhaled to treat headaches.	Hutchings et al. (1996); Watt and Breyer-Brandwijk (1962)	2
Cucumis africanus L.f. Synonyms: *Cucumis hookeri* Naud, *Cucumis arenarius* Schrad.	Agurkie, bitterappel (A), thorn cucumber, wild cucumber (E), isende-lenja, uselwa-lwemamba (Z)	Cucurbitaceae	Herb	Fruit, leaves	Warm water infusions of fruit pulp are administered as enemas for the relief of lumbago	Watt and Breyer-Brandwijk (1962)	1
Cussonia nicholsonii Strey	South Coast Cabbage-tree (E), Insengane, Umsenge (Z)	Araliaceae	Tree	ns	Musculo-skeletal inflammation	Mhlongo and Van Wyk (2019)	1

Botanical name	*Common name	Family	Life-form	#Part used	Application	Reference	Nm
Cussonia spicata Thunb. Synonyms: *Cussonia boivinii* Drake, *Cussonia triptera* Colla	Cabbage-tree, common cabbage tree (E), kiepersol (A), Umsenge (Z)	Araliaceae	Tree	ns	Musculo-skeletal inflammation. Used for headache	Mhlongo and Van Wyk (2019); Mbanjwa (2020)	2
Cyanotis speciosa (L.f.) Hassk. Synonyms: *Tradescantia nodiflora* Lam, *Tonningia nodiflora* (Lam.) Kuntze	Doll's Powderpuff, Job's Tears (E), loupoeierkwassie (A), Theepe-balingoana (SS), Iphindemuva, (Udabulamafu), Unkungwini (Z)	Commelinaceae	Herb	ns	Musculo-skeletal (inflammation)	Mhlongo and Van Wyk (2019)	1
Cycnium racemosum Benth. Synonyms: *Cycnium huttoniae*, *Cycnium kraussianum*	Large mountain ink flower (E), berginkplant (A), Injanga (X), Uhlabahlangane (Z)	Orobanchaceae	Herb	Roots	Root infusions are taken as emetics for general pains in the body	Watt and Breyer-Brandwijk (1962)	1
Cymbopogon marginatus (Steud.) Stapf ex Burtt-Davy. Synonyms: *Andropogon marginatus* Steud., *Andropogon nardus* subsp. *marginatus* (Steud.) Hack.	Lemon Grass, Lemon-scented Grass, Dobo Grass, Khuskhus (E), Koperdraad, Koperdraadgras, Kuskusgras (A), Lebata (SS), Fungwi (V), Umqungu (X), Isiqunga (Z)	Poaceae	Grass	Roots	Roots are burned and smoke inhaled to relieve headache	Arnold and Gulumian (1984)	1
Cyperus papyrus L. Synonyms: *Chlorocyperus papyrus* (L.) Rikli, *Papyrus antiquorum* Willd.	Papyrus (E), Papirus (A), Ibhuma, Intandelo (Z)	Cyperaceae	Herb	ns	Musculo-skeletal (inflammation)	Mhlongo and Van Wyk (2019)	1
Cyphostemma cirrhosum (Thunb.) Desc. ex Wild & R.B. Synonyms: *Cissus cirrhosa* (Thunb.) Willd, *Vitis cirrhosa* Thunb.	Wildedruif, Wildedruiwe (A), Isidikili (Z)	Vitaceae	Creeper	ns	Musculo-skeletal (inflammation)	Mhlongo and Van Wyk (2019)	1
Cyrtanthus obliquus (L.f.) Aiton. Synonyms: *Amaryllis pendula* Salisb., *Timmia obliqua* (L.f.) J.F.Gmel.	Knysna lily (E), Knysnalelie (A), Umathunga (X), Umathaga (Z)	Amaryllidaceae	Herb	Roots	Root infusion is taken for stomachache	Bhat and Jacobs (1995)	1

Botanical name	*Common name	Family	Life-form	#Part used	Application	Reference	Nm
Dais cotinifolia L. Synonyms: *Dais canescens* Bartl. ex Meisn., *Lasiosiphon grandifolius* Gilli	Pompon tree, pincushion tree (E), kannabas, speldekussing, basboom (A), intozani, isihlungu (X), intozwane-emnyama (Z)	Thymelaeaceae	Tree	Leaves	Leaf decoction (half a cup) is used for treating stomachache	Bhat and Jacobs (1995)	1
Dasispermum suffruticosum (P.J.Bergius) B.L.Burtt. Synonyms: *Capnophyllum jacquinii* DC., *Conium suffruticosum* P.J.Bergius	Wild parsley, Sea Parsley, Duineseldery (E)	Apiaceae	Herb	Leaves	Leaves are used for rheumatism	Philander (2011)	1
Dalbergia armata E.Mey.	Thorny-rope, flat-bean, Hluhluwe climber (E), doringtou (A), sehlokootswa (NS), uBobo (X), Umhluhluwe, Umhluhlube (Z)	Fabaceae	Shrub	Roots, leaves, ns	Analgesic (sharp internal body pains). The roots are boiled in water and the water is gargled to relieve toothache. Leaf decoction is taken (half a cup thrice daily) for body pains	Mhlongo and Van Wyk (2019); Corrigan et al. (2011); Bhat and Jacobs (1995)	3
Dalbergia obovata E.Mey. Synonym: *Dalbergia sessiliflora* Harms	climbing flat bean (E), bobbejaankoudoring, rankplatboontjie (A), Umzungulu (Z)	Fabaceae	Climber	ns	For treating ear-ache and muscular cramps	Mhlongo and Van Wyk (2019); Mbanjwa (2020)	2
Datura stramonium L. Synonyms: *Datura bernhardii*, *Datura inermis*	Downy thorn apple, ditch weed, Jimson weed, stinkwort (E), gewone stinkblaar, malpitte (A), Stinkblaar (KS), umhlavuthwa (X), iloqi (Z)	Solanaceae	Herb	Leaves	Leaves are smoked for the relief of headaches. Leaf infusions are used for treating rheumatism. Powdered leaves are applied to human and animal bruises to alleviate inflammation. Leaves applied as compress on pain (inflammation), backache, headache and ear-ache	Van Wyk et al. (1997); Watt and Breyer-Brandwijk (1962); Nortje and van Wyk (2015); Hulley and Van Wyk (2019)	4

Botanical name	*Common name	Family	Life-form	#Part used	Application	Reference	Nm
Desmodium incanum DC. Synonyms: *Desmodium sparsiflorum* G. Don, *Meibomia racemifera* (DC.) Kuntze	Creeping beggerweed (E), isinama, Umboniselwa, Inamathela (Z)	Fabaceae	Shrub	ns	For treating back pain	Mbanjwa (2020)	1
Dianthus thunbergii S.S.Hooper. Synonym: *Dianthus scaber* Thunb.	Wild Pink (E), aandblommetjie, Grashout, Wilde-angelier (A)	Caryophyllaceae	Herb	ns	Used to treat stomachache	Hulley and Van Wyk (2019)	1
Dicerocaryum eriocarpum (Decne.) Abels	Devil's thorn (E), beesdubbeltjie, seepbos (A), makanagwe (TW)	Pedaliaceae	Herb	Whole plant	Whole plant is burnt for treating headache	Tshikalange et al. (2016)	1
Dicerothamnus rhinocerotis (L.f.) Koek. Synonyms: *Elytropappus rhinocerotis* (L.f.) Less., *Stoebe rhinocerotis* L.f., *Seriphium adpressum* DC.	Rhinoceros bush, rhenoster bush (E), renosterbos, rhenosterbos (A) Renosterbos (KS)	Asteraceae	Shrub	Leaves, stem	Leaf decoction used for painful legs and as wash for burning feet, as compress for backache. Used as wash for rheumatism. Infusion of young stem is used for back pain. Leaves are chewed and juices swallowed for stomachache. Infusions used for stomachache, headache and earache	Nortje and van Wyk (2015); Van Wyk et al. (2008); Hulley and Van Wyk (2019); Thring and Weitz (2006)	4
Dichrostachys cinerea (L.) Wight & & Arn. Synonyms: *Acacia engleri*; *Acacia spinosa*, *Cailliea dichrostachys*	Sickle bush (E), Sekelbos (A), ugagane, ugegane, umthezane, Udonsuthando, umzilazembe, usegwane (Z)	Fabaceae	Shrub	Roots	Roots are an ingredient in a decoction taken to ease pain or rubbed into incisions cut over the painful area. Analgesic (sharp internal body pains), side pains in babies	Palmer and Pitman (1961); Mhlongo and Van Wyk (2019)	2
Dicoma capensis Less. Synonyms: *Berkheya albida* DC., *Tibestina lanuginosa* Maire	Fever bush (E), Karmedik, verpis, vyfpondbos, Melktou (A), koorsbos(sie) (KS)	Asteraceae	Herb	Leaves	Leaf infusion with other plants used for rheumatism and backache. For treating rheumatism and stomach pain	Nortje and van Wyk (2015); Van Wyk et al. (2008); De Beer and Van Wyk (2011)	3

Botanical name	*Common name	Family	Life-form	#Part used	Application	Reference	Nm
Dioscorea dregeana (Kunth) T. Durand & Schinz Synonyms: *Helmia dregeana* Kunth, *Dioscorea dregeana* var. *dregeana*	Wild yam (E), wildejam (A), Ingevu, Intana ebovu, Udakwa, Ufudu, Ufudulwehlathi (Z)	Dioscoreaceae	Creeper	ns	Analgesic (sharp internal body pains)	Mhlongo and Van Wyk (2019)	1
Diosma oppositifolia L. Synonyms: *Diosma decussata* Lam., *Diosma succulenta* P.J.Bergius	Bitter buchu (E), bitterboegoe (A)	Rutaceae	Shrub	Leaves	Leaves used external for sprains	Philander (2011)	1
Diosma prama I.Williams	Little Karoo buchu (E)	Rutaceae	Shrub	ns	Used to treat backache	Hulley and Van Wyk (2019)	1
Diospyros austro-africana De Winter	Fire-sticks, star-apple (E), jakkalsbos, vuurmaakbossie, kraaibessie (A), liperekisi-tsa-makhoaba, senokonoko (SS), umbhongisa (X)	Ebenaceae	Shrub	Roots	Dried roots are smoked to relieve headache	Van Wyk et al. (2008)	1
Diospyros mespiliformis Hochst. ex A.DC. Synonyms: *Diospyros bicolor* Klotzsch, *Diospyros senegalensis* Perrier ex A.DC.	African ebony, jackal-berry (E), jakkalsbessie (A), Musuma (V)	Ebenaceae	Tree	Seeds	Seed decoction is drunk or used to prepare soft porridge for headache	Arnold and Gulumian (1984)	1
Diospyros villosa (L.) De Winter var. *villosa*. Synonyms: *Royena corilyfolia*, *Royena scabra*, *Royena scandens*	Hairy star-apple (E), harige sterappel, bloubos (A), umbishimbishi, umbongisa, Umqandane wesempisi, Indodemnyama (Z)	Ebenaceae	Shrub	Leaves, roots	Leaves or pounded roots are bruised and placed over painful spots for up to an hour as a counter irritant to any kind of fixed internal pain. Pulverised roasted rootbark is rubbed into incisions on fractures and sprains	Bryant (1966); Hutchings et al. (1996)	2
Dittrichia graveolens (L.) Greuter. Synonyms: *Conyza minor* Bubani, *Jacobaea graveolens* (L.) Merino	Stinkwort, stinking fleabane (E), kakiebos (A)	Asteraceae	Shrub	ns	Used as a wash for pain and inflammation	Hulley and Van Wyk (2019)	1

Botanical name	*Common name	Family	Life-form	#Part used	Application	Reference	Nm
Dodonaea viscosa Jacq var. *angustifolia* (L.f) Benth. Synonyms: *Dodonaea arabica* Hochst. & Steud., *Dodonaea angustifolia* L.f.	Sand olive (E), makkaree, Sandolien, ysterhouttoppe (A), mutata-vhana (V)	Sapindaceae	Shrub	Leaves	Leaf decoctions are used against arthritis. Infusion of leafy tips are used for back pain. Powdered leaves used as snuff to help with headache. Leaf infusions used for pain and inflammation, backache and arthritis. The leaf tops (±3 teaspoons in 1 L boiling water) are made into an infusion and small amount taken 3 times daily to treat arthritis, inflammation and rheumatism	Van Wyk et al. (1997); Watt and Breyer-Brandwijk (1962); Van Wyk et al. (2008); De Beer and Van Wyk (2011); Hulley and Van Wyk (2019); Philander (2011); Thring and Weitz (2006); Mintsa Mi Nzue (2009)	8
Dolichothrix ericoides (Lam.) Hilliard & B.L.Burtt. Synonyms: *Aphelexis ericoides* Sweet, *Xeranthemum ericoides* Lam.	klipanoster, berganoster, langbeenanosterbos (A)	Asteraceae	Herb	ns	Infusions used for treating backache and pain	Hulley and Van Wyk (2019)	1
*Dovyalis caffra (*Hook.f. & Harv.) Sim. Synonyms: *Aberia caffra*, *Dovyalis caffra* (Hook. f. & Harv.) Warb.	Kei-apple, Dingaan's apricot, wild apricot (E), Kei-appel, appelkoosdoring (A), mutunu (V), Umqokolo (Z)	Salicaceae	Shrub	Root, bark, thorn	Roots and thorns are used for treating chest pain. Decoction of the bark and root is a remedy for rheumatism. Analgesic (sharp internal body pains). Thorn decoction is drunk for pain in chest (heart side)	Bryant (1966); Cumes et al. (2009); Watt and Breyer-Brandwijk (1962); Mhlongo and Van Wyk (2019); Arnold and Gulumian (1984)	5
Dovyalis rhamnoides (Burch. ex DC.) Burch. ex Harv. & Sond. Synonyms: *Flacourtia rhamnoides* Burch. ex DC.,	Cape cranberry, crownberry, sourberry, wineberry (E), suurbessie, wynbessie (A), isihlamane,	Salicaceae	Shrub	Roots, bark	Root and bark decoctions are taken for the pain in rheumatic fever and rheumatism	Bryant (1966)	1

Botanical name	*Common name	Family	Life-form	#Part used	Application	Reference	Nm
Dovyalis zizyphoides E.Mey. ex Arn.	ukhanginqi, umkokolo, umnyezane (Z)						
Dracaena aletriformis (Haw.) Bos. Synonyms: *Dracaena hookeriana, Dracaena latifolia* Regel, *Pleomele hookeriana* (Kuntze) N.E.Br.	Large-leaved dragon tree (E), Isikhothakhotha, Iskhothakhotha esikhulu, isikhothakhotha esincane, Ufayibe (Z)	Asparagaceae	Tree	ns	Analgesic (toothache), musculo-skeletal (inflammation)	Mhlongo and Van Wyk (2019)	1
Drimia altissima (L.f.) Ker Gawl. Synonyms: *Urginea altissima, Drimia paolii* Chiov.	Tall white squill (E), Jeukbol (A), maerman, isiklenama, umahlogolosi, umgulube (Z)	Asparagaceae	Herb	Bulbs	Bulbs are used as poultices for rheumatic swellings and gouty limbs	Watt and Breyer-Brandwijk (1962)	1
Drimia elata Jacq. Synonyms: *Drimia alta* R.A.Dyer, *Drimia robusta* Baker	Satin Squill (E), brandui, maerman (A), indongana-zibomvana, isiklenama (Z)	Asparagaceae	Herb	Bulbs	Bulb scales are rubbed on the chest for stabbing pains. Poultice of the bulb is used against pain and inflammation. Topical arthritis remedy	Hutchings et al. (1996); Hulley and Van Wyk (2019); Philander (2011)	3
Drimiopsis maculata Lindl. & Paxton. Synonyms: *Ledebouria petiolata* J.C.Manning & Goldblatt, *Drimiopsis minor* Baker	Leopard lily, little white soldiers, small snake lily (E), Ikhambi lezingane, Imbiza yezingane, Umayihlandlana, Umbola, U-anyanisi (Z)	Asparagaceae	Herb	ns	Musculo-skeletal (inflammation)	Mhlongo and Van Wyk (2019)	1
Dysphania ambrosioides (L.) Mosyakin & Clemants. Synonyms: *Ambrina ambrosioides* (L.) Spach, *Atriplex ambrosioides* f. *minus* Aellen	Mexican tea (E), Ikhambi leslumo (Z)	Amaranthaceae	Herb	Leaves, ns	Analgesic (fever). Leaves used as bud for earache and toothache. Leaves used as a compress for pain and inflammation	Mhlongo and Van Wyk (2019); Hulley and Van Wyk (2019)	2
Ekebergia capensis Sparrm. Synonyms: *Ekebergia buchananii* Harms, *Trichilia ekebergia* E. Mey. ex Sond.	Cape ash, dog plum (E), essenhout, rooiess(en)hout (A), nyamaru (TW), mmidibidi (NS), Mutovuma (V), umnyamathi,	Meliaceae	Tree	Roots, bark, leaves	Roots are used for headaches. Leaves and bark are used for headache. Bark is macerated and used as	Watt and Breyer-Brandwijk (1962); Mabogo (1990); Arnold	3

Botanical name	*Common name	Family	Life-form	#Part used	Application	Reference	Nm
	umthoma, usimanaye, uvungu (Z)				enema to relieve backache for 2 days	and Gulumian (1984)	
Elephantorrhiza burkei Benth.	Elephant-root, sumach bean (E), basboontjie, basbos (A), lesitsane (NS), mositsane, mosidi, mosidi-godimo, mosidi-mogolo (TW), tshisesevhafa, gumulolo, musesevhufa (V)	Fabaceae	Tree	Roots	Root decoction is ised for enema on swollen legs due to stomach troubles	Arnold and Gulumian (1984)	1
Elephantorrhiza praetermissa J.H.Ross	Sekhukhune Elephant-root (E)	Fabaceae	Shrub	ns	Decoction is used to treat stomach pains	Mogale et al. (2019)	1
Eleutherine bulbosa (Mill.) O.Urb. Synonyms: *Bermudiana bulbosa* (Mill.) Molina, *Eleutherine plicata* Herb. ex Klatt	Ingqunda, Uhloyile (Z)	Iridaceae	Herb	ns	Analgesic (body pains), musculo-skeletal (inflammation)	Mhlongo and Van Wyk (2019)	1
Emex australis Steinh. Synonyms: *Emex centropodium* Meisn, *Emex podocentrum* Meisn. ex Drège	Cape Spinach, Cat's Head, Devil's Thorn (E), Dubbeltjie, Dubbeltjiedoring (A), Umanyiwa yinkomo, Unginyathele, Unkunzana (Z)	Polygonaceae	Herb	ns	Analgesic (toothache), musculo-skeletal (inflammation)	Mhlongo and Van Wyk (2019)	1
Encephalartos natalensis R.A.Dyer & Verdoorn	Natal cycad (E), natalbroodboom (A), Isigqiki semfene, Isgqiki somkhovu (Z)	Zamiaceae	Tree	ns	Analgesic (sharp internal body pains)	Mhlongo and Van Wyk (2019)	1
Encephalartos villosus Lem.	Poor man's cycad (E), Isgqiki somkhovu, Isgqiki semfene, Umasundwana (Z)	Zamiaceae	Tree	ns	Analgesic (sharp internal body pains)	Mhlongo and Van Wyk (2019)	1
Englerophytum natalense (Sond.) T.D.Penn. Synonyms: *Amorphospermum natalense*	Natal. silverleaf milkplum (E), Natalmelkpruim (A),	Sapotaceae	Tree		Used for treating toothache	Mbanjwa (2020)	1

Botanical name	*Common name	Family	Life-form	#Part used	Application	Reference	Nm
(Sond.) Baehni, *Neoboivinella natalensis* (Sond.) Aubrév. & Pellegr.	ithunga, umThungwane (X), umThongwane (Z)						
Eriocephalus africanus L. Synonyms: *Eriocephalus corymbosus* Moench, *Eriocephalus umbellulatus* DC.	Wild rosemary (E), wilderoosmaryn, kapokbos (A.)	Asteraceae	Shrub	Leaves, stem	Treatment for migraine and headache	Philander (2011); Mintsa Mi Nzue (2009)	2
Eriocephalus ericoides (L.f.) Druce. Synonyms: *Eriocephalus glaber* var. *glaber, Tarchonanthus ericoides* L.f.	Rosemary, Kapok Bush (E), Kapokbos, Regtekapok, Renosterveldkapok, wilderoosmaryn, Sandveldkapokbossie (A)	Asteraceae	Shrub	ns	Used for backache, pain and inflammation	Hulley and Van Wyk (2019)	1
Eriocephalus punctulatus DC. Synonyms: *Eriocephalus pteronoides* Sch.Bip., *Eriocephalus punctulatus* var. *punctulatus*	Wild Rosemary, Cape chamomile (E), Boegoekapok (A)	Asteraceae	Shrub	Leaves	Treatment for migraine	Philander (2011)	1
Eriospermum lanceifolium Jacq. Synonyms: *Eriospermum brevipedicellatum* (Kuntze) Poelln., *Eriospermum lanceifolium* Jacq. var. *brevipedicellatum* Kuntze	Baboon ear (E), babbejaanore (A)	Asparagaceae	Herb	Bulbs	Ingested to alleviate pain	Philander (2011)	1
Erythrina caffra Thunb.	Coast coral tree (E), kuskoraalboom (A), Umsinsi, Umnsinsi (Z)	Fabaceae	Tree	Bark, ns	Analgesic (toothache, earache). The bark is used for treating toothache	Mhlongo and Van Wyk (2019); Mbanjwa (2020)	2
Erythrina lysistemon Hutch. Synonym: *Erythrina caffra* var. *mossambicensis*	Common coral tree, lucky bean tree (E), gewone koraalboom, kanniedood (A), umsintsi (X), muvhale (V), mophete (TW), umsinsi (Z)	Fabaceae	Tree	Bark	Bark is used to treat arthritis and toothache. Bark is used as a poultice for swellings and abscesses. The bark s using toothache	Pujol (1990); Mabogo (1990); Van Wyk et al. (1997); Mbanjwa (2020)	4

Botanical name	*Common name	Family	Life-form	#Part used	Application	Reference	Nm
Erythrophleum lasianthum Corbishley. Synonyms: *Erythrophleum guineense* G. Don var. *swaziense* Burtt Davy; *Erythrophleum suaveolens sensu* Compton	Maputaland ordeal tree, Swazi ordeal tree, sasswood (E), Maputaland-oordeelboom, Swazi-oordeel boom (A), Umbhemise, Umhlakazane, Umkhwangu (Z)	Fabaceae	Tree	Bark	Powdered bark is widely taken as snuff for headaches and persistent body pain. Decoctions are taken for intestinal spasms in doses limited to one tablespoon	Gerstner (1941); Pujol (1990)	2
Eucalyptus camaldulensis Dehnh. Synonyms: *Eucalyptus acuminata* Hook, *Eucalyptus mcintyrensis* Maiden	River red gum (E), Ugamthrini, Ugamthrini omhlophe (Z)	Myrtaceae	Tree	ns	Analgesic (headache, toothache). Used for relieving menstrual pain	Mhlongo and Van Wyk (2019); Mbanjwa (2020)	2
Eucalyptus globulus Labill. Synonyms: *Eucalyptus gigantea* Dehnh., *Eucalyptus perfoliata* Desf.	Eucalyptus	Myrtaceae	Tree	Leaves	For treating muscle aches and pain	Philander (2011)	1
Eucalyptus grandis W.Hill	looded gum, rose gum (E), Ugamthrini obomvu, Ugamthrini (Z)	Myrtaceae	Tree	ns	For relieving menstrual pain	Mbanjwa (2020)	1
Eucalyptus sideroxylon A.Cunn. Synonyms: *Eucalyptus leucoxylon* var. *minor* Benth, *Eucalyptus sideroxylon* var. *rosea* Rehder	Mugga ironbark, red ironbark (E), Bloekom (KS)	Myrtaceae	Tree	Leaves	leaf infusion used for backache	Nortje and van Wyk (2015)	1
Euclea natalensis A.DC. Synonyms: *Euclea natalensis* subsp. *natalensis*, *Euclea natalensis* subsp. *obovata* F.White	Natal guarri, Natal ebony, large-leaved guarri (E), berggwarrie, swartbasboom (A), umTshekisani, umKhasa (X), Mutangulethavha (V), umZimane, umTshikisane, umHlalanyamazane, umAnyathi (Z)	Ebenaceae	Tree	Roots, bark	Roots are burned and teh smoke inhaled to relieve headache. Roots are powdered and placed on incisions made on forehead to relieve headache. Infusion made from the roots and bark is used to wash the oral cavity for toothache	Arnold and Gulumian (1984)	1
Euclea undulata Thunb. Synonyms: *Euclea humilis* Eckl.	small-leaved guarri, common guarri (E),	Ebenaceae	Tree	Roots, leaves	Infusions used as treatment for stomachache and	Hulley and Van Wyk (2019)	1

Botanical name	*Common name	Family	Life-form	#Part used	Application	Reference	Nm
& Zeyh.. *Euclea undulata* var. *myrtina* (Burch.) Hiern	kleinblaarghwarrie, gewone ghwarrrie (A), gwanxe, inkunzane, umshekizane, umbophanyamazane (Z)				rheumatism. Leaves used as treatment for stomach ailments, chest ailments, infusion		
Eucomis autumnalis (Mill.) Chitt. Synonyms: *Basilaea undulata (Aiton)* Mirb, *Eucomis undulata* Aiton	Pineapple flower, pineapple lily (E), wildepynappel, krulkoppie (A), Ukhwali, Umathunga,Umathunga obovu (Z)	Asparagaceae	Herb	ns	Analgesic (sharp internal body pains, back pains, general body pains)	Mhlongo and Van Wyk (2019)	1
Eucomis autumnalis (Mill.) Chitt. subsp. *autumnalis*. Synonym: *Eucomis undulata* Ait.	Pineapple flowers, pineapple lily (E), Wildepynappel, krulkoppie (A), Mothusi, Phato (SS), ubuhlungu becanti (X), umakhondle (Z)	Asparagaceae	Herb	Bulbs, roots	Milk or water decoctions of bulb shavings and roots are taken for colic and abdominal distension. Bulbs are used for treating abdominal pains	Hutchings et al. (1996); Watt and Breyer-Brandwijk (1962)	2
Eucomis comosa (Houtt.) Wehrh. var. *comosa*. Synonyms: *Eucomis pallidiflora* Bak, *Eucomis punctata* L'Herit.	Pineapple flower (E), krulkoppie, Pynappellelie (A), Ubuhlungu-becanti (X), Ubuhlungu-becanti (Z)	Asparagaceae	Herb	Roots, bulbs	Medicine made from the root is administered as an anti-rheumatic in doses of one spoonful. Bulb decoctions are used for rheumatism	Gerstner (1941); Hutchings et al. (1996); Watt and Breyer-Brandwijk (1962)	3
Eulophia ovalis Lindl. Synonyms: *Eulophia bakeri* Rolfe; *Eulophia dregeana* Lindl.; *E. haygarthii* Rolfe	Lekholela, Lekholela-la-Matebele, Lekoesha (SS), Ihamba Lesigodi, Iphamba (Z)	Orchidaceae	Herb	Tuber	Powder from burnt tubers is rubbed into incisions on sore limbs	Hutchings et al. (1996)	1
Euphorbia bupleurifolia Jacq. Synonyms: *Tithymalus bupleurifolius* (Jacq.) Haw, *Euphorbia proteifolia* Boiss.	Cycad spurge (E), melkbol (A), insema, intsele (X), inkamamasane, insema (Z)	Euphorbiaceae	Herb	Roots	Ashes from dried burnt roots are rubbed into incisions made around swellings of the lower limbs	Bryant (1966)	1
Euphorbia ingens E.Mey. ex Boiss. Synonym: *Euphorbia similis* A.Berger	Common tree Euphorbia (E), naboom, gewone naboom (A), Umnhlonhlo, Umphapha (Z)	Euphorbiaceae	Tree	Roots, ns	Roots are burned and the ash rubbed on swollen ankles. Musculo-skeletal (inflammation)	Mhlongo and Van Wyk (2019); Arnold and Gulumian (1984)	2
Euphorbia mauritanica L. Synonyms: *Euphorbia*	Yellow milk bush, golden spurge (E), geelmelkbos,	Euphorbiaceae	Shrub	Roots	Infuse the root in boiling water and rinse mouth with	Van Wyk et al. (2008)	1

Botanical name	*Common name	Family	Life-form	#Part used	Application	Reference	Nm
corallothamnus Dinter, *Tirucalia mauritanica* (L.) P.V.Heath	beesmelkbos, gifmelkbos, kaalmelkbos, kleinmelkbos, melkbos (A)				the warm extract to mitigate toothache		
Euphorbia natalensis Bernh. ex Krauss	Inkalamasane, Inkamamasane, Umnhlonhlo (Z)	Euphorbiaceae	Shrub	Root	For treating toothache	Mintsa Mi Nzue (2009)	1
Euryops lateriflorus (L.f.) DC. Synonyms: *Jacobaeastrum lateriflorum* (L.f.) Kuntze, *Othonna lateriflora* L.f.	Soetharpuisbos, Vetrepuis, Vetbos, Vetharpuis (A), Blourapuis (KS)	Asteraceae	Shrub	Bark (resin)	The resin is used for toothache	Nortje and van Wyk (2015)	1
Exomis microphylla (Thunb.) Aellen. Synonyms: *Atriplex microphylla* Willd., *Exomis microphylla* var. *microphylla*	Basterhondebossie, Brakbos, Brakbossie, Hondebos, Hondebossie, Hondepisbossie, Vaalbrakbossie (A)	Amaranthaceae	Shrub	Leaves	Used to treat stomach ailments, ear-ache, rheumatism and arthritis. Infusion or compress of leaves used to treat fever	Hulley and Van Wyk (2019)	1
Ficus craterostoma Warb. ex Mildbr. & Burret. Synonyms: *Ficus anomani* Hutch., *Ficus ruwenzoriensis* De Wild.	Forest fig, forest strangler-fig, blunt-leaved forest fig, bastard Natal fig (E), bosvy, wurgvy, stomblaarbosvy, basternatalvy (A), intozane, uluzi, inTendekwane, umthombe (X), umthombe, umbombe, isihlamfane (Z), moumo (NS), muvumo, tshikululu (V)	Moraceae	Tree	Leaves	Fresh leaf decoction is taken orally in small quantities once or twice daily for stomaachache	Bhat and Jacobs (1995)	1
Ficus ingens (Miq.) Miq. Synonyms: *Ficus caffra* (Miq.) Miq, *Ficus ovatocordata* De Wild.	Red-leaved rock fig (E), rooiblaarvy (A), mohlatsa (NS); Umanyala, Umkhiwane (Z)	Moraceae	Tree	ns	Analgesic (sharp internal body pains)	Mhlongo and Van Wyk (2019)	1
Fockea sp	kambro; camarebi; camao (A)	Apocynaceae	Herb	Tuber	Fleshy tuber is use to treat headache	De Beer and Van Wyk (2011)	1

Botanical name	*Common name	Family	Life-form	#Part used	Application	Reference	Nm
Foeniculum vulgare Mill. Synonyms: *Anethum foeniculum* L, *Foeniculum officinale* ALL.	Fennel, vinkel (E), i(li)beka, imbozisa, imboziso(-eluhlaza) (Z)	Apiaceae	Herb	Leaves	Leaf decoctions are taken three times a day or taken as enemas for pain in the side. Used for cramp and stomach ache. Analgesic (toothache), Musculo-skeletal inflammation. Infusion of the leaves is used for stomachache and arthritis	Hutchings et al. (1996); Watt and Breyer-Brandwijk (1962); Mhlongo and Van Wyk (2019); Hulley and Van Wyk (2019); Thring and Weitz (2006)	5
Furcraea foetida (L.) Haw. Synonyms: *Agave bulbosa* K.Koch, *Furcraea gigantea* Vent.	Giant Cabuya, Green-aloe, Mauritius-hemp (E), Isitaluka, Ubhumlane, Ufayibe (Z)	Asparagaceae	Shrub	ns	Musculo-skeletal inflammation	Mhlongo and Van Wyk (2019)	1
Galenia africana L. Synonyms: *Galenia linearis* Thunb., *Galenia tenuifolia* Salisb.	Geelbos, perdebos, kraalbos (A), Kraalbos (KS)	Aizoaceae	Shrub	Leaves, twigs	Twig/leaf placed in tooth for toothache. Bathe in a weak infusion to relieve rheumatism. Leaf infusion used to treat leg pain. Used as wash or ointment as a rinse for toothache, rheumatism, pain and inflammation	Nortje and van Wyk (2015); Van Wyk et al. (2008); De Beer and Van Wyk (2011); Hulley and Van Wyk (2019); Philander (2011)	5
Galium capense Thunb. subsp. *namaquense* (Eckl. & Zeyh.) Puff. Synonyms: *Galium capense* var. *scabrum* Sond., *Galium namaquense* Eckl. & Zeyh.	Tiny-tots (E), Kleefgras (A), Rooistorm (KS)	Rubiaceae	Herb	Roots	Used as snuff for headache	Nortje and van Wyk (2015)	1
Galium tomentosum Thunb. Synonyms: *Galium asperum var. villosum* Eckl. & Zeyh., *Galium glabrum* Thunb.	Old Man's Beard (E), Rooivergeet, Kleefgras (A), Jantjiegoub (KS)	Rubiaceae	Herb	Roots	Roots are powdered and use as a snuff for headache. As a remedy for inflammation	Nortje and van Wyk (2015); Hulley and Van Wyk (2019); Philander (2011)	3

Botanical name	*Common name	Family	Life-form	#Part used	Application	Reference	Nm
Garcinia livingstonei T.Anderson. Synonyms: *Garcinia angolensis* Vesque, *Garcinia livingstonei* var. *pallidinervia* Engl.	African mangosteen (E), Afrika-geelmelkhout (A), Muphiphi, mupimbi (V), umPhimbi, uGobandlovu (Z)	Clusiaceae	Tree	Roots	Root decoction is used for toothache	Arnold and Gulumian (1984)	1
Gasteria brachyphylla (Salm-Dyck) van Jaarsv. Synonyms: *Aloe pseudonigricans* Salm-Dyck, *Gasteria triebneriana* Poelln.	Klein Karoo ox-tongue (E), Klein Karoo-beestong, boesmanrys (A)	Xanthorrhoeaceae	Herb	Leaves	Compress on sores and sore foot	Hulley and Van Wyk (2019)	1
Gazania krebsiana Less. Synonyms: *Gazania mucronata* DC., *Meridiana oxyloba* (DC.) Kuntze	terracotta gazania (E), gousblom, botterblom, rooi gazania (A), Ubendle, Umasonga (Z)	Asteraceae	Herb	ns	Internal side pain in children	Mhlongo and Van Wyk (2019)	1
Gerbera piloselloides (L.) Cass. Synonyms: *Arnica hirsuta* Forssk., *Gerbera peregrina* Steenis	Pink and white gerbera (E), botterblom, Griekwateebossie (A), moarubetso (SS), Imbune yentaba, Uhlunguhlungu, Umoyawezwe (Z)	Asteraceae	Herb	ns	Musculo-skeletal (inflammation). Used for treating headache and ear-ache	Mhlongo and Van Wyk (2019); Mbanjwa (2020)	2
Gladiolus dalenii Van Geel. Synonyms: *Gladiolus adlami* Baker, *Gladiolus hockii* De Wild.	African gladiolus (E), papegaai-gladiolus (A), khahla-e-kholo (SS), Isidwa, Isiqunga sikatikoloshe, Umabelejongosi, Umasendenja, Umlunge, Undwendweni (Z)	Iridaceae	Herb	Bulbs, ns	Musculo-skeletal (back problem). Bulb decoction is used as ear drops to relieve ear-ache	Mhlongo and Van Wyk (2019); Arnold and Gulumian (1984)	2
Gladiolus ecklonii Lehm. Synonyms: *Gladiolus ecklonii* subsp. *ecklonii, Gladiolus inclusus* F.Bolus	sheathed gladiolus (E), kxahla, litsoantsoang, makhabebe (SS), Ibuthe, Isidwa, Incwincwi, Umabelejongosi, Umlunge (Z)	Iridaceae	Herb	ns	Musculo-skeletal (back problem)	Mhlongo and Van Wyk (2019)	1

Botanical name	*Common name	Family	Life-form	#Part used	Application	Reference	Nm
Glycyrrhiza glabra L. Synonyms: *Glycyrrhiza brachycarpa* Boiss., *Glycyrrhiza violacea* Boiss. & Noe	Licorice (E), soethoutwortel (A)	Fabaceae	Herb	ns	Used to treat inflammation and stomach ailments	Hulley and Van Wyk (2019)	1
Gnidia capitata L.f. Synonyms: *Lasiosiphon capitatus* (L. f.) Burtt Davy, *Lasiosiphon similis* C.H. Wright	Gifbos (A)	Thymelaeaceae	Shrub	Bark	For treating menstrual pains	Philander (2011)	1
Gnidia deserticola Gilg. Synonyms: *Arthrosolen deserticola* (Gilg) Compton, *Lasiosiphon deserticola* (Gilg) C.H. Wright	Hotnotsverfbossie, Saffraan, Saffraanbos, Verfbossie (A)	Thymelaeaceae	Shrub	ns	Used for the treatment of pain and inflammation	Hulley and Van Wyk (2019)	1
Gnidia kraussiana Meisn. Synonyms: *Gnidia hoepjiwriana*, *Lasiosiphon hoepfnerianus*, *Lasiosiphon kraussianus* (Meisn.) Burtt Davy	Yellow heads (E), gifbossie (A), umarhedeni (X), isidikili, imfuzane, umsilawengwe (Z)	Thymelaeaceae	Herb	Roots	Strong enemas made from root extracts are taken for stomach complaints. Roots in milk decoctions for backache and stomach sores. Root decoctions or infusions are taken for chest complaints. For lower back pain	Hulme (1954); Watt and Breyer-Brandwijk (1962); Hutchings et al. (1996); Mbanjwa (2020)	4
Gomphocarpus cancellatus (Burm.f.) Bruyns. Synonyms: *Asclepias arborescens* L., *Asclepias rotundifolia* Mill.	Wild cotton (E), bergmelkbos, bergtontelbos, dermhout, gansiesbos, katoenbos, melkbos, regopmelkbos (A), Bergtontelbos (KS)	Apocynaceae	Herb	Leaves	Applied as ointment on pains	Nortje and van Wyk (2015)	1
Gomphocarpus fruticosus (L.) W.T.Aiton. Synonyms: *Asclepias fruticosa*, *Gomphocarpus crinitus* G.Bertol.	Milkweed, narrow-leaved cotton bush, wild cotton (E), blaasoppies (A), Gewone (KS), ulusinga Iwesalukazi, umsinga-lwesalukazi (Z)	Apocynaceae	Shrub	Leaves, roots	Leaf infusions are administered for stomach pain in children. Roots are used for general body pain and stomach ache. Root used as snuff for headache, dry leaves used as snuff for	Gerstner (1941); Hulme (1954); Pujol (1990); Watt and Breyer-Brandwijk (1962); Nortje and van Wyk	7

Botanical name	*Common name	Family	Life-form	#Part used	Application	Reference	Nm
					headache. Leaf decoction is taken orally as headache treatment	(2015); De Beer and Van Wyk (2011); Mogale et al. (2019)	
Gomphocarpus physocarpus E.Mey. Synonyms: *Asclepias brasiliensis* (E.Fourn.) Schltr., *Gomphocarpus brasiliensis* E.Fourn.	Balloon milkweed, balloon wild cotton, hairy balls, bindweed (E), balmelkbossie, balbossie, wilde kapok (A), Usinga, Uqhumane, Usingalwesalukazi, Usipha lwesalukazi (Z)	Apocynaceae	Shrub	Leaves, ns	Analgesic (headache, internal side pains). Smoked leaves is used for headache	Mhlongo and Van Wyk (2019); Mbanjwa (2020)	2
Gunnera perpensa L. Synonyms: *Gunnera calthifolia*, *Perpensum blitispermum*	River pumpkin, wild Rhubarb (E), Iphuzi, Uxobo (X), Rivierpampoen (A), qobo (SS), Izibu, Ugobho, Uklenya, Uxobo (Z)	Gunneraceae	Herb	Roots, rhizomes	Together with other plants such as *Alepidea amatymbica* and *Crinum* sp., the root decoctions are taken for pain in rheumatic fever and stomachache. Musculo-skeletal (inflammation). Inflammtion and menstrual pain. Root decoction is used to treat menstrual pain. Leaves used as compress on pain and inflammation especially rheumatism and backache. Used as compress for headache. Used for relieving menstrual pains and afterbirth pain	Bryant (1966); Mhlongo and Van Wyk (2019); Maroyi (2017); Bhat (2014); Hulley and Van Wyk (2019); Mbanjwa (2020)	6
Gymnosporia rubra (Harv.) Loes. Synonyms: *Maytenus mossambicensis* var. *rubra* (Harv.) Blakelock, *Celastrus ruber* Harv.	Red-flower Spikethorn, Red-flowered Spike-thorn (E), Rooiblom-pendoring (A), Ihlinzanyoka, Ithethe (Z)	Celastraceae	Tree	ns	Analgesic (internal sharp body pains), musculo-skeletal (inflammation)	Mhlongo and Van Wyk (2019)	1

Botanical name	*Common name	Family	Life-form	#Part used	Application	Reference	Nm
Gymnosporia senegalensis (Lam.) Loes. Synonyms: *Catha europaea* Boiss., *Celastrus senegalensis* Lam.	Confetti Spike-thorn, Confetti Tree, Red Spike-thorn (E), Rooidoring, Rooi-pendoring (A), Motlhono, Motlhôno, Motlhonu (TW), Sephatwa (NS), Tshibavhe, Tshiphandwa (V), Isihlangu, Isihlangwane, Ubuhlangwe (Z)	Celastraceae	Shrub	Leaves	Leaves are used to treat stomachache	Mongalo and Makhafola (2018)	1
Haemanthus albiflos Jacq. Synonyms: *Haemanthus albiflos* var. *brachyphyllus* Baker, *Haemanthus virescens* var. *pubescens* Herb.	white paint brush (E), witpoeierkwas (A), umathunga (X), uzaneke (Z)	Amaryllidaceae	Herb	Whole plant	Use for treating pains and arthritis	Mintsa Mi Nzue (2009)	1
Harpagophytum procumbens (Burch.) DC. ex Meisn	Devil's claw, harpago, grapple plant (E), bobbejaandubbeltjie, kloudoring (A), lekgagamare, ghamaghoe (TW)	Pedaliaceae	Creeper	Whole plant	Indicated as a treatment for rheumatism	Van Wyk and Gericke (2000)	1
Harpephyllum caffrum Bernh.	Wild plum (E), wildepruim, kafferpruim (A), umgwenya (X), mothekele (NS), umgwcnya (Z)	Anacardiaceae	Tree	Bark	Powdered burnt bark is use to treat sprains and fractures. Analgesic (lower back aches)	Hutchings et al. (1996); Mhlongo and Van Wyk (2019)	2
Helichrysum aureonitens Sch.Bip.	golden everlasting (E), goue sewejaartjie (A), toane-ntja, toane-poli (SS), Impepho, Intungwa (Z)	Asteraceae	Herb	ns	Analgesic (toothache), musculo-skeletal (inflammation) and headache	Mhlongo and Van Wyk (2019); Mbanjwa (2020)	2
Helichrysum cymosum (L.) D.Don. Synonyms: *Gnaphalium cernuum* Thunb., *Gnaphalium tricostatum* Sieber ex DC.	Gold carpet (E), goue tapyt (A), imPepho, imPepha (X)	Asteraceae	Herb	Leaves	Fresh leaves are boiled in water and the vapour used as vapour bath for treating headache. For treating toothache	Bhat and Jacobs (1995); Bhat (2013); Mbanjwa (2020)	3

Botanical name	*Common name	Family	Life-form	#Part used	Application	Reference	Nm
Helichrysum luteoalbum (L.) Rchb. Synonyms: *Gnaphalium luteoalbum* L., *Pseudognaphalium luteoalbum* (L.) Hilliard & B.L.Burtt	Jersey cudweed (E), Impepho, Impepho emhlophe/ enkulu (Z)	Asteraceae	Herb	ns	Musculo-skeletal (inflammation)	Mhlongo and Van Wyk (2019)	1
Helichrysum nudifolium (L.) Less. Synonyms: *Anaxeton nudifolium* (L.) Gaertn., *Helichrysum asperifolium* Moeser, *Helichrysum leiopodium* DC.	Hottentot's tea, kaffir tea (E), Hottentotstee (A), letapiso, mohlomela-tsie-oa-thaba (SS), icholocholo, icolocolo (X); isidwaba-somkhovu, umagada-emthini (Z)	Asteraceae	Herb	Leaves	Menstrual pain. Fresh leaves are boiled and the hot lotion is used to foment swollen feet for rapid recovery	Maroyi (2017); Bhat (2013)	2
Helichrysum odoratissimum (L.) Sweet. Synonyms: *Gnaphalium strigosum* Thunb., *Helichrysum rosmarinum* Mattf.	Most fragrant helichrysum (E), kooigoed, kruie (A), iphepho (X), Hotnotskooigoed (KS)	Asteraceae	Herb	Whole plant, ns	Infusion used for backache. Whole plant is used for headache. Used for treating pain and inflammation, backache, toothache, menstrual pains and cramp	Nortje and van Wyk (2015); Maroyi (2017); Hulley and Van Wyk (2019); Mbanjwa (2020)	4
Helichrysum patulum (L.) D.Don. Synonyms: *Gnaphalium divaricatum* P.J.Bergius, *Helichrysum crispum* (L.) D.Don	honey everlasting (E), kooigoed (A), impepho (X)	Asteraceae	Herb	Leaves	Dried leaf infusions drunk as infusion, approx 25 ml taken 2–3 times per day until symptoms of arthritis, headache and rheumatism to disappear	Thring and Weitz (2006)	1
Helichrysum pedunculatum Hilliard & B.L.Burtt. Synonyms: *Helichrysum pedunculare* (L.) DC., *Helichrysum pedunculare* var. *pedunculare*	Indlebe zebokwe, uNdleni, isicwe, siggutsi (X)	Asteraceae	Herb	Leaves	Leaves are used after circumcision to prevent inflammation externally	Bhat and Jacobs (1995); Bhat (2014)	2
Helichrysum pumilio (O.Hoffm.) Hilliard & B.L.Burtt. Synonyms: *Helichrysum bolusianum*	Sewejaartjie, wolbossie (A)	Asteraceae	Herb	Whole plant	Prepare as infusion and drank to treat rheumatism	Van Wyk et al. (2008)	1

Botanical name	*Common name	Family	Life-form	#Part used	Application	Reference	Nm
Moeser, *Helichrysum laneum* S.Moore							
Helichrysum rutilans (L.) D.Don. Synonyms: *Gnaphalium rutilans* L., *Helichrysum manopappum* O.Hoffm.,	Kooigoedbos (KS)	Asteraceae	Herb	ns	infusion used for backache	Nortje and van Wyk (2015)	1
Helinus integrifolius (Lam.) Kuntze. Synonyms: *Helinus ovatus, Helinus scandens*	Soap bush, soap creeper (E), seepbos, seepbossie (A), tyolo, Ukumbuqwekwe (X), ubhubhubhu, ubububu (Z)	Rhamnaceae	Shrub	Roots	Root decoctions are taken for backache	Hutchings et al. (1996)	1
Hermbstaedtia glauca (J.C.Wendl.) Rchb. ex Steud.	Bokhout (A), Bokhoutjie (KS)	Amaranthaceae	Shrub	ns	Used with *grashoutjie* for backache	Nortje and van Wyk (2015)	1
Heteromorpha arborescens (Spreng.) Cham. & Schltdl. Synonyms: *Heteromorpha arborescens* var. *collina* (Eckl. & Zeyh.) Sond., *Tenoria arborescens* (Spreng.) Spreng.	Parsley tree (E), wildepietersielie, pietersieliebos (A), umbangadlala, umbangandlatho, umbangeza, iyeza-lempambano (X), Muthatha-vhanna (V), umbangadlala (Z)	Apiaceae	Tree	Roots	Root decoction is used to prepare soft porridge to relieve general body pain and headache	Mabogo (1990)	1
Heteromorpha trifoliata (Wendl.) Eckl. & Zeyh. Synonyms: *Heteromorpha arborescens* var. *abyssinica, Bupleurum arborescens, Bupleurum trifoliatum*	Parsley tree (E), kraaibos, stinkbos, wildepieterselie (A), Mokadala (NS), Mula-notshi, Muthatha-vhanna (V), umbangandlala (X), umbangandlala, Umbhangandlahla (Z)	Apiaceae	Tree	Bark, roots	Bark is used for colic. Root infusions are used for headaches and pain in the chest and back. Smoke from burning unspecified part of the plant is inhaled for headaches	Gerstner (1941); Watt and Breyer-Brandwijk (1962)	2
Heteropyxis natalensis Harv.	Lavender Tree (E), Laventelboom (A), Umkhuze, Umkluza (Z)	Myrtaceae	Tree	ns	Analgesic (lower back pains)	Mhlongo and Van Wyk (2019)	1

Botanical name	*Common name	Family	Life-form	#Part used	Application	Reference	Nm
Hibiscus aethiopicus var. *ovatus* Harv. Synonyms: *Hibiscus leiospermus* Harv., *Hibiscus aethiopicus* var. *asperifolius* Hochr.	Dwarf yellow hibiscus (E), umhlophe, uvemvane (Z)	Malvaceae	Herb	Roots	Poultices from the pounded roots are applied to swollen joints and sprains	Hulme (1954); Watt and Breyer-Brandwijk (1962)	2
Hippobromus pauciflorus Radlk. Synonyms: *Hippobromus alata* (Thunb.) Eckl. & Zeyh., *Rhus alatum* Thunb.	Bastard Horsewood, False Horsewood, Horsewood (E), Basterperdepis, Basterperdepisboom (A), Isiqhume, Uqhume (Z)	Sapindaceae	Tree	Leaves, roots, stem, ns	Analgesic (internal side pains). Used for headache and toothache	Mhlongo and Van Wyk (2019); Mintsa Mi Nzue (2009)	2
Holarrhena pubescens Wall. ex G.Don. Synonyms: *Chonemorpha antidysenterica* (Roth) G.Don, *Holarrhena perrotii* Spire	Makhulu-wamuhatu, khatha-khathane (V)	Apocynaceae	Shrub	Roots	Root decoction is used to prepare porridge for 3 days to relieve stomachache in women	Arnold and Gulumian (1984)	1
Hoodia gordonii (Masson) Sweet ex Decne. Synonyms: *Scytanthus gordonii* (Masson) Hook., *Stapelia gordonii* Masson	bitter ghaap (E), bitterghaap, muishondghaap, wolweghaap, bobbejaanghaap, bergghaap, bokhorings (A)	Apocynaceae	Herb	Stem	To treat stomach pain. Used for the treatment of backache, headache and stomachache	De Beer and Van Wyk (2011); Hulley and Van Wyk (2019)	2
Hydnora africana Thunb. Synonyms: *Aphyteia africana* (Thunb.) Oken, *Hydnora longicollis* Welw.	Jackal food (E), jakkalskos, bobbejaankos (A), Jakkelskos (KS)	Hydnoraceae	Herb	Fruit	Dried fruit pericarp (*skil*) is ground and used as an infusion for body pains	Nortje and van Wyk (2015)	1
Hypoxis colchicifolia Baker. Synonyms: *Hypoxis distachya* Nel, *Hypoxis oligotricha* Baker	Broad-leaved hypoxis (E), iLabatheka (Z)	Hypoxidaceae	Herb	Bulbs, roots	For treating arthritis	Mintsa Mi Nzue (2009)	1
Hypoxis hemerocallidea Fisch., C.A.Mey. & Avé-Lall. Synonyms: *Hypoxis rooperi* S. Moore, *Hypoxis patula* Nel	African potato, Star flower, yellow star (E), sterblom, geelsterretjie, gifbol (A), moli kharatsa, lotsane (SS), tshuka (TW), inongwe, ixhalanxa, ikhubalo	Hypoxidaceae	Herb	Corms, rootstock	Infusions and decoctions of the plant are used for rheumatism. Juice from the rootstock is applied to burns. Corms are traditionally used for headaches. Analgesic	Hutchings et al. (1996); Van Wyk and Gericke (2000); Mhlongo and Van Wyk (2019); Philander	5

Botanical name	*Common name	Family	Life-form	#Part used	Application	Reference	Nm
	lezithunzela (X), inkomfe, inkomfe enkulu (Z)				(back pains, sharp internal body pains), musculo-skeletal (arthritis)	(2011); Mintsa Mi Nzue (2009)	
Hypoxis rigidula Baker. Synonyms: *Hypoxis cordata* Nel, *Hypoxis volkmanniae* Dinter	Yellow Star, Farmer's String, Silver-leaved Star-flower (E), Botterblom (A), Tieane (SS), Inkomfe, Umhungulo (Z)	Hypoxidaceae	Herb	ns	Analgesic (general body pains, sharp internal body pains)	Mhlongo and Van Wyk (2019)	1
Ilex mitis (L.) Radlk var. *mitis*. Synonyms: *Ilex capensis* Sond. & Harv., *Sideroxylon nigricans* Dum.Cours.	African/Cape holly, water tree (E), monamane (NS), umDuma (X), phukgu, phukgile (SS), mutanzwa-khamelo (V), iphuphuma, umdumowazo (Z)	Aquifoliaceae	Tree	ns	Used for rheumatism. Enemas made from unspecified parts are administered to children with colic	Hutchings et al. (1996); Watt and Breyer-Brandwijk (1962)	2
Ipomoea bolusiana Schinz. Synonyms: *Ipomoea angustisecta* Engl., I*pomoea simplex* auct. non Thunb.	Narrow-leaved pink ipomoea (E), Seakhoe (SS)	Convolvulaceae	Herb	Bulbs	Bulb is used to treat foot ache	Mongalo and Makhafola (2018)	1
Ipomoea cairica (L.) Sweet. Synonyms: *Convolvulus cairicus* L., *Ipomoea stipulacea* Jacq.	Coast morning glory, five-fingered morning glory, Cairo morning glory, Messina creeper, mile-a-minute, (E), Ijalamu, Ingcingolo (Z)	Convolvulaceae	Climber	ns	Analgesic (internal side pains)	Mhlongo and Van Wyk (2019)	1
Ipomoea crassipes Hook. Synonyms: *Ipomoea calystegioides* Hallier f., *Ipomoea sarmentacea* Rendle	Leafy-flowered Ipomea (E), Ijalamu, Uvimbukhalo (Z)	Convolvulaceae	Creeper	ns	Musculo-skeletal (inflammation)	Mhlongo and Van Wyk (2019)	1
Jamesbrittenia atropurpurea (Benth.) Hilliard. Synonyms: *Chaenostoma croceum* (Eckl. ex Benth.) Wettst. ex Diels,, *Sutera atropurpurea* (Benth.) Hiern	Bruinsafraanbos, Geelblommetjie (A)	Scrophulariaceae	Shrub	Leaves	Leaves used for toothache and general pains	De Beer and Van Wyk (2011)	1

Botanical name	*Common name	Family	Life-form	#Part used	Application	Reference	Nm
Jatropha curcas L. Synonyms: *Castiglionia lobata* Ruiz & Pav., *Ricinus americanus* Mill.	Physic nut, Barbados nut, poison nut, bubble bush or purging nut (E), mupfure-donga (V)	Euphorbiaceae	Shrub	Root	Root decoction is used to rinse the oral cavity to relieve toothache	Arnold and Gulumian (1984)	1
Juncus lomatophyllus Spreng. Synonyms: *Juncus capensis var. latifolius* E.Mey., *Juncus viridifolius* Adamson	Imfe-yesele (X)	Juncaceae	Herb	Leaves	Leaf decoction are applied externally to prevent inflammation of wound	Bhat and Jacobs (1995)	1
Kalanchoe pinnata (Lam.) Pers. Synonym: *Bryophyllum pinnatum* (Lam.) Oken	Air plant, cathedral bells, life plant, miracle leaf (E), Inyathelo, Umvuthuza (Z)	Crassulaceae	Herb	ns	Analgesic (tooth ache), musculo-skeletal (inflammation)	Mhlongo and Van Wyk (2019)	1
Kedrostis africana (L.) Cogn. Synonyms: *Kedrostis digitata* Cogn., *Kedrostis punctulata* Cogn.	Baboon's Cucumber (E), bojaankamoo, bitterpatat (A)	Cucurbitaceae	Climber	ns	Used to treat stomach ailment	Hulley and Van Wyk (2019)	1
Kedrostis capensis A. Meeuse. Synonym: *Pisosperma capense* Sond.	Bitterpatat (A)	Cucurbitaceae	Climber	ns	Used for the treatment of stomach ailments	Hulley and Van Wyk (2019)	1
Kedrostis foetidissima (Jacq.) Cogn. Synonyms: *Kedrostis foetidissima* subsp. *obtusiloba* Meeuse, *Rhynchocarpa foetida* C.B. Clarke	Ystervarkpatat (A)	Cucurbitaceae	Climber	ns	Used for the treatment of stomach ailments	Hulley and Van Wyk (2019)	1
Kedrostis leloja (Forssk. ex J.F Gmel.) C.Jeffrey	Makgonatšohle (SP)	Cucurbitaceae	Herb	Whole plant	Whole plant can be mixed with bathing water to treat aching body	Mogale et al. (2019)	1
Kedrostis nana var. *zeyheri* A. Meeuse. Synonym: *Kedrostis zeyheri* Cogn.	kalmoes, bitterhout (A)	Cucurbitaceae	Climber	Roots	Medicine for stomachache	Van Wyk et al. (2008); Hulley and Van Wyk (2019)	2
Kigelia africana (Lam.) Benth. Synonyms: *Bignonia africana*, *Kigelia pinnata*	Sausage-tree (E), kalabasboom (A), Mutshata, Muvevha (V),	Bignoniaceae	Tree	Bark, fruits, Stem, twigs	Bark is used for toothache. Powdered dried fruit is used for rheumatism. Stem and	Hutchings et al. (1996); Watt and Breyer-Brandwijk (1962)	2

Botanical name	*Common name	Family	Life-form	#Part used	Application	Reference	Nm
	umvungura, Umvongothi (Z)				twigs are rheumatism and other stomach ailment		
Lagenaria siceraria (Molina) Standl. Synonyms: *Cucumis bicirrha* J.R.Forst. ex Guill., *Lagenaria vulgaris* subsp. *asiatica* Kobjakova	bottle gourd, calabash (E), kalbas (A), moraka (NS), segwana (TW), Muphapha (V), iselwa (X, Z)	Cucurbitaceae	Creeper	Leaves	Cooked leaves are eaten by women as potherbs at the same time relieving period pains	Mokganya and Tshisikhawe (2019)	1
Landolphia kirkii Dyer. Synonyms: *Clitandra stapfiana* A.Chev., *Landolphia polyantha* K.Schum.	Rubber vine, Sand apricot-vine (E)	Apocynaceae	Shrub	Roots	The root is infused in water and the water is gargled to relieve toothache	Corrigan et al. (2011)	1
Lannea schweinfurthii Engl.	false-marula (E), valsmaroela (A), mmopu (NS), umganunkomo (Z)	Anacardiaceae	Tree	Roots	Root decoction is used for body aches	Tshikalange et al. (2016)	1
Lantana camara L. Synonyms: *Lantana undulata* Raf., *Lantana mexicana* Turner	tickberry (E), Tshidzimbampotolo (V)	Verbenaceae	Shrub	Roots	Dried roots are burned and smoked over private parts for dysmenorrhoea.	Mahwasane et al. (2013)	1
Ledebouria apertiflora (Baker) Jessop. Synonyms: *Drimia apertiflora* Baker, *Scilla linearifolia* Baker	Desert African hyacinth (E)	Asparagaceae	Herb	Bulbs	The bulb is crushed and rubbed on painful areas of the body	Mogale et al. (2019)	1
Ledebouria floribunda (Baker) Jessop. Synonyms: *Scilla floribunda* Baker, *Scilla princeps* Baker	spotted African hyacinth (E), Ikhambi lezingane, Iscociso, Umababaza, Umbola, Umbola wentaba (Z)	Asparagaceae	Herb	ns	Abnormal abdominal pains and abdominal swelling in infants	Mhlongo and Van Wyk (2019)	1
Ledebouria ovatifolia (Baker) Jessop. Synonyms: *Scilla cicatricosa*, *Scilla elevans*, *Scilla ovatifolia*	Flat-leaved African hyacinth (E), untanganazibomvu (X), Ikhambi lezingane, Imbiza yezingane, Intelezi, Umababaza (Z)	Asparagaceae	Herb	Bulbs	Bulbs are taken for backache. Abnormal abdominal pains and abdominal swelling	Hutchings et al. (1996); Mhlongo and Van Wyk (2019)	2

Botanical name	*Common name	Family	Life-form	#Part used	Application	Reference	Nm
Leonotis leonurus (L.) R.Br. Synonyms: *Hemisodon leonurus* (L.) Raf., *Phlomis leonurus* L.	Wild dagga, lion's ear, leonotis (E), wildedagga, duiwelstabak (A), imvovo, utywala-bengcungcu, umfincafincane, umunyamunya (X), umfincafincane, umcwili, utshwala-bezinyoni (Z)	Lamiaceae	Shrub	Leaves, flowers	Leaf decoction is taken orally to treat headache. Used to treat stomach ailments, backache, pain and inflammation. An infusion is made from a handful of leaves and flowers steeped in boiling water and left to draw in a glass bottle. About 25 ml is drunk morning and night for the arthritis, backache, headache and rheumatism	Bhat (2013); Hulley and Van Wyk (2019); Philander (2011); Thring and Weitz (2006)	4
Leonotis ocymifolia (Burm.f.) Iwarsson. Synonyms: *Leonotis capensis* Raf., *Phlomis ocymifolia* Burm.f.	Minaret flower, lion’s ear, rock lion's paw (E), Klipdagga, wildedagga (A), umcwili (Z)	Lamiaceae	Shrub	Leaves	Dried, powdered leaves used as a snuff for headache. Used to treat backache, pain and inflammation.	Van Wyk et al. (2008); Hulley and Van Wyk (2019)	2
Lepidium africanum (Burm.f.) DC. Synonyms: *Lepidium africanum subsp. divaricatum* (Aiton) Jonsell, *Lepidium tasmanicum* Thell.	Birdseed, Cape Pepper Cress, Peppergrass, Pepperweed, Pepperwort (E), Kanariesaadgras, Peperbossie (A), Sebista (SS)	Brassicaceae	Herb	Roots	Root is eaten to treat stomachache	Hulley and Van Wyk (2019)	1
Lessertia inflata Harv. Synonym: *Lessertia versicaria* E.Mey.	Seeroogbossie, lê-wildekeur (A)	Fabaceae	Herb	ns	For stomachache	Van Wyk et al. (2008)	1
Leucas lavandulifolia Sm. Synonyms: *Leonurus indicus* L., *Leucas linifolia* (Roth) Spreng. *Leucas indica* (L.) Vatke	Umagumede (Z)	Lamiaceae	Herb	ns	Analgesic (fever, back aches, headache)	Mhlongo and Van Wyk (2019)	1
Lichtensteinia lacera Cham. & Schltdl. Synonyms: *Lichtensteinia lacera* var. *lacera, Lichtensteinia lacera*	Larger tinsel flower (E), Kalmoes (A), iQwili (X)	Apiaceae	Herb	Whole plant	For relieving headache	Mintsa Mi Nzue (2009)	1

Botanical name	*Common name	Family	Life-form	#Part used	Application	Reference	Nm
Cham. & Schltdl. var. *pinnatifida* Sond.							
Limeum aethiopicum Burm. f. Synonyms: *Limeum aethiopicum* subsp. *aethiopicum*, *Limeum aethiopicum* var. *aethiopicum*	Boesmandagga, koggelmandervoet (A)	Molluginaceae (Limeaceae – World Flora Online)	Herb	ns	Infusion is used to treat headache	Hulley and Van Wyk (2019)	1
Linum thunbergii Eckl. & Zeyh.	Wild flax (E), Wildevlas (A), Bohlokoana (SS), ithalel impofu (Z)	Linaceae	Herb	Root	Root decoction used for relieving pain.	Watt and Breyer-Brandwijk (1962)	1
Lippia javanica (Burm.f.) Spreng. Synonyms: *Lantana galpiniana*, *Lippia asperifolia*, *Verbena javanica*	Fever tea, lemon bush, wild sage (E), koorsbossie, beukesbossie, lemoenbossie (A), musukudu, bokhukhwane (TW), inzinziniba (X); umsuzwane, umswazi (Z)	Verbenaceae	Tree	Leaves, roots	Leaves are used for headaches while roots are used for headaches and backache. Analgesic (headache), musculo-skeletal (inflammation)	Hutchings et al. (1996); Mhlongo and Van Wyk (2019)	2
Lobelia flaccida (C.Presl) A.DC. Synonyms: *Lobelia bellidifolia* f. *flexuosa* Zahlbr., *Rapuntium bellidifolium* var. *brevidens* C.Presl	Itshilizi (X)	Campanulaceae	Herb	Leaves	For abdominal pain	Maroyi (2017)	1
Lobostemon fruticosus (L.) H.Buek Synonyms: *Echium fruticosum* var. *minor* Sims, *Lobostemon obovatus* DC. & A.DC.	Pajama bush, 8-days (E), agtdaegeneesbos, lobos, douwurmbos, luibos (A)	Boraginaceae	Shrub	Leaves, whole plant	For treating stomachache and pains	Philander (2011); Mintsa Mi Nzue (2009)	2
Lopholaena coriifolia (Sond.) E.Phillips & C.A.Sm. Synonyms: *Lopholaena bainesii* (Oliv. & Hiern) S.Moore, *Othonna coriifolia* Sond.	Leather-leaved Fluff-bush (E), Pluisiesbos (A)	Asteraceae	Shrub	Branches	Dried branches are burned and the smoke is inhaled to treat headaches	Mogale et al. (2019)	1
Macrotyloma maranguense (Taub.) Verdc.	Xikondlo (Xitsonga)	Fabaceae	Herb	Bulbs	Bulb is chewed as remedy for swollen or painful testicles	Tshikalange et al. (2016)	1

Botanical name	*Common name	Family	Life-form	#Part used	Application	Reference	Nm
Maerua cafra (DC.) Pax. Synonyms: *Maerua triphylla* (Thunb.) T.Durand & Schinz, *Niebuhria triphylla* (Thunb.) H.L.Wendl.	Common bush-cherry, White-wood (E), Witbos (A), Gwambazi-Mukundulela (V)	Capparaceae	Tree	Root	Root decoction is used to prepare soft porridge to relieve headache due to indigestion. Roots are buned and smoke inhaled to relieve headache	Arnold and Gulumian (1984)	1
Maesa lanceolata Forssk. Synonyms: *Baeobotrys ovata* Willd. ex Schult., *Maesa trichophlebia* Baker	false assegai (E); valsassegaai (A), intendekwane (X), muunguri (Venda), Isidende (Z)	Maesaceae	Shrub	ns	Musculo-skeletal (inflammation)	Mhlongo and Van Wyk (2019)	1
Manilkara concolor (Harv.) Gerstner. Synonym: *Mimusops concolor*	Zulu milkberry (E), Zoeloemelkbessie (A), umncambu, umnqambo (Z)	Sapotaceae	Tree	Rootbark	Rootbark decoctions are administered as powerful enemas for back pain	Palmer and Pitman (1961)	1
Manilkara discolor (Sond.) J.H.Hemsl. Synonyms: *Eichleria discolour*, *Labourdonnaisia discolour*, *Mahea natalensis*, *Muriea discolour*	Forest milkberry, red milkwood (E), rooimelkhout, bosmelkbossie (A), umnqambo, unweba(-wentaba) (Z)	Sapotaceae	Tree	Rootbark	Rootbark decoctions are administered as powerful enemas for back pain and also for brittle bones	Palmer and Pitman (1961); Pooley (1993)	2
Manilkara mochisia (Baker) Dubard. Synonyms: *Manilkara macaulayae*, *Mimusops mochisia*	Lowveld milkberry (E), laeveldmelkbessie (A), Munamba (V), mvambu, umncambu (Z)	Sapotaceae	Tree	Roots	Root used in enemas for back pain	Palmer and Pitman (1961); Pooley (1993)	2
Maytenus peduncularis Loes. Synonyms: *Celastrus peduncularis* Sond., *Gymnosporia peduncularis* (Sond.) Loes.	Cape Blackwood, Indigenous Blackwood (E), Kaapse Swarthout (A), Makhulu-wa-mukwatule (V), Umnqai, Umnqayi, Umnqayinqayi, Umnqayi-nqayi (X), Inqayi Elimnyama (Z)	Celastraceae	Tree	Roots	Root decoction is drunk 4 times daily for backache in male	Arnold and Gulumian (1984)	1

Botanical name	*Common name	Family	Life-form	#Part used	Application	Reference	Nm
Melianthus comosus Vahl. Synonym: *Diplerisma comosum* (Vahl) Planch.	Touch-me-not, honey flower (E), Kruidjie-roer-my-nie (A), ibonya (Z)	Melianthaceae	Shrub	Leaves, whole plant	Plant decoctions are used to bathe rheumatic limbs and painful feet while leaf paste has also been used to reduce the swelling of bruises. Herb is applied topically for inflammation of legs. Boiled leaves is applied to painful knees. For treating rheumatism painful back and legs. Used as a wash for pain and inflammation, backache, rheumatism, wounds, sores and as a rinse for toothache and to pull teeth	Hutchings et al. (1996); Van Wyk et al. (2008); De Beer and Van Wyk (2011); Hulley and Van Wyk (2019)	4
Melianthus major L.	Honey flower, melianthus, giant honey flower, touch-me-not (E), kruidjie-roer-my-nie, heuningblom, klappers, krikkiebos (A), ubuhlungubemamba, ubutyayi (X), ibonya (Z)	Melianthaceae	Shrub	Leaves	External poultice for pain and rheumatism	Philander (2011)	1
Melianthus pectinatus Harv.	Honey flower, melianthus, giant honey flower, touch-me-not (E), kruidjie-roer-my-nie, heuningblom, klappers (A), Kriekiebos (KS), ubuhlungubemamba, ubutyayi (X), ibonya (Z)	Melianthaceae	Shrub	Leaves, roots	Lukewarm decoction of green leaves used as wash for painful legs. Leaves used for rheumatism. Root used as snuff with rooistorm, cloves and tobacco (boertwak) for headache	Nortje and van Wyk (2015)	1
Melolobium candicans (E.Mey.) Eckl. & Zeyh.	Wilde dagga; naeltjiedoring (A)	Fabaceae	Herb	Stem, leaves	Decoction of the stem and leaves used to treat stomach problems	De Beer and Van Wyk (2011)	1
Mentha longifolia (L.) Huds. Synonyms: *Mentha aepycaulos*	Wild mint (E), ballerja, balderjan, baldrian,t'kamma	Lamiaceae	Herb	Leaves	Leaves used as compress on pains and sores. Ointment	Nortje and van Wyk (2015); Van	5

Botanical name	*Common name	Family	Life-form	#Part used	Application	Reference	Nm
Candargy, *Mentha brassoensis* (Topitz) Trautm.	(A), Ballerja (KS), inixina, inzinziniba (X), ufuthana lomhlanga (Z)				used for painful legs. Prepared as infusion for treating headache and arthritis. Warm leaves used as a compress to treat headache and stomach pains, used for washing aching legs. Used to treat toothache, headache, ear-ache, pain and inflammation	Wyk et al. (2008); De Beer and Van Wyk (2011); Hulley and Van Wyk (2019); Thring and Weitz (2006)	
Mentha spicata L. Synonyms: *Mentha aquatica* var. *crispa* (L.) Benth., *Mentha crispa* L.	Spearmint, lamb mint (E), Kruistement (KS)	Lamiaceae	Herb	Leaves, ns	Infusion used for pains (inflammation) and menstrual pains. Leaf infusion is used as headache remedy	Nortje and van Wyk (2015); Van Wyk et al. (2008)	2
Merwilla plumbea (Lindl.) Speta. Synonyms: *Scilla natalensis*, *Scilla plumbea* Lindl.	Blue squill (E), blousalngkop (A), kherere (SS), ichitha, inguduza, ubulika (Z)	Asparagaceae	Herb	Bulb	Bulb decoctions are used for sprains and fractures. Used for treating chest pain	Watt and Breyer-Brandwijk (1962); Mbanjwa (2020)	2
Mesembryanthemum cordifolium L.f. Synonyms: *Aptenia cordifolia*, *Litocarpus cordifolius* (L.f.) L.Bolus	Red aptenia, baby sun rose (E), brakvygie (A), Ibohlololo, Umjuluka, Uncolozi Omncane (Z)	Aizoaceae	Herb	Leaves, stem	Burnt leaves and stems are applied to painful joints as anti-inflammatory	Hutchings et al. (1996); Mhlongo and Van Wyk (2019)	2
Microglossa mespilifolia (Less.) B.L. Rob. Synonyms: *Aster mespilifolius* Less., *Erigeron natalensis* Sch.Bip.	Ikhambi lesduli, Ikhambi elimhlophe, Umazambezi (Z)	Asteraceae	Herb	ns	Analgesic (back pains)	Mhlongo and Van Wyk (2019)	1
Mikania natalensis DC.	Mikania (E), Ihlozi, Ikhambi-lesiduli (Z)	Asteraceae	Climber	ns	Analgesic (headaches)	Mhlongo and Van Wyk (2019)	1
Millettia stuhlmannii Taub.	panga panga, partridge wood (E), panga-panga, patryshout (A), muangaila (V)	Fabaceae	Tree	Roots	Root decoction is drunk for 2 days to relieve stomachache	Arnold and Gulumian (1984)	1

Botanical name	*Common name	Family	Life-form	#Part used	Application	Reference	Nm
Mimusops zeyheri Sond. Synonyms: *Mimusops blantyreana* Engl., *Mimusops monroi* S.Moore	Transvaal red milkwood (E), moepel (A), mmupudu (NS), nhlantswa (Tsonga); mubululu (V), umpushane (Z)	Sapotaceae	Tree	Roots	Roots are used to treat stomachache	Mongalo and Makhafola (2018)	1
Muraltia spinosa (L.) F.Forest & J.C.Manning (=*Nylandtia spinosa* (L.) Dumort.)	Tortoise berry (E), skilpadbessie, duinebessie, bokbessie (A), mmaba (TW)	Polygalaceae	Shrub	ns	Used to treat stomach ailments	Hulley and Van Wyk (2019)	1
Musa × paradisiaca L. Synonyms: *Musa × paradisiaca* subsp. *sapientum* (L.) Kuntze, *Musa × acutibracteata* M.Hotta	Muomva (V)	Musaceae	Herb	Fruits	Fruit decoction is drunk for heart pains	Arnold and Gulumian (1984)	1
Nemesia fruticans Benth. Synonyms: *Nemesia capensis* Kuntze, *Nemesia gracillima* Dinter	Cape snapdragon, mauve nemesia (E), leeubekkie, maagpynblommetjie, witleeubekkie, wildeleeubekkie (A)	Scrophulariaceae	Herb	ns	Used to treat stomach ailments	Hulley and Van Wyk (2019)	1
Neorautanenia mitis (A.Rich.) Verdc. Synonyms: *Dolichos mitis* A. Rich., *Pachyrhizus orbicularis* Welw. ex Baker	Fig-leaf trailing pea (E), blou-ertjie, wilde-pronkertjie (A)	Fabaceae	Herb	Bulb	Bulbs are used to treat foot ache	Mongalo and Makhafola (2018)	1
Nerium oleander L. Synonyms: *Nerion oleandrum* St.-Lag., *Oleander indica* (Mill.) Medik.	Oleander, Rosebay, Five-roses (E)	Apocynaceae	Shrub	Leaves	Leaves are used to treat toothache	Mongalo and Makhafola (2018)	1
Nicotiana glauca Graham. Synonyms: *Nicotiana glauca* f. *lateritia* Lillo, *Nicotiana glauca* var. angustifolia Comes	Tree tobacco (E), wilde twak, jantwak(boom), jan twak (A), Jantwaks (KS), Icubamfene (X)	Solanaceae	Shrub	Leaves	Warmed leaves used as compress on pains. Leaves used as plug or as wash for earache. Used for headache. Fresh leaves is applied to the head as a poultice to draw out the pain. Dried leaves are used as fumitory to get rid of headache. Used as a	Nortje and van Wyk (2015); Maroyi (2017); Van Wyk et al. (2008); Bhat (2014); Hulley and Van Wyk (2019)	5

Botanical name	*Common name	Family	Life-form	#Part used	Application	Reference	Nm
					compress on head for headache, ear-ache, pain and inflammation		
Ochna natalitia (Meisn.) Walp.	Coastal- redwood, Natal plane (E), pronkrooihout, Natalrooihout (A), mbovu, isithundu (Z)	Ochnaceae	Tree	Roots	Root decoction is used for painful joints	Tshikalange et al. (2016)	1
Ocotea bullata (Burch.) E. Meyer in Drege. Synonyms: *Laurus bullata* Burch., *Oreodaphne bullata* (Burch.) Nees	Cape stinkwood, laurel wood (E), swartstinkhout (A), Umhlungulu, Umnimbithi (X), umnugani, umnukani (Z)	Lauraceae	Tree	Bark	Snuff or smoke from the bark is inhaled to treat headaches	Watt and Breyer-Brandwijk (1962); Coopoosamy and Naidoo (2012)	2
Olea capensis L. Synonyms: *Enaimon undulata* (Aiton) Raf., *Olea laurifolia* var. *concolor* (E.Mey.) Harv.	Small ironwood , black ironwood, buchuwood, buckutree, false ironwood (E), valsysterhout, witboekenhout (A), moraøane (NS), musiri (V), umnquma-swile (X), umsishane (Z)	Oleaceae	Tree	Roots	Powdered roots is used to cover incision on swollen joints	Arnold and Gulumian (1984)	1
Olea europaea subsp. *cuspidata* (Wall. & G.Don) Cif. Synonyms: *Olea europaea* subsp. *africana* (Mill.) P.S. Green, *Olea chrysophylla* var. *aucheri* A.Chev.	Wild olive (E), Olienhoutboom (KS), motlhware (TW), mutlhwari (V)	Oleaceae	Tree	Leaves	Leaf infusion used for backache. Leaves is used as a poultice for rheumatism	Nortje and van Wyk (2015); Hulley and Van Wyk (2019)	2
Oncosiphon piluliferum (L.f.) Källersjö. Synonyms: *Cenocline globifera* K.Koch, *Tanacetum obtusum* Thunb.	Gansogie, Karoostinkkruid, Knoppies-stinkkruid, Stinkkruid (A)	Asteraceae	Herb	Leaves	Compress leaves on head for headache. Infusion used to treat backache, pain and inflammation	Hulley and Van Wyk (2019)	1
Oncosiphon suffruticosum (L.) Källersjö. Synonyms: *Cotula tanacetifolia* L., *Pentzia tanacetifolia* (L.) Hutch.	Stinkingweed, Calomba daisy (E), stinkkruid, wurmkruid, wurmbos,	Asteraceae	Herb	Leaves	Leaves used as compress on pains. Used to treat stomach problems	Nortje and van Wyk (2015); De Beer and Van Wyk (2011)	2

Botanical name	*Common name	Family	Life-form	#Part used	Application	Reference	Nm
	miskruid (A), Stinkkruid (KS)						
Opuntia ficus-indica (L.) Mill. Synonyms: *Cactus ficus-indica* L., *Opuntia megacantha* Salm-Dyck	prickly pear (E), turksvy (A)	Cactaceae	Shrub	Leavees	Compress on knee for pain and on back for backache	Hulley and Van Wyk (2019)	1
Osmitopsis asteriscoides Less. Synonyms: *Leucanthemum asteriscoides* (L.) Kuntze, *Osmitopsis asteriscoides* Cass.	Swamp daisy, mountain daisy, bellis (E), bels, belskruie, belsbos, belsebos, belsbossie, belsebossie (A)	Asteraceae	Shrub	Leaves	Used for body pain	Philander (2011)	1
Osteospermum calendulaceum L.f. Synonyms: *Calendula parviflora* Thunb., *Oligocarpus calendulaceus* (L.f.) Less.	Boegoebos, Boegoebossie, geneesbos(sie) (A)	Asteraceae	Herb	Leaves	Infusions used for pain and inflammation. Used for stomach ailments	Hulley and Van Wyk (2019)	1
Osteospermum herbaceum L.f. Synonym: *Osteospermum zeyheri* Spreng. ex DC.	Stinktontel (A)	Asteraceae	Herb	Leaves	The leaf sap is mixed with powdered tea (black tea) and apply to the mouth/gum for treating toothache	Van Wyk et al. (2008)	1
Otholobium arborescens C.H. Stirt.	Renosterveld Hook-leaved Pea (E), Renosterveld-vlieëkeurtjie (A)	Fabaceae	Shrub	Twigs	Leafy twigs used for treating backache and headache	De Beer and Van Wyk (2011)	1
Ozoroa paniculosa (Sond.) R.Fern. & A.Fern. Synonyms: *Heeria paniculosa* (Sond.) Kuntze, *Rhus paniculosa* Sond.	common resin tree, bushveld ozoroa (E), gewone harpuisboom (A), isifice (Z), monoko (NS), mudumbula (V)	Anacardiaceae	Tree	Bark	The bark can be ground to a fine powder which is used to treat stomach pains	Mogale et al. (2019)	1
Parinari curatellifolia Planch. ex Benth. Synonyms: *Ferolia curatellifolia* (Planch. ex Benth.) Kuntze, *Parinari gardineri* Hemsl.	mobola-plum, cork tree, hissing tree (E), grysappel, bosappel (A), mmola (NS), mobola (TW), muvhula (V)	Chrysobalanaceae	Tree	Bark	Bark decoction is used to rinse oral cavity to relieve toothache. Bark decoction is used as nose and ear drops to relieve toothache die to mandibular and maxillar pains	Arnold and Gulumian (1984)	1

Botanical name	*Common name	Family	Life-form	#Part used	Application	Reference	Nm
Passiflora suberosa L. Synonyms: *Anthactinia walkeri* M.Roem., *Cieca hederacea* M.Roem.	Unyawo lenkukhu, Inhlanhla emhlophe (Z)	Passifloraceae	Climber	ns	Analgesic (lower back pains)	Mhlongo and Van Wyk (2019)	1
Pavetta graciliflora Wall. ex Ridl.	Ncolovoti (Xitsonga)	Rubiaceae	Herb	Root	Root decoction is used for treating painful feet	Tshikalange et al. (2016)	1
Pegolettia baccharidifolia Less. Synonyms: *Carphopappus baccharidifolius* (Less.) Sch.Bip., *Iphiona baccharidifolia* (Less.) Benth. & Hook.f. ex Dinter	Heuningbos, ghwarrieson, heuningdou (A)	Asteraceae	Herb	ns	For inflammation (on parts of the body or chest). For treating headache, backache, pain and inflammation	Van Wyk et al. (2008); Hulley and Van Wyk (2019)	2
Pelargonium grossularioides (L.) L'Hér. Synonym: *Geranium grossularioides* L.	gooseberry-leaved pelargonium (E), rooirabas, rooirabassam, rooistingelhoutbas (A)	Geraniaceae	Herb	ns	For treating backache and stomachache	Hulley and Van Wyk (2019)	1
Pelargonium hypoleucum Turcz. Synonym: *Pelargonium harveyanum* R.Knuth	Rooirabas (KS)	Geraniaceae	Herb	Roots	Root decoction used for backache and menstrual pains	Nortje and van Wyk (2015)	1
Pelargonium triste (L.) L'Hér. Synonyms: *Geraniospermum triste* (L.) Kuntze, *Pelargonium flavum* L. Ait.	Night-scented pelargonium, Sad geranium (E), Kaneelbol (A)	Geraniaceae	Herb	Corms, roots	For treatment of pains	Mintsa Mi Nzue (2009)	1
Pelargonium peltatum (L.) L'Hér. Synonyms: *Pelargonium lateripes* L'Hér., *Dibrachya clypeata* Eckl. & Zeyh.	ivy-leaved pelargonium, ivy pelargonium, wild sorrel, cascading pelargonium (E), kolsuring, wildemalva (A), umnewana, ityholo (X)	Geraniaceae	Herb	ns	Used for the treatment of ear-ache	Hulley and Van Wyk (2019)	1
Pelargonium ramosissimum Willd.	Dassieboegoe, Dassiebuchu, dassiebos (A)	Geraniaceae	Shrub	Leaves, stem	Infusion of leafy stem used to treat backache and stomach problems	De Beer and Van Wyk (2011)	1

Botanical name	*Common name	Family	Life-form	#Part used	Application	Reference	Nm
Pelargonium zonale (L.) L'Hér. ex Aiton. Synonyms: *Ciconium clarum* Hoffmanns., *Ciconium stenopetalum* Hoffmanns.	horse-shoe pelargonium (E), wildemalva, bergmalva (A)	Geraniaceae	Shrub	Leaves	Fresh leaves are used as ear plugs for earche. For treating toothache and ear-ache	Van Wyk et al. (2008); Hulley and Van Wyk (2019)	2
Peltophorum africanum Sond. Synonym: *Brasilettia africana*	Weeping wattle, Natal wattle (E), huilboom, kiaatboom (A), Mosêhla, Mosese (NS), Mosêtlha (TW), umsehle, Isikhabamkhombe (Z)	Fabaceae	Tree	Roots, bark	Roots and bark are used for backache. Bark is also used for abdominal pain while leaves are used for toothache and abdominal pain. Root decoction is used for body pain	Pooley (1993); Mabogo (1990); Watt and Breyer-Brandwijk (1962); Mogale et al. (2019); Tshikalange et al. (2016)	5
Pentanisia prunelloides subsp. *latifolia* (Hochst.) Verdc. Synonyms: *Pentanisia variabilis* Harv. var. *latifolia*, *Declieuxia latifolia* Hochst.	Wild verbena, broad-leaved Pentanisia (E), sooibrandbossie (A), isigcikamlilo (X), Icishamlilo, Icishamlilo elikhulu (Z)	Rubiaceae	Herb	Leaves, roots	Decoctions are sprinkled on painful parts for treating rheumatism. Pounded roots are applied to burns and used in poultices for inflammation and swollen joints. Leaf poultices or hot root decoctions are applied to painful swellings, rheumatic parts, sprains and sores. Analgesic (general body pains). Root decoction is used for treating rheumatism	Bryant (1966); Gerstner (1941); Hulme (1954); Watt and Breyer-Brandwijk (1962); Mhlongo and Van Wyk (2019); Bhat (2014); Mintsa Mi Nzue (2009); Mbanjwa (2020)	8
Pentzia dentata (L.) Kuntze. Synonyms: *Athanasia flabellifera* Salisb., *Pentzia flabelliformis* Willd.	greater skaapkaroo, duck's-foot skaapkaroo (E), grootskaapkaroo, eendepootkaroo (A)	Asteraceae	Shrub		Used for relieving menstration pains	Hulley and Van Wyk (2019)	1
Pentzia incana (Thunb.) Kuntze. Synonyms: *Chrysanthemum incanum* Thunb., *Pentzia virgata* Less.	Anchor Karoo, Common Karro (E), Ankerkaroo, Gansie, Alsbossie, Rooikarobos (A), Mohantsoana (SS)	Asteraceae	Shrub	Leaves, twigs, ns	For stomachache. Leaves are chewed to treat stomach cramps and to treat general pain. Twigs are chewed to extract juices for treating	Van Wyk et al. (2008); De Beer and Van Wyk (2011); Hulley	3

Botanical name	*Common name	Family	Life-form	#Part used	Application	Reference	Nm
					stomachache, backache, pain and inflammation	and Van Wyk (2019)	
Petroselinum crispum (Mill.) Fuss. Synonyms: *Apium petroselinum* var. *angustifolium* Hayne, *Wydleria portoricensis* DC.	Parsley (E), pietersielie (A)	Apiaceae	Herb	Leaves	Taken as an infusion made from a handful of leaves taken when needed for treating arthritis and rheumatism	Thring and Weitz (2006)	1
Phoenix reclinata Jacq. Synonyms: *Fulchironia senegalensis* Lesch., *Phoenix dybowskii* A.Chev.	Wild date palm (E), Isundu, Usundu (Z)	Arecaceae	Tree	ns	Analgesic (sharp internal body pains, internal side pains, toothache)	Mhlongo and Van Wyk (2019)	1
Phragmites australis (Cav.) Trin. ex Steud. Synonyms: *Phragmites communis* Trin., *Remirea diffusa* Sieber ex Steud.	Common reed (E)	Poaceae	Grass	Seeds	Used to cover incisions made on swollen joints after the application of *Olea capensis* L.	Arnold and Gulumian (1984)	1
Phytolacca americana L. Synonyms: *Phytolacca americana* var. *americana*, *Phytolacca decandra* L.	Cancer jalap/root, inkberry, poke berry/root/weed (E), karmosynbos (A), umnanja, umnyanja (Z)	Phytolaccaceae	Herb	Fruit, roots	Fruit and roots are used as anti-rheumatics, anti-inflammatories for pain	Hutchings et al. (1996)	1
Pittosporum viridiflorum Sims. Synonyms: *Pittosporum abyssinicum*, *Pittosporum antunesii*, *Pittosporum commutatum*, *Pittosporum floribundum*	Cheesewood (E), bosbeukenhout, Kasuur (A), Kgalagangwe (NS), Mosetlela (SS), Mutanzwakhamelo (V), umkhwenkwe (X), umfusamvu, umkwenkwe (Z)	Pittosporaceae	Shrub	Bark, roots	Bark decoctions are also taken for pains in the back as emetics or enemas for stomach troubles particularly those to ease pain. Root infusions are taken for chest pains. Taken for abdominal pain	Watt and Breyer-Brandwijk (1962); Maroyi (2017); Van Wyk et al. (2008)	3
Ptaeroxylon obliquum (Thunb.) Radlk. Synonyms: *Ptaeroxylon utile* Eckl. & Zeyh, *Rhus obliqua* Thunb.	Sneezewood (E), umThathi (X), umthathe (Z)	Rutaceae	Tree	Bark	Treating headache	Philander (2011)	1
Plantago lanceolata L. Synonyms: *Arnoglossum*	Buckhorn Plantain, English Plantain, German Psyllium, Lamb's Tongue (E),	Plantaginaceae	Herb	Leaves, roots	Small leaves are inserted in the nostrils to heal headache. Root juice is used to treat	Bhat (2014); Hulley and Van Wyk (2019)	2

Botanical name	*Common name	Family	Life-form	#Part used	Application	Reference	Nm
lanceolatum (L.) Gray, *Plantago sinuata* Lam.	Msalweebree, Msalweegbree (A), Setla-bocha, Bolila-nyana (SS), Indlebe-kathekwane Encane (Z)				ear-ache. Leaves are used as compress on aching feet. Infusions are used for stomach ailments		
Platycarpha glomerata (Thunb.) Less. Synonyms: *Cynara glomerata* Thunb., *Stobaea glomerata* (Thunb.) Spreng.	Imbozisa, Imbozisa encane, Isiphahluka, Ubani, Ubani olukhulu, Ukhula, Umabopha,Umbola, Umkhwibi ompofu (Z)	Asteraceae	Herb	Roots, ns	Internal side pain in children, chest pain and musculo-skeletal (inflammation). Yellow sap is used for cleaning ear or ear-ache	Mhlongo and Van Wyk (2019); Philander (2011); Mbanjwa (2020)	3
Plumbago auriculata Lam. Synonyms: *Plumbagidium auriculatum* (Lam.) Spach, *Plumbago capensis*	Cape leadwort, plumbago (E), syselbos (A), Umabophe, umatshintshine, (X), umasheleshele, Ubani, umaswelisweli (Z)	Plumbaginaceae	Shrub	Roots, leaves	Powdered roots or dried leaves are taken as snuff to relieve headaches. Analgesic (sharp internal body pains)	Gerstner (1941); Hutchings et al. (1996); Mhlongo and Van Wyk (2019)	3
Portulaca oleracea L. Synonyms: *Portulaca consanguinea* Schltdl., *Portulaca sylvestris* Montandon	Common Purslane (E), porstelein, reslein, oumisbredie (A)	Portulacaceae	Herb	ns	Used to treat backache	Hulley and Van Wyk (2019)	1
Portulacaria afra Jacq.	Porkbush, Elephant's Food (E), Spekboom. Olifantskos, spekboomblare (A), iNtelezi, isiDondwane, isAmbilane, iNdibili, isiCococo (Z), iGqwanitsha (X)	Portulacaceae	Shrub	ns	Eaten as treatment for stomachache, ear-ache, pain and inflammation	Hulley and Van Wyk (2019)	1
Protorhus longifolia (Bernh.) Engl. Synonyms: *Anaphrenium longifolium* Bernh., *Rhus longifolia* (Bernh.) Sond.	Purple currant, red beech, red cape beech (E), Harpuisboom (A), Ikhubalo, Isifuce(X), Mutu-musolde (V), Isifice, Isifico-sehlathi, Isifuze (Z),	Anacardiaceae	Tree	Bark	Bark decoctions are taken in 200 ml doses as emetics to relieve heartburn and bleeding from the stomach. Smoked to relieve headache	Pujol (1990); Mbanjwa (2020)	2

Botanical name	*Common name	Family	Life-form	#Part used	Application	Reference	Nm
Prunus africana (Hook.f.) Kalkman. Synonym: *Pygeum africanum* Hook. f.	African almond tree, bitter almond (E), Bitter-amandel, Bitteramandelboom (A), Mogohloro (NS), Umkhakhase, Itywina-elikhul (X), Mulala-maanga (V), umdumizula, umkhakhazi (Z)	Rosaceae	Tree	Bark	Bark is used to treat intercostal pain and vhest pain	Pujol (1990); Coopoosamy and Naidoo (2012)	2
Prunus persica (L.) Batsch. Synonyms: *Persica vulgaris* var. *compressa* Loudon, *Prunus persica* f. *scleropersica* (Rchb.) Voss	Umpetshisi, Ipentshisi (Z)	Rosaceae	Tree	ns	For relieving menstrual pain	Mbanjwa (2020)	1
Pseudophyllanthus ovalis (E.Mey. ex Sond.) Voronts. & Petra Hoffm. Synonyms: *Andrachne ovalis* (E.Mey. ex Sond.) Müll.Arg., *Phyllanthus ovalis* E.Mey. ex Sond.	False Lightening Bush (E), Valsbliksembos (A), umgqata (X), umbheza (Z)	Phyllanthaceae	Shrub	Roots	For body pains and aches	Philander (2011)	1
Psiadia punctulata (DC.) Vatke. Synonyms: *Baccharis resiniflua* Steud. & Hochst. ex DC., *Psiadia arabica* Jaub. & Spach	Sticky Psiadia (E), Blink Stefaans (A)	Asteraceae	Shrub	ns	Plant is used to rinse painful eyes	Mogale et al. (2019)	1
Psidium guajava L. Synonyms: *Guajava pumila* (Vahl) Kuntze, *Syzygium ellipticum* K.Schum. & Lauterb.	Guava (E)	Myrtaceae	Shrub	Roots	Roots are used for stomachache. Leaf infusion is used to treat arthiritis	Mongalo and Makhafola (2018); Hulley and Van Wyk (2019)	2
Ptaeroxylon obliquum (Thunb.) Radlk. Synonyms: *Ptaeroxylon utile* Eckl. & Zeyh, *Rhus obliqua* Thunb.	Sneezewood, stinkhout (E), Nieshout (A), Mulari, Munari, Munukha-vhaloi (V), Umthathi, Umthote	Rutaceae	Tree	Bark	Bark infusions are use against rheumatism and arthritis. Powdered bark is use to relieve headaches	Pujol (1990); Watt and Breyer-Brandwijk (1962); Mhlongo	3

Botanical name	*Common name	Family	Life-form	#Part used	Application	Reference	Nm
	(X), Ithatha, Umthathi, Umzane, Uthathi (Z)					and Van Wyk (2019)	
Pterocarpus angolensis DC. Synonyms: *Pterocarpus bussei* Harms, *Pterocarpus dekindtianus* var. *latifoliolatus* De Wild.	Bloodwood, paddle-wood, sealing-wax tree, wild teak, Transvaal teak (E), bloedhout, kajatenhout, lakhout, wilde-kiaat (A), morôtô (NS), mokwa, morotômadi (TW); mutondo (V), umvangazi, umbilo (Z)	Fabaceae	Tree	Roots	Roots are macerated and taken for headache	Arnold and Gulumian (1984)	1
Pterodiscus kellerianus Schinz	Moyane (NS)	Pedaliaceae	Herb	Roots	Fleshy roots are used to treat stomach aches in new-born babies	Mongalo and Makhafola (2018)	1
Pteronia camphorata (L.) L. Synonyms: *Pteronia aspera* Thunb., *Pteronia laricina* Houtt. ex DC.	sand gumbush (E), sandgombos (A), Wakkerbos (KS)	Asteraceae	Shrub	Leaves, twigs	Powdered leaves used medicinally as snuff (mixed with other herbs). Leaves and twigs infused in milk or water used to alleviate toothache. Dry powdered leaves applied on cotton wool to relief of earache. infusion used for rheumatism	Nortje and van Wyk (2015)	1
Pteronia cinerea L.f. Synonym: *Pteronia canescens* DC.	Boegoe (KS)	Asteraceae	Shrub	Leaves	leaf infusion used for low back pain	Nortje and van Wyk (2015)	1
Pteronia incana (Burm.) DC. Synonyms: *Athanasia rigida* Scop., *Pteronia xantholepis* DC.	skieterbosa, keurtjiebosa, kraakbos (A)	Asteraceae	Herb	ns	Infusion is used for backache and stomachache	Hulley and Van Wyk (2019)	1
Punica granatum L. Synonyms: *Punica nana* L., *Punica spinosa* Lam.	Granaat, grinaat, wildegranaatbos (A), garenate (SS)	Lythraceae	Shrub	Fruits	Used for treating stomachache. The skin of the fruit is used to treat stomach pains	Hulley and Van Wyk (2019); Mogale et al. (2019)	2

Botanical name	*Common name	Family	Life-form	#Part used	Application	Reference	Nm
Radyera urens (L.f.) Bullock. Synonyms: *Allenia urena* (L.f.) E.Phillips, *Hibiscus urens* L. f.	Jeukblom, Jigblom, Pampoenbossie (A), Wildepampoen (KS)	Malvaceae	Herb	Leaves	leaves as compress on pains	Nortje and van Wyk (2015)	1
Rapanea melanophloeos (L.) Mez. Synonyms: *Heeria melanophloeos* (L.) Meisn., *Myrsine melanophloeos*	Cape beech tree (E), boekenhout, rooiboekenhout (A), Isiqwane Sehlathi (X), Mogônô (NS), Tshididiri, Tshikonwa (V), Isihluthi-wentaba, ikhubalwane (Z)	Primulaceae	Tree	Bark	Bark is used for stomach and muscular pain. Ground bark decoctions are taken for stomach ache	Gerstner (1941); Hutchings et al. (1996); Pujol (1990)	3
Raphionacme procumbens Schltr.	Dema (Xitsonga)	Apocynaceae	Herb	Bulb	Bulb is mixed with milk and used for painful waist	Tshikalange et al. (2016)	1
Rauvolfia caffra Sond. Synonyms: *Rauvolfia inebrians* K.Schum., *Rauvolfia oxyphylla* Stapf	quinine tree (E), kinaboom (A), umJelo, umThundisa (X), Umhlambamanzi, umKhadluvungu (Z)	Apocynaceae	Tree	ns	Analgesic (toothache)	Mhlongo and Van Wyk (2019)	1
Rhoicissus digitata (L. f.) Gilg & M. Brandt. Synonym: *Rhus digitatum* L. f.	Baboon grape (E), bobbejaandruif (A), isaQoni, isaQoni esincini, Uchithibhunga (X), isiNwazi, umThwazi, umNangwazi, umPhambane (Z);	Vitaceae	Climber	Roots	For treating headache	Maroyi (2017)	1
Rhoicissus tomentosa (Lam.) Wild & R.B. Drumm. Synonym: *Cissus tomentosa* Lam.	Wild grape, bush grape, African grape, forest Grape, monkey rope (E), Bosdruif, Wildedruif, Bobbejaantou (A), isaQoni, iDiliya (X), Makhulu-wa-khundwi (V), Isende lengulube, Isinwazi (Z)	Vitaceae	Climber	Leaves, roots, stem, ns	Analgesic (chronic headache). Remedy for pains	Mhlongo and Van Wyk (2019); Mintsa Mi Nzue (2009)	2
Rhoicissus tridentata (L.f.) Wild & R.B.Drumm. Synonyms: *Rhus tridentata* L.f.,	Northern Bushman's grape, bitter grape, wild grape (E), noordelike boesmansdruif,	Vitaceae	Tree	Roots	Powdered roots is added to porridge to relieve stomach pain in children	Arnold and Gulumian (1984)	1

Botanical name	*Common name	Family	Life-form	#Part used	Application	Reference	Nm
Toxicodendron tridentatum (L.f.) Kuntze	bitterdruif, droog-my-keel, wildedruif (A), isaqoni, umnxeba, ulatile, uchithibhunga (X), murumbula-mbudzana (V), isinwazi, umthwazi (Z)						
Rhus chirindensis Bak. f. Synonyms: *Searsia chirindensis*, *Rhus chirendensis* A. Fern. & R. Fern., *Rhus legatii* Schönland	Red currant (E), Bostaaibos (A), umhlakothi (X), muvhadela-phanga (V), Inhlakoshane, Inyazangoma, Inyazangoma elimhlophe (Z)	Anacardiaceae	Tree	Bark	Bark is used to strengthen the body and for rheumatism. Analgesic (*isincindo* for pains, sharp internal body pains)	Pujol (1990); Mhlongo and Van Wyk (2019)	2
Ricinus communis L. Synonyms: *Ricinus africanus* Mill., *Ricinus communis* var. *communis*	Castor bean (E), kasterolie (boom) (A), Olieboom, kasterolie (KS), Mupfure (V), Umkakuva, umhlakuva (X), Uhlakuva, Umhlakuva (Z)	Euphorbiaceae	Shrub	Leaves, seeds, fruits	Analgesic (toothache), musculo-skeletal (inflammation). Oil from ground seeds used as ointment and leaves as compress on pains and rheumatism. Leaves compress on the cheek for toothache. Treating stomachache. Leaves are heated and placed on painful knees/joints. Oil squeezed from the fruits into the ear to relieve ear-ache. Fresh leaves are ground and mixed with water and about 50 ml is given orally to treat stomachache. Compress leaveas for headache, pain and inflammation as well as sprains. Warm leaves is	Mhlongo and Van Wyk (2019); Nortje and van Wyk (2015); Maroyi (2017); De Beer and Van Wyk (2011); Arnold and Gulumian (1984); Bhat (2014); Hulley and Van Wyk (2019), Philander (2011); Thring and Weitz (2006); Mbanjwa (2020)	10

Botanical name	*Common name	Family	Life-form	#Part used	Application	Reference	Nm
					wrapped around a child for stomachache		
Roepera morgsana (L.) Beier & Thulin. Synonym: *Zygophyllum morgsana* L.	Leeutjies (A)	Zygophyllaceae	Shrub	Roots	Roots are used for treating backache	Hulley and Van Wyk (2019)	1
Rosmarinus officinalis L. Synonyms: *Rosmarinus laxiflorus* Noë, *Rosmarinus rigidus* Jord. & Fourr.	Rosemary	Lamiaceae	Shrub	Leaves	For treating headache	Philander (2011)	1
Rosenia humilis (Less.) K.Bremer. Synonyms: *Nestlera conferta* DC., *Rosenia nestleroides* Compton	Hartebeeskaroo, Perdekaroo (A)	Asteraceae	Shrub	ns	For relieveing labour pains	Hulley and Van Wyk (2019)	1
Rothmannia capensis Thunb. Synonyms: *Gardenia capensis* (Thunb.) Druce, *Gardenia rothmannia* L.f.	Wild gardenia, common Rothmannia (E), iBolo (X), wildekatjiepiering (A), modulatshwene (NS), mukubudu (V), umPhazane-mkhlu (Z)	Rubiaceae	Tree	Roots	Powdered roots rubbed for scarification of rheumatism	Pooley (1993); Watt and Breyer-Brandwijk (1962)	2
Rubus ludwigii Eckl. & Zeyh. Synonyms: *Rubus rhodacantha* E.Mey., *Rubus ludwigii* subsp. *ludwigii*	Silver bramble, Wild Rasberry (E), Braam, Braambos (A), Monoko-metsi (SS), Itshalo, Imencemence, Unomhloshane (Z)	Rosaceae	Shrub	Roots	Powdered roots are taken in water to ease stomach ache. The Sotho administer decoctions for acute pain during illnesses	Watt and Breyer-Brandwijk (1962); Hutchings et al. (1996)	2
Rubus pinnatus Willd. Synonyms: *Rubus kingaensis* Engl., *Rubus pinnatus* var. *defensus* Gust.	South African blackberry (bramble or Raspberry), Capebramble (E), braambos braamboswortel (A), Iqunube (X), Ijingijolo, Mfongosi (Z)	Rosaceae	Shrub	Roots	Root decoctions are taken for various respiratory ailments including pain in the chest. Roots are used for toothache either as warm water gargles or ground and inserted directly into the cavity	Hutchings et al. (1996); Pujol (1990); Watt and Breyer-Brandwijk (1962)	3
Rubus rigidus Sm. Synonyms: *Dyctisperma rigidus* (Sm.) Raf.	White bramble (E), braambossie, braambos	Rosaceae	Shrub	Roots	Root decoctions are taken as gargles for toothache. Root	Hutchings et al. (1996); Watt and	4

Botanical name	*Common name	Family	Life-form	#Part used	Application	Reference	Nm
ex B.D.Jacks., *Rubus inedulis* Rolfe	(A), Umgcunube (X), ijingijolo, Amajikijolo, Amabhimbi (Z)				decoctions for acute pain during illnesses. Analgesic (toothache, sharp internal body pains)	Breyer-Brandwijk (1962); Mhlongo and Van Wyk (2019); Mbanjwa (2020)	
Rumex cordatus Desf. Synonym: *Rumex sarcorhizus* Link	Sorrel, Dock (E), Maksuring (A), Tongblaar (KS)	Polygonaceae	Herb	Leaves	warm leaves used as compress on pains, inflamed joints (e.g. a stiff neck)	Nortje and van Wyk (2015)	1
Rumex crispus L. Synonyms: *Lapathum crispum* (L.) Scop., *Rumex odontocarpus* Sandor ex Borbás	Tongblaar, beestong (A)	Polygonaceae	Herb	Leaves	Leaves used as compress on sores, pain and inflammation	Hulley and Van Wyk (2019)	1
Ruta graveolens L. Synonym: *Ruta hortensis* Mill.	Rue, Common rue, Herb of Grace, Garden Rue (E), wynruit, wynruik (A), Wynruit (KS)	Rutaceae	Shrub	Leaves, stem	Leaf infusion used for menstruation pains and toothache. As compress for low back pain and other pains. Leaf infusion to relief stomachache and treatment of headache. Used for inflammation and ear-ache	Nortje and van Wyk (2015); Van Wyk et al. (2008); De Beer and Van Wyk (2011); Hulley and Van Wyk (2019); Philander (2011); Thring and Weitz (2006); Mintsa Mi Nzue (2009)	7
Salix mucronata Thunb. Synonyms: *Salix capensis* var. *mucronata Anders*, *Salix safsaf* Forssk. ex Trautv.	Wild willow (E), Treurwilg Wildewilgerboom (A), Munengeledzi (V), Wilgerboom (KS)	Salicaceae	Tree	Branch tips, leaves, bark	Indicated as remedy against rheumatism. Leaf infusions used for backache. Leaves are used to treat pain. Infusions of the bark is ised to treat rheumatism, pain and inflammation.	Van Wyk and Gericke (2000); Nortje and van Wyk (2015); De Beer and Van Wyk (2011); Hulley and Van Wyk (2019)	4
Salvia africana L. = *Salvia africana-caerulea* L. Synonyms:	Blue African sage, blue sage, wild sage, African sage, purple sage (E),	Lamiaceae	Shrub	Leaves	For treating menstrual complaints	Philander (2011)	1

Botanical name	*Common name	Family	Life-form	#Part used	Application	Reference	Nm
Salvia colorata Vahl, *Stiefia molucellae* (Benth.) Soják	blousalie, bloublomsalie, perdesalie, wildesalie (A.)						
Salvia aurea L. = *Salvia africana-lutea* L. Synonyms: *Crolocos aurea* (L.) Raf., *Salvia eckloniana* Benth.	Golden sage, beach sage, dune sage, sand sage (E), bruinsalie, sandsalie, strandsalie, geelblomsalie (A)	Lamiaceae	Shrub	Leaves	Externally used for swelling and sprains	Philander (2011)	1
Salvia chamelaeagnea Berg. Synonyms: *Salvia chamelaeagnus* Burm.f., *Salvia paniculata* L.	Rough Blue Sage (E), Bloublomsalie (A)	Lamiaceae	Shrub	ns	Infusions are used to treat stomachache, pain and inflammation	Hulley and Van Wyk (2019)	1
Salvia dentata Aiton	Toothed sage (E), Bloublomsalie, bergsalie (KS)	Lamiaceae	Shrub	Leaves	Leaves used for pains. Leaf decoctions are used for backache	Nortje and van Wyk (2015); De Beer and Van Wyk (2011)	2
Salvia microphylla Kunth. Synonyms: *Salvia gasterantha* Briq., *Salvia odoratissima* Sessé & Moc.	baby sage, Graham's sage, or blackcurrant sage (E), rooisalie, rooiblomsalie (A)	Lamiaceae	Shrub	ns	Infusions are used for treating backache	Hulley and Van Wyk (2019)	1
Sansevieria hyacinthoides (L.) Druce. Synonyms: *Acyntha guineensis* (L.) Medik., *Salmia guineensis* (L.) Cav.	Mother-in-law’s tongue, piles root, bowstring hemp (E), isiKholokotho, Iskhothakhotha, Isqunga sehlathi (Z)	Ruscaceae (Asparagaceae – World Flora Online)	Herb	Roots, ns	Analgesic (toothache). The root is boiled in water and the water is gargled to treat toothache	Mhlongo and Van Wyk (2019); Corrigan et al. (2011)	2
Scabiosa columbaria L. Synonyms: *Asterocephalus columbaria* (L.) Wallr., *Scabiosa anthemifolia* Eckl. & Zeyh.	wild scabious (E), Upelepelane (Z)	Dipsacaceae (Caprifoliaceae - World Flora Online)	Herb	ns	Musculo-skeletal (lower back problems)	Mhlongo and Van Wyk (2019)	1
Scadoxus puniceus (L.) Friis & Nordal. Synonyms: *Haemanthus fax-imperii* Cufod., *Haemanthus redouteanus* M.Roem.	Paintbrush lily, Snake lily (E), Idumbe likahloyile (Z)	Amaryllidaceae	Herb	ns	Analgesic (fever, toothache)	Mhlongo and Van Wyk (2019)	1

Botanical name	*Common name	Family	Life-form	#Part used	Application	Reference	Nm
Sceletium emarcidum L. Bolus. Synonyms: *Mesembryanthemum anatomicum* Haw., *Mesembryanthemum emarcidum* Thunb.	kanna(wortel), Kougoed (A)	Aizoaceae	Herb	Leaves, whole plants	Inflammation. Whole plant is used for washing aching legs and treatment of toothache	Van Wyk et al. (2008); De Beer and Van Wyk (2011)	2
Sceletium tortuosum (L.) N.E.Br. Synonym: *Mesembryanthemum tortuosum* L.	kanna, tortuose fig marigold (E), Kougoed, springbok-slaai (KS)	Aizoaceae	Creeper	Leaves	Leaves are chewed and used for stomach ache, headache and toothache	Nortje and van Wyk (2015); Hulley and Van Wyk (2019)	2
Schinus molle L. Synonyms: *Schinus angustifolia* Sessé & Moc., *Schinus occidentalis* Sessé & Moc.	Peruvian pepper, pepper tree (E), peperboom (KS), peperboom (A)	Anacardiaceae	Tree	Leaves	Leaves as compress on painful legs, backache, headache and knee. Warm compress of leaves is placed on cheek for toothache. Vapour of leaf decoction is inhaled for inflammation and rheumatism. Leaf decoction is used to gargle to cure toothache. Compress for headache, pain and inflammation. Infusions for headache, pain and inflammation	Nortje and van Wyk (2015); Van Wyk et al. (2008); Bhat and Jacobs (1995); Bhat (2014); Hulley and Van Wyk (2019)	5
Schizocarphus nervosus (Burch.) van der Merwe. Synonyms: *Scilla nervosa* (Burch.) J.P.Jessop, *Drimia dregeana* Kunth	White Scilla (E), Sandlelie (A), Magaqana (X), ngcino (Z)	Asparagaceae	Herb	Bulbs	Diluted bulb decoctions are used in small doses for pain in rheumatic fever, taken for one day only	Bryant (1966); Watt and Breyer-Brandwijk (1962)	2
Schkuhria pinnata (Lam.) Kuntze ex Thell. Synonyms: *Amblyopappus mendocinus* Phil., *Schkuhria pinnata* var. *pinnata*	Dwarf marigold (E), Luswielo (V)	Asteraceae	Herb	Leaves, roots	Roots or leaves are chewed and swallowed for dysmenorrhoea	Mahwasane et al. (2013)	1

Botanical name	*Common name	Family	Life-form	#Part used	Application	Reference	Nm
Schotia afra (L.) Thunb. Synonym: *Guaiacum afrum* L.	Karoo boer-bean (E), Karoohuilboerboon (A)	Fabaceae	Tree	Leaves	Leaf infusion used to treat stomach ailment	Hulley and Van Wyk (2019)	1
Schotia brachypetala Sond. Synonym: *Schotia semireducta* Merxm.	Weeping boer-bean, tree fuchsia, African walnut (E), huilboerboon (A), umfofofo, ishimnumyane (X), molope (NS), mulibi, mununzwa, mununzwu, mutanswa (V), umutwa (TW), ihluze, umgxamu, uvovovo (Z)	Fabaceae	Tree	Bark, leaves, roots, seeds	For treating backache. Root decoction is used for shoulder pains. Seed decoction is used for stermum pains	Mintsa Mi Nzue (2009); Tshikalange et al. (2016)	2
Sclerocarya birrea (A.Rich.) Hochst. subsp. *caffra* (Sond.) Kokwaro. Synonyms: *Sclerocarya birrea*; *Sclerocarya caffra*, *Sclerocarya schweinfurthiana*	Cider tree, marula, maroola (E), maeroola, maroelaboom (A), Mufula (V), umganu (Z)	Anacardiaceae	Tree	Bark	Bark decoctions are used for abdominal pain. The bark for is used for headaches, toothache (rinsing oral cavity) and backache	Hutchings et al. (1996); Mabogo (1990); Watt and Breyer-Brandwijk (1962); Arnold and Gulumian (1984)	4
Searsia lancea (L.f.) F.A.Barkley. Synonyms: *Rhus lancea* L.f., *Toxicodendron lanceum* (L.f.) Kuntze	karee (E), karee, rooikaree (A), mokalabata, monhlohlo, motshakhutshakhu (NS), mosinabele, mosilabele (SS), mosabele, mosilabele (TW), mushakaladza (V), umhlakotshane (X)	Anacardiaceae	Tree	Bark	Bark infusion used to treat stomachache	Hulley and Van Wyk (2019)	1
Searsia leptodictya (Diels) T.S.Yi, A.J.Mill. & J.Wen. Synonyms: *Rhus leptodictya* Diels, *Rhus rhombocarpa* A. Fern. & R. Fern	Mountain karee, rock karee (E), bergkaree, klipkaree (A), Mohlwehlwe (NS), Mushakaladza (V)	Anacardiaceae	Tree	Bark, root	Smoke, inhaled under blanklet to relieve headache	Arnold and Gulumian (1984)	1
Searsia undulata (Jacq.) T.S.Yi, A.J.Mill. & J.Wen. Synonyms: *Rhus undulata* Jacq.,	Kuni-bush (E), taaibos, koeniebos, slapbos (A)	Anacardiaceae	Tree	ns	Infusions are used for backache	Hulley and Van Wyk (2019)	1

Botanical name	*Common name	Family	Life-form	#Part used	Application	Reference	Nm
Toxicodendron undulatum (Jacq.) Kuntze							
Secamone gerrardii Harv. ex Benth.	Ubuka (X), Inhlule-lemamba, Iphophoma, Ugobandlovu, Umhlonyise (Z)	Apocynaceae	Herb	Roots, bark	Roots and bark are used to relieve sharp chest pains	Hutchings et al. (1996)	1
Securidaca longepedunculata Fresen. Synonyms: *Elsota longipedunculata* (Fresen.) Kuntze, *Securidaca longipedunculata* var. *longipedunculata*	Violet tree (E), Langboslaagboom (A), Mpesu (V)	Polygalaceae	Tree	Bark, roots, root-kernel	Bark and roots are taken orally as infusions and decoctions for rheumatism. Root-kernel is used to treat headache. Root decoction is drunk thrice daily to relieve backache	Van Wyk and Gericke (2000); Watt and Breyer-Brandwijk (1962); Mongalo and Makhafola (2018); Arnold and Gulumian (1984)	4
Senecio cinerascens Aiton. Synonyms: *Cineraria seminuda* Klatt ex Schinz, *Senecio tomentosus* Salisb.	Handjiebos (A), Vieroulap, oulap (KS)	Asteraceae	Herb	Leaves	Used as compress for headache. Leaf infusion (or compress) used topically for pains	Nortje and van Wyk (2015)	1
Senecio serratuloides DC. Synonyms: *Senecio serratuloides var. glabratus* (DC.) DC., *Senecio serratuloides* var. *serratuloides*	Two-day Cure (E), Unsukumbili, Unsukumbili wehlathi (Z)	Asteraceae	Herb	ns	Analgesic (general pains), Musculo-skeletal (inflammation)	Mhlongo and Van Wyk (2019)	1
Senecio speciosus Willd. Synonyms: *Senecio concolor* DC., *Senecio concolor* var. *concolor*	Idambiso (X), Sebea-mollo-se-senye-nyane (SS) Inzwabuhlungu, Ibohlololo, Idambiso (Z),	Asteraceae	Herb	Leaves	Inflammations	Maroyi (2017)	1
Senegalia burkei (Benth.) Kyal. & Boatwr. Synonym: *Acacia burkei* Benth.	Black monkey thorn (E), swartapiesdoring (A), umkhaya, whelalahlatini (Z)	Fabaceae	Tree	Bark, roots	Bark and roots are used for eye and back complaints	Pooley (1993)	1
Senegalia caffra (Thunb.) P.J.H. Hurter & Mabb.	Hook-thorn, Acacia caffra (E), Gewone haakdoring	Fabaceae	Tree	ns	Used for toothache	Mbanjwa (2020)	1

Botanical name	*Common name	Family	Life-form	#Part used	Application	Reference	Nm
	(A), Mositsana (NS) Isakhamuzi, Umthole, Umtholo (Z)						
Senna didymobotrya (Fresen.) H.S.Irwin & Barneby. Synonyms: *Cassia nairobensis* Hort. ex L. H. Bailey, *Cassia verdickii* De Wild.	African senna, popcorn senna, candelabra tree, peanut butter cassia (E), motokomane (SP)	Fabaceae	Shrub	Roots	The roots are boiled and the extract drank as treatment for a painful body	Mogale et al. (2019)	1
Senna obtusifolia (L.) H.S.Irwin & Barneby. Synonyms: *Cassia contorta* Vogel, *Diallobus falcatus* Raf.	Chinese senna, sicklepod (E), Mutsheketsheke (V)	Fabaceae	Herb	Roots	Roots are boiled and the mixture drank for treating dysmenorrhoea	Mahwasane et al. (2013)	1
Senna petersiana (Bolle) Lock. Synonym: *Cassia petersiana* Bolle	Dwarf Cassia, eared Cassia, Eared Senna, Monkey Pod, Monkey Senna (E), Apiespeul (A), Bohlôko (NS), Munembenembe (V), Umnembenembe (Z)	Fabaceae	Shrub	Roots	Roots are boiled and the mixture drank for treating dysmenorrhoea. Root decoction is used to rinse the oral cavity to relieve toothache	Mahwasane et al. (2013); Arnold and Gulumian (1984)	2
Seriphium plumosum L. Synonyms: *Stoebe plumosa* (L.) Thunb., *Artemisia vermiculata* L.	Silver stoebe (E), slangbos, vaalbossie, Khoi-kooigoed (A), Hotnotswortel (KS)	Asteraceae	Shrub	Leaves	Leaf infusion used for treatment of backache	Nortje and van Wyk (2015)	1
Solanum aculeastrum Dunal. Synonym: *Solanum aculeastrum* var. *aculeastrum*	Apple of Sodom, bitter apple, devil's apple (E), Bitterappeltjie, Bokappel (A), Morola (NS), Murulwa, Shulwa (V), Intuma, Intuma, Intuma enkulu, Intumayezibaya, Uthuma, Untumane (Z)	Solanaceae	Shrub	Fruit, roots, ns	Ash from burnt fruit is rubbed into scarifications over painful parts for the relief of rheumatism pains. Fruits applied topically for toothache and are also placed in the wound after tooth extraction. Fruit decoctions are used as enemas for pain in the lower back and legs while ash is used for pains from walking. Analgesic	Watt and Breyer-Brandwijk (1962); Hutchings et al. (1996); Mhlongo and Van Wyk (2019); Mongalo and Makhafola (2018); Philander (2011); Mintsa Mi Nzue (2009); Mbanjwa (2020)	7

**Botanical name**	***Common name**	**Family**	**Life-form**	**#Part used**	**Application**	**Reference**	**Nm**
					(toothache, general pains, back aches), musculo-skeletal (inflammation). Roots are used to treat stomachache		
Solanum aculeatissimum Jacq. Synonyms: *Solanum cavaleriei* H. Lév. & Vaniot, *Solanum khasianum* C.B. Clarke	Apple of Sodom (E), ituma elikhulu, umthuma (Z)	Solanaceae	Shrub	Root, whole plant	Root decoctions are taken for backache and steam from burning plants is used for toothache	Hutchings et al. (1996)	1
Solanum americanum Mill. Synonyms: *Solanum nigrum* L., *Solanum adventitium* Polgar	Black berry, woody night shade (E), isihlalakuhle, udoye, ugqumgqumu, ugwabha, umaguqa (Z)	Solanaceae	Herb	Fruit, leaves, roots	Roots are used for the rheumatic pain. Fruit and leaves are used for abdominal pain and inflammation of the bladder	Hutchings et al. (1996)	1
Solanum capense L.	Nightshade (E), Monyaku (SS), Umthumana (X), Isithumana (Z)	Solanaceae	Shrub	Fruits	Powdered fruit is inserted into cavities for toothache and also into wounds after tooth extraction	Gerstner (1941)	1
Solanum elaeagnifolium Cav. Synonyms: *Solanum dealbatum* Lindl., *Solanum obtusifolium* Dunal	Silver-leaf nightshade (E), satansbos (A)	Solanaceae	Shrub	Fruits	The fruit is burned on the fire and the smoke is taken into the mouth to take away pain in the teeth	Corrigan et al. (2011)	1
Solanum hermannii Dunal. Synonyms: *Solanum sodomeum* var. *hermannii* Dunal, *Solanum sodomeum*	Bitter apple (E), Umthuma (Z)	Solanaceae	Shrub	Roots, leaves, fruits	Root infusions and decoctions are used for abdominal pain. Leaf decoctions are taken for backache. Crushed fruit is used for toothache	Hutchings et al. (1996); Watt and Breyer-Brandwijk (1962)	2
Solanum incanum L. Synonyms: *Solanum bojeri* Dunal, *Solanum sanctum* L.	Bitter Apple, Sodom Apple (E), Intuma, Intuma encane, Umagangeni (Z)	Solanaceae	Herb	Fruits, roots, ns	Analgesic (lower back aches, toothache). Fruits and roots are used for treating toothache and sprain	Mhlongo and Van Wyk (2019); Mbanjwa (2020)	2

Botanical name	*Common name	Family	Life-form	#Part used	Application	Reference	Nm
Solanum marginatum L.f.	Purple African nightshade (E), Intuma (Z)	Solanaceae	Shrub	ns	Analgesic (toothache), musculo-skeletal (inflammation)	Mhlongo and Van Wyk (2019)	1
Solanum mauritianum Scop. Synonyms: *Solanum tabacifolium* Vell., *Solanum verbascifolium* var. *auriculatum* (Aiton) Kuntze	Bugweed (E), Mothollo (NS), Umbhangabhanga, Ugwayana (Z)	Solanaceae	Shrub	Roots, ns	Used to mitigate ear-ache. Roots are used to treat stomachache	Mhlongo and Van Wyk (2019); Mongalo and Makhafola (2018)	2
Solanum panduriforme E. Mey.	Morolana (NS), Mututulwa (V), Intuma encane (Z)	Solanaceae	Herb	Roots, fruits, ns	Analgesic (toothache), musculo-skeletal (inflammation). Roots are used to treat stomachache, Fruits are burned and powder is applied externally on incision made on forehead to relieve headache	Mhlongo and Van Wyk (2019); Mongalo and Makhafola (2018); Arnold and Gulumian (1984)	3
Solanum retrofractum L = *Lycianthes stellata Bitter*	Nastergal, nasgal (A)	Solanaceae	Herb	ns	Infusions used for treatment of inflammation	Hulley and Van Wyk (2019)	1
Solanum supinum Dunal. Synonyms: *Solanum aranoideum* Dammer, *Solanum lyratifolium* Dammer	Morola (NS)	Solanaceae	Herb	Roots	Roots are used to treat stomachache	Mongalo and Makhafola (2018)	1
Solanum tomentosum L.	Slangappelbos (E), gifappel, Vuilsiekbossie, Doringappeltjie, !nuheis, bitterboelabos (A), Tandpynbossie (KS)	Solanaceae	Shrub	Fruits, leaves	Fruit is used for toothache. Ground leaves is a treatment for backache and stomachache	Nortje and van Wyk (2015); De Beer and Van Wyk (2011); Hulley and Van Wyk (2019)	3
Solanum retroflexum Dunal	Nightshade (E), Muxe (V)	Solanaceae	Herb	Leaves	Squeezed liquid from leaves is used to treat earache in children.	Mokganya and Tshisikhawe (2019)	1
Sonchus asper (L.) Hill. Synonyms: *Sonchus aemulus*	Prickly Sowthistle, spiney Sowthistle, corky-bark (E), irhwabe (X)	Asteraceae	Herb	Leaves	Leaf decoction prepared from approximately two glasses of water allowed to	Bhat (2014)	1

Botanical name	*Common name	Family	Life-form	#Part used	Application	Reference	Nm
Merino, *Sonchus carolinianus* Walter					stand for 1 hour and taken orally to treat chronic stomachache		
Spermacoce natalensis Hochst. Synonyms:*Borreria natalensis* (Hochst.) K.Schum. ex S.Moore, *Diodia natalensis* (Hochst.) J.G.García	Isimuyisane, Isindiyandiya, Insulansula (Z)	Rubiaceae	Herb	ns	Used to treat painful neck	Mbanjwa (2020)	1
Spilanthes mauritiana (A.Rich. ex Pers.) DC.	Tshishengelaphofu (V), Isishoshokazane, Isisinini (Z)	Asteraceae	Creeper	ns	Analgesic (toothache)	Mhlongo and Van Wyk (2019); Mbanjwa (2020)	2
Spirostachys africana Sond. Synonyms: *Excoecaria africana* (Sond.) Müll.Arg., *Spirostachys synandra* (Pax) Pax	tamboti (E), tambotie (A), umThombothi (Zulu); Modiba (NS), Morukuru (TW), Muonze (V)	Euphorbiaceae	Tree	Bark/wood	Smoke of the burning wood is inhaled to relieve headache	Arnold and Gulumian (1984)	1
Stachys cuneata Banks ex Benth. Synonym: *Stachys denticulata* Burch. ex Benth.	Bergtee, vaaltee (A)	Lamiaceae	Herb	ns	For painful body	Van Wyk et al. (2008)	1
Stangeria eriopus (Kunze) Baill. Synonyms: *Stangeria katzeri* Regel, *Stangeria paradoxa* T.Moore	Natal Grass Cycad, Cycad (E), obbejaankos (A), Umfingwani, Umncuma (X), Imfingo, Umafinga (Z)	Zamiaceae	Shrub	Tubers, roots	Burnt powdered underground tubers are used for headaches. Tubers are used for pains in the bones. Analgesic (sharp internal body pains). Roots used to treat headache	Hutchings et al. (1996); Watt and Breyer-Brandwijk (1962); Mhlongo and Van Wyk (2019); Coopoosamy and Naidoo (2012)	4
Stapelia gigantea N.E.Br. Synonyms: *Gonostemon giganteus* (N.E.Br.) P.V.Heath, *Stapelia meintjesii* Verd.	Giant carrion flower, Giant Stapelia (E), Reeuseaasblom (A), ililo elikhulu, isihlehle, uzililo (Z)	Apocynaceae	Herb	Whole plant	Ash from burnt plants is rubbed into scarifications made on the body or limbs to relieve pain	Watt and Breyer-Brandwijk (1962)	1
Stapelia nobilis N.E.Br. ex Hook.f. Synonym: *Gonostemon*	Madadza shango (V)	Apocynaceae	Herb	Roots	Root decoction is prepared and the body is exposed to	Arnold and Gulumian (1984)	1

Botanical name	*Common name	Family	Life-form	#Part used	Application	Reference	Nm
giganteus var. *nobilis* (Hook.f.) P.V.Heath					the steam to relieve general body ache		
Stoebe capitata P.J.Bergius. Synonyms: *Gymnachaena bruniades* Rchb. ex DC., *Stoebe bruniades* (Rchb.) Levyns	Groen slangbos (A)	Asteraceae	Shrub	Leaves	Leaves are used for treating stomach and menstrual complaints	Philander (2011)	1
Stoebe incana Thunb. = *Seriphium incanum* (Thunb.) Pers. Synonym: *Stoebe leiocarpa* Sch.Bip.	Vit moerbos (A)	Asteraceae	Shrub	Leaves	Leaves are used for treating menstrual complaints	Philander (2011)	1
Stoebe plumosa (L.) Thunb. = *Seriphium plumosum* L.	silver stoebe (E), slangbos, vaalbossie, Khoi-kooigoed, Grys slangbos (A)	Asteraceae	Shrub	Leaves	Leaves are used for treating stomach and menstrual complaints	Philander (2011)	1
Strychnos cocculoides Baker. Synonyms: *Strychnos paralleloneura* Gilg & Busse, *Strychnos suberosa* De Wild.	Corky-bark monkey-orange (E), kurkbasklapper (A), morapa (NS), umnonono (X)	Loganiaceae	Tree	Bark	Approximately two spoons grated bark is mixed with about 2 glasses of water which is allowed to stand for 1 hour and taken orally to treat stomachache	Bhat (2014)	1
Strychnos henningsii Gilg. Synonyms: *Strychnos albersii* Gilg & Busse.; *Strychnos holstii* Gilg	Coffee bean Strychnos, Natal teak, coffee hard pear (E), harclepeer(hout), rooibitterbessie (A), umanana, umdunye, umnono, umqalothi, umqaloti (Z)	Loganiaceae	Shrub	Roots, bark	Boiled roots are used for stomach complaints. Bark decoctions boiled with roots of *Turraea floribunda* Hochst are taken for the pains of rheumatic fever. Bark is used in the treatment of dysmenorrhoea. Analgesic (body pains)	Hutchings et al. (1996); Watt and Breyer-Brandwijk (1962): Mhlongo and Van Wyk (2019); Philander (2011)	4
Strychnos madagascariensis Poir. Synonyms: *Strychnos burtonii* Baker, *Strychnos unguacha* var. *micrantha* Gilg	Black monkey orange (E); swartklapper, botterklapper (A), Morapa (NS), Mogorwagorwana (TW), Mukwakwa (V),	Loganiaceae	Tree	Roots	Roots are used to treat footache. Roots are roasted and sap is dropped on tooth to relieve toothache	Mongalo and Makhafola (2018); Arnold and Gulumian (1984)	2

Botanical name	*Common name	Family	Life-form	#Part used	Application	Reference	Nm
	umGluguza, umKwakwa (Z)						
Sutherlandia frutescens (L.) R.Br. = *Lessertia frutescens* (L.) Goldblatt & J.C.Manning subsp. *frutescens*. Synonym: *Colutea frutescens* L.	Turkey flower, balloon pea, cancer bush (E), Wildekeur (A), Kankerbossie (KS), Umnwele (X), Umnwele, Unwele (Z)	Fabaceae	Herb	leaves, fruits, seeds, stem, flowers, whole plant	Different parts of the plant are used for treating backache and rheumatism. Leaf wash used for painful feet. Chewed in the mouth and placed on tooth to help with toothache. Infusion is used to treat backache and stomach ailments	Watt and Breyer-Brandwijk (1962); Nortje and van Wyk (2015); De Beer and Van Wyk (2011); Hulley and Van Wyk (2019); Thring and Weitz (2006); Mintsa Mi Nzue (2009)	6
Sutherlandia microphylla Burch.	Wildekeur, kalkoenbel, kankerbos (A)	Fabaceae	Herb	Leaves	Leaves are used for back pain, stomach pain and kidney pain	Van Wyk et al. (2008)	1
Symphytum officinale L. Synonyms: *Consolida major* Garsault, *Symphytum peregrinum* Ledeb.	Comfrey (E)	Boraginaceae	Herb	Leaves	Young leaves are eaten and wrapped around sprains to relieve pains	Philander (2011)	1
Synadenium cupulare L.C. Wheeler = *Euphorbia cupularis* Boiss. Synonym: *Synadenium arborescens* Boiss.	Crying Tree, Dead-man's Tree (E), Dooiemansboom, Gifboom (A), Mulamba-noni, muswoswo (V), Umdlebe, Umdletshane (Z)	Euphorbiaceae	Tree	Roots	Burned roots are mixed with petroleum jelly and rubbed on swollen ankle	Arnold and Gulumian (1984)	1
Syzygium cordatum Hochst.ex C.Krauss. Synonyms: *Syzygium cymiferum* (E.Mey.) C.Presl, *Jambosa cymifera* E.Mey.	Water Wood, water berry (E), waterbessie (A), Mutu (V), umswi, umjomi (X), Umdoni (Z)	Myrtaceae	Tree	Bark, leaves, roots	The Bemba use cold leaf infusions for various stomach ailments. Vhavenda use leaves for fever while bark and roots are used for headache. For treating inflammation. Roots are burned and the ash is applied	Hutchings et al. (1996); Watt and Breyer-Brandwijk (1962); Mabogo (1990); Maroyi (2017); Arnold and Gulumian	6

Botanical name	*Common name	Family	Life-form	#Part used	Application	Reference	Nm
					on incisions on forehead to relieve headache. Used for relieving menstrual pain	(1984); Mbanjwa (2020)	
Tabernaemontana elegans Stapf. Synonyms: *Conopharyngia elegans* (Stapf) Stapf, *Leptopharyngia elegans* (Stapf) Boiteau	toad tree (E), laeveldse paddaboom (A), mahatu (V), umKhahlwana, umKhadlu (Z)	Apocynaceae	Tree	Roots	Root decoction is used to prepare soft porridge to relieve stomachache. Root maceration is taken to relieve stomachache. Root maceration is used to prepare porridge for 1 month to relieve stomachache in women. Maceration of root powder is drunk or used to prepare sot porridge to relieve stomachache in women. Exposure to burnt root under a blanket to relieve headache	Arnold and Gulumian (1984)	1
Tagetes minuta L. Synonyms: *Tagetes bonariensis* Pers., *Tagetes glandulosa* Schrank ex Link	Khaki weed, African marigold (E), Mushushathuri, mukakambanzhe (V), Ikhambi lempaka, Insangu katikoloshe, Usangwana (Z)	Asteraceae	Herb	Leaves, ns	Leaves are buned and the smoke inhaled for headache. Analgesic (toothache)	Mhlongo and Van Wyk (2019); Arnold and Gulumian (1984)	2
Tanacetum parthenium (L.) Sch.Bip. Synonyms: *Chrysanthemum parthenium* (L.) Pers., *Parthenium matricaria* Gueldenst.	Fever few (E), ubushwa (X)	Asteraceae	Herb	Leaves	Drop of the leaf decoction is placed in infected ear 2-3 times daily to treat inflammation of the ear	Bhat (2013)	1
Tecoma capensis (Thunb.) Lindl. Synonyms: *Tecomaria capensis* (Thunb.) Spach, *Bignonia capensis* Thunb.	Cape honeysuckle (E), Kaapse kamperfoelie, trompetters (A), Icakatha, Umsilingi (X), uchahacha,	Bignoniaceae	Shrub	Bark	Dried powdered bark infusions are taken for fevers, pain and stomach pains. Relieve pain and	Watt and Breyer-Brandwijk (1962); Mhlongo	2

Botanical name	*Common name	Family	Life-form	#Part used	Application	Reference	Nm
	Incwincwi, Uthswala benyoni (Z)				induce sleep and is rubbed on bleeding gums. Analgesic (backaches)	and Van Wyk (2019)	
Teedia lucida (ex Sol.) Rudolphi	Stinkbos, Glossy Lilac Berry (E), Klipdruiwe (KS), Hlwenya (Z)	Scrophulariaceae	Shrub	ns	Infusion is a remedy for inflammation	Nortje and van Wyk (2015)	1
Terminalia phanerophlebia Engl. & Diels	Lebombo cluster-leaf (E), Lebombotrosblaar (A), amaNgwe-amnyama, amaNgwe-omphofu (Z)	Combretaceae	Tree	Roots	Roots are rubbed into scarifications for rheumatic ailments	Watt and Breyer-Brandwijk (1962)	1
Terminalia sericea Burch. ex DC. Synonym: *Terminalia angolensis* O.Hoffm.	silver cluster-leaf (E), silwerboom, Vaalboom (A), arnangwe (Z)	Combretaceae	Tree	Roots	For treating different form of pain in the chest, neck or shoulders.	Watt and Breyer-Brandwijk (1962)	1
Tetradenia riparia (Hochst.) Codd. Synonyms: *Basilicum riparium* (Hochst.) Kuntze, *Plectranthus riparius* Hochst.	misty plume bush, ginger bush (E), gemmerbos, watersalie (A), Iboza, Ibozane (E)	Lamiaceae	Herb	ns	Used for treating back pain	Mbanjwa (2020)	1
Tetragonia tetragonioides (Pall.) Kuntze. Synonyms: *Demidovia tetragonoides* Pall., *Tetragonia borealis* Batt. & Trab.	New Zealand spinach (E), Ibohlololo elimhlophe (Z)	Aizoaceae	Herb	ns	Musculo-skeletal (inflammation). Used as poltices for swollen feed	Mhlongo and Van Wyk (2019); Mbanjwa (2020)	2
Teucrium africanum Thunb. Synonym: *Ajuga africana* (Thunb.) Pers.	Aambeibossie, Bitterbos, Katjiedriedoring, Paddaklou, drievingertee, katjie-drie-blaar (A)	Lamiaceae	Shrub	ns	Used for the treatment of stomach ailments	Hulley and Van Wyk (2019)	1
Teucrium trifidum Retz. Synonyms: *Ajuga capensis* (Thunb.) Pers., *Teucrium capense* Thunb.	Aambeibossie, Akkedispoot, Koorsbossie, Akkedispootjie, katjiedrieblaar (A)	Lamiaceae	Herb	ns	For treating back pain	Van Wyk et al. (2008)	1
Thesium lineatum L. f. Synonyms: *Thesium ephedroides* A.W. Hill, *Thesium rigidum* Sond., *Thesium sparteum* R.Br.	Black storm (E), sawrtstrom, Vaalstorm, Witstorm (A)	Santalaceae	Shrub	Roots	Roots are burnt to relieve headache	Philander (2011)	1

**Botanical name**	***Common name**	**Family**	**Life-form**	**#Part used**	**Application**	**Reference**	**Nm**
Toddalia asiatica (L.) Lam. Synonyms: *Cranzia asiatica* (L.) Kuntze, *Toddalia aculeata* (Sm.) Pers.	Orange climber (E), Gwambazi (V)	Rutaceae	Shrub	Roots	Root decoction is drunk twice daily for heart pains and backache	Arnold and Gulumian (1984)	1
Trema orientalis (L.) Blume. Synonyms: *Celtis commersonii* Brongn., *Sponia commersonii* Decaisne ex Planchon	Trema, pigeon wood (E), hophout (A), umSekeseke, umBhangabhanga, umBengebenge	Cannabaceae	Tree	ns	Infusion used for toothache	Coopoosamy and Naidoo (2012)	1
Trichilia dregeana Sond. Synonym: *Trichilia strigulosa* Welw. ex C.DC.	Natal forest mahogany (E), Bosrooiessenhout (A), Mutshikili, Mutuhu, Muuhu (V), umathunzini, umkhuhla, Igxolo, umkhuhlu (Z)	Meliaceae	Tree	Bark	Bark decoctions are administered as enemas for backache associated with kidney problems. Unspecified parts are used for stomach complaints and backache. Analgesic (lower back aches, toothache)	Hutchings et al. (1996); Watt and Breyer-Brandwijk (1962); Mhlongo and Van Wyk (2019)	3
Trichilia emetica Vahl. Synonyms: *Elcaja emetica* Forssk., *Geniostephanus tomentosus* Fenzl	Cape/Natal mahogany, reel ash, thunder tree(E), basteresse(n)hout (A), Mutshikili, Mutuhu (V), umkhuhlwa (Z)	Meliaceae	Tree	Seeds, bark, roots	Oil from seeds taken orally for rheumatism. Bark decoction is used as enema to treat pains in the waist in men. Root infusion is used for treating painful feet	Watt and Breyer-Brandwijk (1962); Arnold and Gulumian (1984); Tshikalange et al. (2016)	3
Tulbaghia alliacea L.f. Synonyms: *Omentaria alliacea* (L.f.) *Kuntze, Tulbaghia brachystemma* Kunth	wild garlic (E), wildeknotflok (A), ishaladi-lezinyoka, knoffel (KS), isikhwa, umwelela (Z)	Amaryllidaceae	Herb	Rhizomes	Infusions are also used by for rheumatism	Watt and Breyer-Brandwijk (1962); Nortje and van Wyk (2015)	2
Tulbaghia capensis L. Synonyms: *Omentaria capensis* (L.) Kuntze, *Tulbaghia cepacea* L.f., *Tulbaghia pulchella* Avé-Lall.	Wild garlic (E), wilde knoffel (A)	Amaryllidaceae	Herb	Bulbs	Treatment for arthritis	Philander (2011)	1

Botanical name	*Common name	Family	Life-form	#Part used	Application	Reference	Nm
Tulbaghia violacea Harv. Synonyms: *Omentaria alliacea* (L.f.) Kuntze, *Tulbaghia brachystemma* Kunth	Wild garlic (E), wildeknofflok, wildeknoffel, bergknoffe (A), isihaqa (Z)	Amaryllidaceae	Herb	Tubers/bulbs, roots, leaves	Pounded tuber decoctions are administered as enemas for stomach ailments. Leaves are rubbed on the head for sinus headache. Administered in enemas for rheumatism. Clove pieces are placed in castor oil to make eardrops. Used for stomach ailments	Watt and Breyer-Brandwijk (1962); Hutchings et al. (1996); Hulley and Van Wyk (2019); Thring and Weitz (2006); Mintsa Mi Nzue (2009)	5
Turbina oblongata A. Meeuse. Synonyms: *Ipomoea lambtoniana*, *Ipomoea oblongata* E. Mey. ex Choisy	Honeysuckle Tree (E), ubhoqo (Z)	Convolvulaceae	Herb	Leaves, roots	Leaves are used as poultices for swollen joints, sores and abscesses. Taken internally for rheumatism and gout. Ground root decoctions are taken three times a day for arthritis and gout. Enemas made from roots are given for pain of the spine	Hutchings et al. (1996); Pujol (1990); Polori et al. (2018)	3
Turraea floribunda Hochst. Synonym: *Rutaea floribunda* (Hochst.) M.Roem.	Honeysuckle Tree (E), Kanferfoelieboom (A), Umadlozana (X), Ubhugulo, Umadlozana, Umadlozane (Z)	Meliaceae	Tree	Roots	Root infusions are taken for rheumatism. Root decoctions are taken as emetics to treat swollen and painful joints	Watt and Breyer-Brandwijk (1962); Pujol (1990)	2
Urtica dioica L. Synonyms: *Urtica dioica* var. *vulgaris* Wedd., *Urtica galeopsifolia* Wierzb. ex Opiz	Nettle (E)	Urticaceae	Herb	Leaves	Leaves are applied topically for arthritis and rheumatism	Philander (2011)	1
Urtica urens L. Synonym: *Urtica trianae* Rusby	Annual nettle, dwarf nettle, small nettle, dog nettle, burning nettle (E), perdebrandnekel, brandnekel (A)	Urticaceae	Herb	ns	Infusion used for toothache, ear-ache, pain and inflammation. Remedy for treating rheumatism	Hulley and Van Wyk (2019)	1
Vachellia karroo (Hayne) Banfi & Galasso. Synonyms: *Acacia*	Sweet thorn (E), soetdoring, doringboom	Fabaceae	Tree	Bark, thorns,	Analgesic (sharp internal body pains). Bark is used to	Mhlongo and Van Wyk (2019); De	5

Botanical name	*Common name	Family	Life-form	#Part used	Application	Reference	Nm
karroo, *Acacia inconflagrabilis* Gerstner	(A), mooka (TW), Muunga (V), Ingamazi, Ingamazi elincane, Umunga, Umantungane, Usidlodlo (Z)			leaves, roots, ns	treat aching legs. Used for stomachache. Thorn decoction is drunk to relieve heart pains. Gum is eaten for stomach ailments and toothache. Bark infusion used to treat stomachache, pain and inflammation. Root infusion is used to treat swollen and burning feet	Beer and Van Wyk (2011); Watt and Breyer-Brandwijk (1962); Arnold and Gulumian (1984); Hulley and Van Wyk (2019)	
Vachellia natalitia (E.Mey.) Kyal. & Boatwr. Synonym: *Acacia natalitia* E.Mey.	Natal Thorn (E), Natal-doring (A), Isingqawe, Isinqawe, Umunga, Umnqawe (Z)	Fabaceae	Tree	ns	Analgesic (sharp internal body pains)	Mhlongo and Van Wyk (2019)	1
Vachellia nilotica (DC.) Kyal. & Boatwr. subsp. *kraussiana* (Benth.) Kyal. & Boatwr. Synonyms: *Acacia arabica* var. *nilotica* (L.) Benth., *Acacia scorpioides* var. *nilotica* (L.) A. Chev.	Scented-pod acacia (E), lekkerruikpeul (A), Motsha (TW), Isambulela, Isingqawe, Isinqawe, Umunga (Z)	Fabaceae	Tree	Roots, ns	Analgesic (sharp internal body pains). Root decoction is used for back pains	Mhlongo and Van Wyk (2019); Tshikalange et al. (2016)	2
Vachellia sieberiana (DC.) = *Vachellia sieberiana* (L.) P.J.H. Hurter & Mabb. var. woodii (Burtt Davy) Kyal. & Boatwr.Kyal. & Boatwr. Synonyms: *Acacia sieberiana*, *Acacia amboensis*	paperbark thorn (E), Mokha, Morumosetlha (TW), mavelo (V), Isinqawe esimphlope, Umkhamba, Uselephe (Z)	Fabaceae	Tree	Bark, ns	Bark infusions are administered as enemas for back pain. Analgesic (sharp internal body pains)	Watt and Breyer-Brandwijk (1962); Mhlongo and Van Wyk (2019)	2
Vangueria infausta Burch. Synonyms: *Canthium infaustum* (Burch.) Baill., *Vangueria tomentosa* Hochst.	wild medlar (E); wilde mispel (A), Mabilo (SL), Amaviyo, Umtulwa (Z)	Rubiaceae	Tree	ns, bark, roots	Analgesic (internal side pains), chest side pains in infants. Bark and roots are medicine for alleviating toothache. Used as steam bath for treating painful body	Mhlongo and Van Wyk (2019); Shai et al. (2020); Mbanjwa (2020)	3

Botanical name	*Common name	Family	Life-form	#Part used	Application	Reference	Nm
Vangueria lasiantha (Sond.) Sond. Synonyms: *Lagynias discolor* E.Mey. ex Robyns, *Pachystigma lasianthum* Sond.	Smooth Pendent-medlar (E), Amatulwa, Umtulw	Rubiaceae	Tree	Leaves	Leaves are used for steaming to cure painful body	Mbanjwa (2020)	1
Veltheimia capensis (L.) DC. Synonyms: *Aletris capensis* L., *Fabricia glauca* (Aiton) Salisb.	quarobe, sand lily (E), kwarobe, sandlelie (A)	Asparagaceae	Herb	ns	Used for the treatment of stomach-related ailments	Hulley and Van Wyk (2019)	1
Vepris undulata Verdoorn & C. A. Sm. Synonym: *Toddalia lanceolata* Lam.	white ironwood (E), witysterhout (A), Muhondwa, muumano, phaladzane (V), umZane (X), umOzana (Z)	Rutaceae	Tree	Leaves	Powder leaves is used externally on incision made on forehead to relieve headache	Arnold and Gulumian (1984)	1
Veronica anagallis-aquatica L. Synonyms: *Veronica anagallidiformis* Boreau, *Veronica brittonii* Porter ex Pennell	water speedwell (E), waterboege (A)	Plantaginaceae	Herb	Leaves	Infusion from the leaves is used to treat backache, wash painful legs and feets	De Beer and Van Wyk (2011)	1
Viscum capense L. f. Synonyms: *Viscum rigidum* Engler & Krause, *Viscum robustum* Eckl. & Zeyh.	Cape Mistletoe (E), Voëlent, litjiestee (A)	Santalaceae	Herb	ns	Infusion is used to treat headache	Hulley and Van Wyk (2019)	1
Vitex obovata E.Mey. Synonyms: *Vitex reflexa* H.Pearson, *Vitex wilmsii* Gürke	Hairy Fingerleaf (E), Vaalbos (A), umduli, umluthu (Z)	Lamiaceae	Tree	Leaves	Leaf infusions are administered as enemas for stomach ache	Watt and Breyer-Brandwijk (1962)	1
Volkameria glabra (E.Mey.) Mabb. & Y.W.Yuan. Synonyms: *Clerodendrum glabrum* E.Mey, *Siphonanthus glaber* (E.Mey.) Hiern, *Premna suaveolens* Chiov.	Tinderwood, verbena tree, white eat's whiskers (E), tontelhout, bitterblaar, bontelhout, harpuisblaar, huilboom (A), munukha-tshilongwe (V), umqangazane, umqaqongu, umqoqongo (Z)	Lamiaceae	Shrub	Leaves, roots, ns	Hot water infusions from roots, mixed with those of *Tetradenia riparia* are taken as emetics for dropsy and rheumatic conditions. Leaves are used for toothache. Analgesic (toothache)	Watt and Breyer-Brandwijk (1962); Hutchings et al. (1996); Mhlongo and Van Wyk (2019)	3

Botanical name	*Common name	Family	Life-form	#Part used	Application	Reference	Nm
Warburgia salutaris (G.Bertol.) Chiov. Synonyms: *Chibaca salutaris*, *Warburgia breyeri* R.Pott	Fever tree, pepper-bark tree (E), koorsboom, peperbasboom (A), mulanga (V), amazwecehlabayo, isibaha, isibhaha (Z)	Canellaceae	Tree	Bark, leaves, roots	Bark is used in emetics or purgatives for febrile complaints and for rheumatism. Lotions made from pounded leaves with stalks of *Hibiscus surattensis* are applied to the penis for inflammation of the urethra, sores and other irritation. Powdered roots are applied on oral cavity to relieve toothache. Decoction of the bark is taken for backache	Hutchings et al. (1996); Corrigan et al. (2011); Arnold and Gulumian (1984)	3
Withania somnifera (L.) Dunal. Synonyms: *Physalis somnifera*, *Withania microphysalis*	Wilde-appelliefie (A), Winter Cherry, Poisonous Gooseberry (E), Ubuvuma (X), Impathampatha, Ubuvimba Umaqhunsula, Ubuvumba (Z)	Solanaceae	Shrub	Leaves, roots	Leaf poultices are applied externally to treat rheumatism. Musculo-skeletal (inflammation). For treating inflammation. Leaf infusions are used to treat stomach ailments	Hutchings et al. (1996); Mhlongo and Van Wyk (2019); Maroyi (2017); Van Wyk et al. (2008); Hulley and Van Wyk (2019)	5
Ximenia americana L. Synonyms: *Amyris arborescens* P. Browne, *Ximenia americana* var. *americana*	Small blue sourplum (E), kleinblousuurpruim (A), morotologana (TW), hwele, mosidi-wa-serotologane (NS); mutanzwa (V), umtunduluka-omncane, umkholotshwana (Z)	Olacaceae	Tree	Roots	Roots are used in the treatment of stomachache	Mongalo and Makhafola (2018)	1
Ximenia caffra Sond.	Plum, large sourplum (E), Kleinsuurpruim (A), Mutanwadombo, Mutshili (V), Umthunduluka-obomvu (Z)	Olacaceae	Tree	Leaves, roots	Cold leaf infusions are applied to inflamed eyes. Root decoction is use to prepare soft porridge for headache due to indigestion	Watt and Breyer-Brandwijk (1962); Mabogo (1990); Arnold and Gulumian (1984)	3

Botanical name	*Common name	Family	Life-form	#Part used	Application	Reference	Nm
Xysmalobium undulatum (L.) W.T. Aiton Synonyms: *Asclepias ciliata* Murray ex Decne., *Asclepias undulata* L.	Milk bush, milkwort, uzura, wild cotton, wave-leaved xysmalobium (E), Ishongwe, Indonya (Z)	Apocynaceae	Herb	Roots, stem, ns	Musculo-skeletal (inflammation). For relieving headache	Mhlongo and Van Wyk (2019); Mintsa Mi Nzue (2009)	2
Zantedeschia aethiopica (L.) Spreng. Synonyms: *Arodes aethiopicum* (L.) Kuntze, *Colocasia aethiopica* (L.) Link	White or common arum lily (E); wit varkoor (A), Ingquthuyengane, Intebe (Z)	Araceae	Herb	Leaves, ns	Musculo-skeletal inflammation. Compress leaves is used for treating backache, rheumatism, headache, pain and inflammation. Treatment for arthritis	Mhlongo and Van Wyk (2019); Hulley and Van Wyk (2019); Philander (2011)	3
Zanthoxylum capense (Thunb.) Harv. Synonyms: *Fagara magaliesmontana* Engl., *Zanthoxylum thunbergii* var. *obtusifolia* Harv.	Adelaide spice tree, cardamom (E), kleinperdepram (A), Umabelejongosi, isinungwane, umlungumabele (Z)	Rutaceae	Tree	Leaves, bark (root-bark), roots	Leaves are used to heal sores. Dried ground root-bark is directly applied for toothache. Analgesic (general body pains). An infusion of the root is taken to treat toothache. For treating swollen feet and toothache	Bryant (1966); Hutchings et al. (1996); Mhlongo and Van Wyk (2019); Corrigan et al. (2011); Philander (2011); Mbanjwa (2020)	6
Zanthoxylum davyi Waterm. Synonyms: *Zanthoxylum thunbergii* var. *grandifolia* Harv., *Fagara davyi* I.Verd	Fever Tree, Forest Knobwood (E), Knopdoringhout (A), Monokwane (NS), Munungu, Murandela (V), Umlungamabele, Umlungumabele (X), Umnungumabele, Omkhulu (Z)	Rutaceae	Tree	Roots, leaves	For tooth removal. Root decoction is drunk thrice daily for 3 days to relieve chest pains. Powdered leaves are rubbed on chest to relieve pains	Mhlongo and Van Wyk (2019); Mabogo (1990); Mbanjwa (2020)	3
Ziziphus mucronata Willd. Synonyms: *Ziziphus madecassus* H. Perrier, *Ziziphus mucronata* subsp. *mucronata*	Blinkblaarboom (A) Buffalo thorn, Cat-thorn (E), Umphafa (X), Mutshetshete, Mukhalu (V), isilahla,	Rhamnaceae	Tree	Bark, roots, leaves	Bark decoctions are used for rheumatism. Roots are used for toothache. Leaves and roots are used for pain. Analgesic (sharp internal	Mabogo (1990); Mhlongo and Van Wyk (2019); Maroyi (2017);	4

Botanical name	*Common name	Family	Life-form	#Part used	Application	Reference	Nm
	umhlahlankosi, umphafa (Z).				body pains). For treating chest pain. Root decoction is used to prepare soft porridge to relieve general body pains	Arnold and Gulumian (1984)	
Zygophyllum foetidum Schrad. & Wendl. Synonym: *Zygophyllum meyeri* Sond	Jakkalspisbos, Jakkalspisbossie, Slymbos (A)	Zygophyllaceae	Shrub	Leaves	Leaf juice is used for treating ear-ache	Hulley and Van Wyk (2019)	1

References

Arnold, H.-J., Gulumian, M. 1984. Pharmacopoeia of traditional medicine in Venda. Journal of Ethnopharmacology 12:35-74.

Bhat, R.B. 2013. Plants of Xhosa people in the Transkei region of Eastern Cape (South Africa) with major pharmacological and therapeutic properties. Journal of Medicinal Plants Research 7:1474-1480.

Bhat, R.B. 2014. Medicinal plants and traditional practices of Xhosa people in the Transkei region of Eastern Cape, South Africa. Indian Journal of Traditional Knowledge 13:292-298.

Bhat, R.B., Jacobs, T.V. 1995. Traditional herbal medicine in Transkei. Journal of Ethnopharmacology 48:7-12.

Bruce, W. 1975. Medicinal properties in the Aloe. Excelsa 5: 57-68.

Bryant, A.T. 1966. Zulu medicine and medicine-men. C. Struik, Cape Town, South Africa.

Coopoosamy, R.M., Naidoo, K.K. 2012. An ethnobotanical study of medicinal plants used by traditional healers in Durban, South Africa." 6.11 (2012): 818 - 823. African Journal of Pharmacy and Pharmacology 6:818-823.

Corrigan, B.M., Van Wyk, B.-E., Geldenhuys, C.J., Jardine, J.M. 2011. Ethnobotanical plant uses in the KwaNibela Peninsula, St Lucia, South Africa. South African Journal of Botany 77:346-359.

Cumes, D., Loon, R., Bester, D. 2009. Healing Trees & Plants of the Lowveld. Struik Nature, Cape Town, South Africa.

De Beer, J.J.J., Van Wyk, B.E. 2011. An ethnobotanical survey of the Agter–Hantam, Northern Cape Province, South Africa. South African Journal of Botany 77:741-754.

Forbes, V.S. 1986. Carl Peter Thunberg Travels at the Cape of Good Hope 1772–1775. Van Riebeeck Society Cape Town, ISBN: 0620109815.

Gebashe, F., Moyo, M., Aremu, A.O., Finnie, J.F., Van Staden, J. 2019. Ethnobotanical survey and antibacterial screening of medicinal grasses in KwaZulu-Natal Province, South Africa. South African Journal of Botany 122:467-474.

Gerstner, J. 1941. A preliminary check list of Zulu names of plants, with short notes. Bantu Studies 13:277-301.

Hulley, I.M., Van Wyk, B.E. 2019. Quantitative medicinal ethnobotany of Kannaland (western Little Karoo, South Africa): Non-homogeneity amongst villages. South African Journal of Botany 122:225-265.

Hulme, M.M. 1954. Wild flowers of Natal. Shuter & Shooter, Pietermaritzburg, South Africa.

Hutchings, A., Scott, A.H., Lewis, G., Cunningham, A. 1996. Zulu Medicinal Plants. An Inventory. University of Natal Press, Pietermaritzburg, South Africa.

Mabogo, D.E.N. 1990. The ethnobotany of the Vhavenda. University of Pretoria.

Mahwasane, S.T., Middleton, L., Boaduo, N. 2013. An ethnobotanical survey of indigenous knowledge on medicinal plants used by the traditional healers of the Lwamondo area, Limpopo province, South Africa. South African Journal of Botany 88:69-75.

Maroyi, A. 2017. Diversity of use and local knowledge of wild and cultivated plants in the Eastern Cape province, South Africa. Journal of Ethnobiology and Ethnomedicine 13:10.1186/s13002-13017-10173-13008.

Mbanjwa, S.G. 2020. A quantitative ethnobotanical survey of the Ixopo area of KwaZulu-Natal,South Africa. University of Johannesburg, Johannesburg, South Africa.

Mhlongo, L.S., Van Wyk, B.E. 2019. Zulu medicinal ethnobotany: new records from the Amandawe area of KwaZulu-Natal, South Africa. South African Journal of Botany 122:266-290.

Mintsa Mi Nzue, A.P. 2009. Use and conservation status of medicinal plants in the Cape Peninsula, Western Cape Province of South Africa. University of Stellenbosch, Stellenbosch, South Africa.

Mogale, M.M.P., Raimondo, D.C., Van Wyk, B.-E. 2019. The ethnobotany of Central Sekhukhuneland, South Africa. South African Journal of Botany 122:90-119.

Mokganya, M.G., Tshisikhawe, M.P. 2019. Medicinal uses of selected wild edible vegetables consumed by Vhavenda of the Vhembe District Municipality, South Africa. South African Journal of Botany 122:184-188.

Mongalo, N.I., Makhafola, T.J. 2018. Ethnobotanical knowledge of the lay people of Blouberg area (Pedi tribe), Limpopo Province, South Africa. Journal of Ethnobiology and Ethnomedicine 14:46.

Nortje, J.M., van Wyk, B.E. 2015. Medicinal plants of the Kamiesberg, Namaqualand, South Africa. Journal of Ethnopharmacology 171:205-222.

Palmer, E., Pitman, N. 1961. Trees of South Africa, Balkema, Cape Town.

Philander, L.A. 2011. An ethnobotany of Western Cape Rasta bush medicine. Journal of Ethnopharmacology 138:578-594.

Polori, K.L., Mashele, S.S., Madamombe-Manduna, I., Semenya, S.S. 2018. Ethno-medical botany and some biological activities of ipomoea oblongata collected in the Free State Province, South Africa. Journal of Biological Sciences 18:441-449.

Pooley, E. 1993. The Complete Field Guide to Trees of Natal, Zululand and Transkei. 1st edition. Natal Flora Publications Trust, Durban, South Africa.

Pujol, J. 1990. NaturAfrica: The Herbalist Handbook: African Flora, Medicinal Plants. Natural Healers' Foundation, Durban, South Africa.

Shai, K.N., Ncama, K., Ndhlovu, P.T., Struwig, M., Aremu, A.O. 2020. An exploratory study on the diverse uses and benefits of locally-sourced fruit species in three villages of Mpumalanga Province, South Africa. Foods 9:1581.

Thring, T.S.A., Weitz, F.M. 2006. Medicinal plant use in the Bredasdorp/Elim region of the southern Overberg in the Western Cape Province of South Africa. Journal of Ethnopharmacology 103:261-275.

Tshikalange, T.E., Mophuting, B.C., Mahore, J., Winterboer, S., Lall, N. 2016. An ethnobotanical study of medicinal plants used in villages under Jongilanga tribal council, Mpumalanga, South Africa. African Journal of Traditional, Complementary and Alternative Medicines 13:83-89.

Van Wyk, B.-E., de Wet, H., Van Heerden, F.R. 2008. An ethnobotanical survey of medicinal plants in the southeastern Karoo, South Africa. South African Journal of Botany 74:696-704.

Van Wyk, B.-E., Gericke, N. 2000. People's Plants: A Guide to Useful Plants of Southern Africa. Briza Publications, Pretoria, South Africa.

Van Wyk, B.-E., Van Oudtshoorn, B., Gericke, N. 1997. Medicinal Plants of South Africa. First edition. Briza Publications, Pretoria, South Africa.

Watt, J.M., Breyer-Brandwijk, M.G. 1962. The Medicinal and Poisonous Plants of Southern and Eastern Africa. 2nd edition. Livingstone, London, UK.
